# Methodology for the selection and evaluation of outcomes for Chinese herbal injection in acute exacerbation of chronic obstructive pulmonary disease (AECOPD): a comprehensive study

**DOI:** 10.1080/07853890.2024.2396567

**Published:** 2024-09-10

**Authors:** De Zhao Kong, Si Hong Yang, Hui Zhao, Xiu Wei Hao, Tong Wu Zhang, Yi Lu

**Affiliations:** a Institute of Basic Research in Clinical Medicine, China Academy of Chinese Medical Sciences, Beijing, China; b China Center for Evidence-Based Traditional Chinese Medicine (CCEBTCM), China Academy of Chinese Medical Sciences, Beijing, China; c Liaoning University of Traditional Chinese Medicine, Shenyang, China

**Keywords:** Outcomes selection, clinical evaluation, AECOPD, methodology, Chinese herbal injection

## Abstract

**Objective:**

To develop a comprehensive framework for selecting outcomes in evaluating the clinical efficacy of Chinese herbal injections and to scientifically select outcomes for the clinical randomized controlled trial (RCT) of Tan-Re-Qing injection intervening AECOPD.

**Methods:**

A comprehensive literature review and consensus methods, including focus groups and Delphi surveys, were utilized.

**Results:**

Literature analysis identified 513 publications, encompassing regulatory guidance, guidelines, expert consensus, and RCTs. Initial dimensions include clinical efficacy, safety, and health economics. Primary outcomes should align with study objectives. Recommended evaluation domains include death, treatment outcome, future impact, quality of life, and safety. Commonly recommended outcomes comprise mortality, arterial blood gases, CAT, exacerbation frequency, adverse events, and lung function. Network meta-analysis identified specific therapeutic efficacy markers (white blood cell count, IL-6, IL-8). Quality of life assessment recommended SF-12, EQ-5D, or CAT. Emphasis on AECOPD frequency and lung function was noted. Delphi survey yielded 41 outcomes across various domains for evaluating Tan-Re-Qing in AECOPD.

**Conclusion:**

The findings contribute to developing a robust and reliable trial design for Tan-Re-Qing injection in AECOPD. The methodology employed in this study ensures a systematic and comprehensive approach to the selection of outcomes for the clinical evaluation of future studies in this field.

## Graphical Abstract

**Figure:**
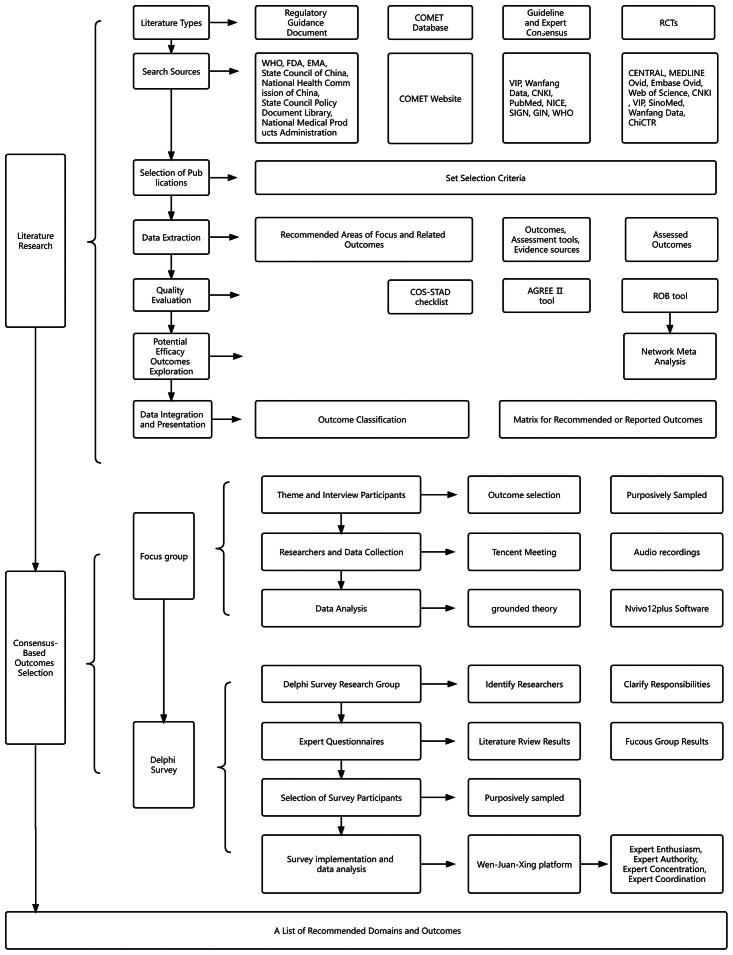

## Background

Chronic Obstructive Pulmonary Disease (COPD) is a heterogeneous lung condition characterized by chronic respiratory symptoms due to abnormalities of the airways and/or alveoli that cause persistent, often progressive, airflow obstruction. Acute Exacerbations of Chronic Obstructive Pulmonary Disease (AECOPD) is defined as an event characterized by dyspnea and/or cough and sputum that worsen over less than 14 days and is one of the most frequent complications in COPD patients, leading to increased mortality and healthcare costs [1, 2]. Chinese herbal injection (CHI), as an important component of traditional Chinese medicine, possesses a unique theoretical system and treatment methods that have shown enormous potential in the intervention of AECOPD [3, 4]. Studies have reported that Tan-Re-Qing injection, a Chinese herbal injection, has shown efficacy and safety for people with AECOPD [5–7]. Tan-Re-Qing injection, a brown-red clear liquid, is composed of five herbs, Huang-Qin (Scutellaria baicalensis Georgi, baical skullcap root, *Scutellariae Radix*), Xiongdanfen (Bear Gall Powder, *Fel Ursi*), Shanyangjiao (Saiga tatarica Linnaeus, Goat’s Horn, *cornu caprae hircus*), Jinyinhua (Lonicera japonica Thunt, Wild honeysuckle flower, *Lonicerae Japonicae Flos),* and Lianqiao (Forsythia suspensa (Thunb.) Vahl, Forsythiae Fructus, *Forsythia suspensa*) [8]. From the view of Chinese medicine, it has the effects of clearing heat, resolving phlegm, and detoxification. The chemical composition of Tan-Re-Qing injection includes amino acids, iridoid and secoirdoid components, isoflaudio-videoones, phenolic acids, phenylethanol glycosides, lignans, steroids, and others [9]. The main active ingredients of Tan-Re-Qing injection are generally recognized as baicalin and wild baicalin in Baical Skullcap Root, ursodeoxycholic acid and chenodeoxycholic acid in Bear Gall Powder, various amino acids in Goat’s Horn, chlorogenic acid, caffeic acid, and galuteolin in Wild Honeysuckle Flower, and forsythiaside in Forsythiae Fructus [10]. Clinically, it has antibacterial, antiviral, antipyretic, sedative, antitussive, and expectorant properties [11]. In clinical practice, Tan-Re-Qing injection is often used concurrently with corticosteroids, bronchodilators, and antibiotics to synergistically alleviate inflammation, relieve bronchospasm and swelling, reduce mucus secretion and expulsion, thereby further improving patients’ respiratory condition and expediting the recovery process from infection [12].

However, it remains a challenge to decide which outcomes to choose and how to select them to evaluate the clinical efficacy, safety, and cost-effectiveness of Chinese herbal injections, including Tan-Re-Qing injection, in the treatment of AECOPD. In clinical trials, the selection of outcomes plays a crucial role as they serve as key indicators for evaluating the effectiveness of therapeutic interventions [13, 14], reflecting disease status, and providing valuable insights into the clinical significance of interventions [15, 16]. Outcomes are not only useful for assessing effectiveness but also for evaluating the safety of therapeutic interventions. Monitoring adverse events, side effects, and other safety-related measures helps determine the impact of interventions on patient safety [17, 18]. Rational selection of outcomes provides robust scientific evidence, empowering doctors and patients to make informed treatment choices and guiding the development of clinical practice [14]. By employing uniform outcomes, researchers can analyze and evaluate results more effectively, facilitating comprehensive cross-study research [19]. Ethical considerations and adherence to regulatory requirements are essential to ensure that chosen outcomes meet scientific, ethical, and regulatory criteria [20, 21].

After the preliminary conducted systematic review of RCTs about the treatment of AECOPD with Tan-Re-Qing injection, we have identified several recurring issues in the outcomes within these RCTs. These issues are also commonly observed in clinical trials:

1. Lack of consistency: Different studies use different outcomes to assess treatment effects, making comparing and combining results difficult. The lack of consistency complicates the comprehensive evaluation of treatment effects and limits the reliability of existing research findings [22, 23].
2. Subjectivity and difficulty in quantification: Some disease states or treatment effects are difficult to measure objectively and quantitatively, relying only on subjective assessments from patients or doctors. This subjectivity can be influenced by individual differences, memory biases, or prejudices, leading to inconsistency and reduced reliability of results [24].
3. Insufficient sensitivity and specificity: Some outcomes may not accurately measure or capture changes in treatment effects. If an outcome measure is not sensitive or specific enough to treatment interventions, it may result in missing the true treatment effect or misinterpreting changes caused by other factors such as treatment effects [25].
4. Lack of patient-centered outcomes and misuse of surrogate outcomes: Many clinical studies lack focus and evaluation on patient-centered outcomes and instead prioritize the evaluation of surrogate outcomes (such as physiological or laboratory indicators) [26, 27]. Furthermore, the correlation and relevance between surrogate outcomes and ultimate clinical outcomes might be unclear. This introduces uncertainty when relying on these surrogate measures in clinical decision-making [28, 29].

Therefore, by researching the selection of outcomes in RCT of Tan-Re-Qing injection for AECOPD as a case study, we aim to explore methods for selecting outcomes in RCT evaluating the clinical efficacy of Chinese herbal injection in order to further establish a research framework and provide methodology reference. Additionally, we conducted this study to scientifically, systematically, and objectively select outcomes for the clinical randomized controlled trial (RCT) of Tan-Re-Qing injection intervening AECOPD.

## Research methodology

To investigate the efficacy of Tan-Re-Qing injection for AECOPD, our study adopted a comprehensive approach involving literature research, systematic review, and consensus-based outcome selection. Please refer to the Graphical Abstract for a detailed overview of the procedures.

### Literature research

We utilized a literature review to identify recommended domain and outcomes for clinical evaluation of AECOPD.

Firstly, we conducted an extensive search and review of regulatory guidance documents and core outcome set (COS) research sources such as the World Health Organization (WHO), China Basic Drug System Policy, China Basic Medical Insurance Medication Management Policy, National Medical Products Administration, China Good Clinical Practice (GCP, 2020), US Food and Drug Administration (FDA), European Medicines Agency (EMA) and Core outcomes in Effectiveness Trials (COMET) website to source recommended outcomes for AECOPD drug interventions. We extracted core areas of clinical concern and their corresponding outcomes.

Secondly, we thoroughly searched for globally published clinical guidelines and expert consensus on Western medicine, Traditional Chinese Medicine (TCM) and integrated TCM into the assessment and treatment recommendations for AECOPD. We identified and extracted outcomes recommended for the evaluation and treatment of AECOPD.

Thirdly, we performed a systematic review to identify globally published randomized controlled trials (RCTs) specifically focused on Chinese patent medicine injections for AECOPD. We extracted and analyzed the outcomes reported in these trials. We select core outcomes by considering recommendations from multiple literature sources and including measures reported in more than 6% of the RCTs.

Fourthly, we utilized network meta-analysis within the systematic review framework to analyze the core outcomes identified in the previous three steps. This network meta-analysis was conducted to explore the potential clinically efficacy outcomes of Chinese herbal injections for AECOPD. This systematic review protocol has been registered at Prospective Register of Systematic Reviews PROSPERO (https://www.crd.york.ac.uk/PROSPERO/, PROSPERO registration no. CRD42021286644). We have followed PRISMA guideline [30] for the reporting of our meta-analysis. The detailed results of our network meta-analysis can be found in another article, while this paper focuses on presenting partial efficacy outcomes on Tan-Re-Qing injection for AECOPD.

#### Search strategy

##### Regulatory guidance document

The task involved utilizing a computer to search for websites related to the regulatory bodies for clinical trials of pharmaceuticals both domestically and internationally, policies on basic drug systems, drug management policies under basic medical insurance, standards for quality management of drug clinical trials in China, and regulations on drug supervision and management in China. The searched websites include the World Health Organization (https://www.who.int/zh), the U.S. Food and Drug Administration (https://www.fda.gov/regulatory-information/search-fda-guidance-documents), the European Medicines Agency (https://www.ema.europa.eu/en/search/search/field_ema_web_topics%3Aname_field/Regulatory%20and%20procedural%20guidance?search_api_views_fulltext=), the official website of the State Council of the People’s Republic of China (https://www.gov.cn/), the official website of the National Health Commission of the People’s Republic of China (https://www.nhc.gov.cn), the State Council Policy Document Library (https://www.gov.cn/zhengce/zhengcewenjianku/), and the National Medical Products Administration (https://www.nmpa.gov.cn/index.html).

The search terms included ‘clinical trials’, ‘outcome’, ‘guideline’, ‘chronic obstructive pulmonary disease’, ‘medical insurance’, ‘drug clinical trials’, ‘technical guidance’, and ‘outcome indicators’. The search strategy combines the use of subject terms and free terms, and adjustments are made according to the specific requirements of different websites. The search was limited to documents published until January 1, 2023, and the language was restricted to Chinese and English.

##### COMET database

We also searched The Core Outcomes in Effectiveness Trials (COMET) website (https://comet-initiative.org/), a database that featured core outcome set studies and pooled comprehensive outcomes. We entered ‘Acute exacerbation of chronic obstructive pulmonary disease’ in the Disease name menu.

##### Guideline and expert consensus

Retrieve databases or websites, including VIP Chinese Science and Technology Journal Database (VIP), Wanfang Data, CNKI, PubMed, National Institute for Health and Care Excellence (NICE), Scottish Intercollegiate Guidelines Network (SIGN), Guidelines International Network (GIN), World Health Organization (WHO).

We used a combination of subject terms and free words for retrieval and made corresponding adjustments according to different databases. The search time limit is from January 1, 2012, to January 1, 2023, and the language is limited to Chinese and English. Chinese search terms included: 慢性阻塞性肺疾病急性加重 (AECOPD), 慢阻肺 (COPD), 慢性阻塞性肺疾病 (chronic obstructive pulmonary disease), 急性加重期慢性阻塞性肺疾病 (acute exacerbation of chronic obstructive pulmonary disease), 实践指南 (practice guideline), 指南 (guidelines), 共识 (consensus), 建议 (recommendation), 规范 (standards). English search terms include AECOPD, acute exacerbation of chronic obstructive pulmonary disease, COPD, chronic obstructive pulmonary disease, guideline, and practice guideline. See Table 1 for the guideline retrieval strategy.

**Table 1.** Guidelines and expert consensus search Strategy (using PubMed Database as an example).

Time span	Search strategy	
1974 to January 2023	#1	AECOPD [MeSH Terms]
	#2	COPD [Title/Abstract]
	#3	acute exacerbation of chronic obstructive pulmonary disease [Title/Abstract]
	#4	chronic obstructive pulmonary disease [Title/Abstract]
	#5	#1 OR #2 OR #3 OR #4
	#6	Practice Guideline[Publication Type]
	#7	Practice Guideline as Topic [MeSH Terms]
	#8	#6 OR #7
	#9	#5 AND #8

##### RCT retrieval of Chinese herbal injection for AECOPD

We searched Cochrane Central Register of Controlled Trials (CENTRAL), MEDLINE Ovid, Embase Ovid, Science Citation Index Expand (Web of Science), CNKI, VIP, SinoMed, and Wanfang Data. We also searched ongoing or unpublished studies in the Chinese Clinical Trial Registry (ChiCTR). See Table 2 for the RCT retrieval strategy.

**Table 2.** RCT search strategy for traditional Chinese medicine injections in AECOPD (using Embase Ovid Database as an example).

Time span	Search strategy	
1974 to July 2022	#1	Acute Exacerbation of Chronic Obstructive Pulmonary Disease [MeSH Terms]
	#2	Chronic obstructive airway disease OR chronic obstructive lung disease OR AECOPD OR COPD [Title/Abstract]
	#3	#1 OR #2
	#4	Chinese herbal medicine [MeSH Terms]
	#5	Chinese herbal medicine preparation OR Chinese herbal medicine injection OR Chinese herbal injection OR Traditional Chinese medicine OR Traditional Chinese drug OR Chinese herbal preparation OR Traditional Chinese preparation OR Chinese patent medicine OR CHMIs OR TCM [Title/Abstract]
	#6	Tanreqing injection OR Tan Re Qing injection OR Tan-Re-Qing injection OR Xuebijing injection OR Xue Bi Jing injection OR Xue-Bi-Jing injection OR Qingkailing injection OR Qing Kai Ling injection OR Qing-Kai-Ling injection OR Reduning injection OR Re Du ning injection OR Re-Du-Ning injection Chuankezhi injection OR Chuan Ke Zhi injection OR Chuan-Ke-Zhi injection OR Xixinnao injection OR Xi Xin Nao injection OR Xi-Xin-Nao injection OR Xiyanping injection OR Xi Yan Ping injection OR Xi-Yan-Ping injection OR Shenfu injection OR Shen Fu injection OR Shen-Fu injection OR Huangqi injection OR Huang Qi injection OR Huang-Qi injection OR Shengmai injection OR Sheng Mai injection OR Sheng-Mai injection OR Shenmai injection OR Shen Mai injection OR Shen-Mai injection OR Danshenchuanxiongqin injection OR Dan Shen Chuan Xiong Qin injection OR Dan-Shen-Chuan-Xiong-Qin injection OR Danhong injection OR Dan Hong injection OR Dan-Hong injection OR Xingnaojing injection OR Xing Nao Jing injection OR Xing-Nao-Jing injection OR Yinxing injection OR Yin Xing injection OR Yin-Xing injection OR Chuanxiongqinin jection OR Chuan Xiong Qin jection OR Chuan-Xiong-Qin jection OR Kushensu injection OR Ku Shen Su injection OR Ku-Shen-Su injection OR Dengzhanhuasu injection OR Deng Zhan Hua Su injection OR Deng-Zhan-Hua-Su injection OR Shuxuetong injection OR Shu Xue Tong injection OR Shu-Xue-Tong injection OR Shenqifuzheng injection OR Shen Qi Fu Zheng injection OR Shen-Qi-Fu-Zheng injection OR Yinxingdamo injection OR Yin Xing Da Mo injection OR Yin-Xing-Da-Mo injection OR Shuxuening injection OR Shu Xue Ning injection OR Shu-Xue-Ning injection OR Honghua injection OR Hong Hua injection OR Hong-Hua injection OR Zhichuanning injection OR Zhi Chuan Ning injection OR Zhi-Chuan-Ning injection OR Shuanghuanglian injection OR Shuang Huang Lian injection OR Shuang-Huang-Lian injection OR Hongjingtian injection OR Hong Jing Tian injection OR Hong-Jing-Tian OR Fufangdanggui injection OR Fu Fang Dang Gui injection OR Fu-Fang-Dang-Gui injection [ALL fields]
	#7	#4 OR #5 OR #6
	#8	Randomised OR Randomized OR Randomly OR Clinical trials OR Randomized controlled trial OR Randomised controlled trial OR Controlled clinical trial [Title/Abstract]
	#9	#3 AND #7 AND #8

#### Selection of publications

##### Selection of regulatory guidance documents

Inclusion criteria: Studies on the selection of outcomes or methods in AECOPD clinical trials (no limitation on study type); or Policies or regulations on the selection of outcomes in domestic and international clinical trials.

Exclusion criteria: Documents or studies for which relevant information cannot be obtained.

##### COMET publication selection

Inclusion criteria: Studies on COS and outcome measurement methods determined based on the consensus ranking method [31].

Exclusion criteria: Pure methodological studies, COS study protocols, outcome measure reviews, studies without consensus-based outcome measurement or symptom measurement.

##### Guideline and expert consensus selection

Comprehensively collect published guidelines and expert consensus on AECOPD/COPD from domestic and international sources. Supplemental screening is conducted for guidelines found in the literature. Only the latest updated version of guidelines published by the same institution was included.

##### RCT selection of Chinese herbal injection for AECOPD

Inclusion criteria:

1. Types of Studies
  All randomized clinical trials (RCTs).
2. Types of Participants
  Individuals diagnosed with AECOPD by physicians or guidelines are considered. The main symptoms of AECOPD are shortness of breath with wheezing, chest tightness, increased cough, sputum, fever, and changes in sputum color and/or consistency. Conditions with clinical and/or laboratory tests that could explain the sudden symptoms change should be excluded. Age, gender, ethnicity, region, disease stage, smoking history, and comorbidity are not excluded.
3. Types of Intervention
  Chinese Herbal Injections (CHIs) approved by the Chinese State Drug Administration, delivered at any dose, duration, follow-up time, and administration method, are considered. Conventional interventions (CIs) in the experimental and control groups are allowed, provided they are administered equally to all trial groups. The following comparisons were investigated: a. CHI versus no intervention; b. CHI plus CIs versus CIs; c. CHI versus placebo; d. CHI plus CIs versus placebo plus CIs; e. CHI versus another CHI; f. CHI plus CIs versus another CHI plus CIs; g. CHI versus CIs.

Exclusion criteria: Studies whose full text was not available were excluded. Republished or data-repetitive studies were retained for complete data.

The researchers independently used NoteExpress software (version 3.0) to read and screen literature. Inconsistencies were resolved through discussion and consultation with a third reviewer if necessary. We manually searched the reference list, retrieved included publications, and recorded reasons for exclusion.

### Data extraction

The researchers independently extracted data word by word and entered it into an electronic form, cross-checking to ensure accuracy. The recommended areas of focus and related outcomes were extracted for regulatory guidance documents and COS studies in the COMET database. Content, outcomes, assessment tools, and evidence sources were extracted for guidelines and expert consensus. Authors, year, sample size, control type, and assessed outcomes were extracted for RCTs. Outcome data for network meta-analyses were reported in another article. Only relevant important results are included in this paper.

### Quality evaluation

#### Quality evaluation of COMET publications

Independent evaluations are conducted based on the items listed in the COS-STAD checklist [31].

#### Quality evaluation of guidelines and consensus

The quality of retrieved guidelines and expert consensus was assessed using the AGREE II tool [32]. Statistical analysis is performed using SPSS 22.0, and the intra-class correlation coefficient (ICC) is used to test the consistency of the evaluation results [33]. If subsequent outcomes are only recommended by two Level C guidelines/consensuses, careful consideration should be given to the selection of those outcomes.

#### Quality evaluation of RCTs on traditional Chinese medicine injection intervention for AECOPD

The risk of bias in the included literature is assessed using the Cochrane Risk of Bias (ROB) tool [34]. RevMan v. 5.4.1 is utilized to illustrate the assessment of the risk of bias. Any conflicts that arise are resolved through discussion or consensus with HZ.

#### Data integration and presentation

##### Outcome classification

We extract outcomes from COS articles and regulatory guidance documents. We then categorized the outcomes into different domains based on their characteristics.

##### Matrix for quality assessment results

We constructed a matrix table to present the following information from guidelines and expert consensus, including quality assessment results, recommended evaluation domains, outcomes, and measurement tools.

##### Matrix for reported outcomes in RCTs

We created a matrix table to display the reported outcomes in randomized controlled trials (RCTs) related to Chinese herbal injections for AECOPD intervention.

##### Network meta-analysis (NMA) using Bayesian framework

We performed an NMA using a Bayesian framework. We utilize the node-splitting method to determine the appropriate model selection. If *p* ≥ 0.05, we used a consistency model. We conducted the Bayesian Markov chain Monte Carlo (MCMC) analysis using R software. We assessed the convergence by checking the Potential Scale Reduction Factor (PSRF), which should be between 1.00–1.05 to indicate satisfactory convergence. We set the Markov chain to 3 and performed 10,000 iterations. We performed 1,000 annealing operations using R software to eliminate the influence of initial values. We used relative risk (RR) to measure binary data and mean difference (MD) to measure continuous data.

## Consensus-based outcomes selection

We employed focus group interviews and a Delphi survey to select consensus-based outcomes for evaluating clinical RCT studies on Tan-Re-Qing injection for AECOPD.

### Focus group

#### Theme and interview participants

The theme of the study focuses on the selection of outcomes for RCT of Tan-Re-Qing injection intervening AECOPD clinical. We purposively sampled eight scholars with research experience in evaluating the clinical efficacy of traditional Chinese medicine.

#### Researchers and data collection

The data collection process for this study was conducted online using Tencent Meeting. Audio recordings were utilized to gather the necessary information. The research team consisted of Zhao Hui as the focus group moderator, Kong De Zhao as the preliminary literature research findings presenter, and Yang Si Hong as the interview recorder. Before the interview, the researchers introduced the study’s purpose, significance, process, and methodology to all participating group members. The interview session lasted for approximately two and a half hours. After transcription, the text was returned to the participants for their feedback.

#### Data analysis

Nvivo12plus software was used for coding the data. Data entry and analysis was based on grounded theory [35–37].

Open Coding: The first level of coding where various categories were assigned to the data after preliminary collection and analysis of the raw material. To avoid losing codes, we read the transcripts line by line.

Axial Coding: The second level of coding involves establishing organic connections between discovered categories and building conceptual relationships. We constantly compared codes with similar meanings and grouped them into the same category, aiming to consolidate the codes obtained through open coding. We followed the principle of keeping all valuable information.

Selective Coding: The third level of coding is to form core categories. After systematic analysis, we select and extract ‘core categories’ from all the identified categories. We then gathered scattered conceptual categories around the core categories, considering the research objectives and interview guidelines. Core codes served as a guide to branch out and extract a series of related content for further analysis in the next stage.

### Delphi survey

#### The establishment of the Delphi Survey research group

The Delphi Survey research group comprised two professionals engaged in evidence-based medicine, two clinical professionals with over ten years of experience in tertiary grade-A hospitals, and two researchers. The primary tasks of the group members included developing expert survey questionnaires, selecting survey participants, distributing and collecting the questionnaires, and organizing, summarizing, and analyzing the feedback and results.

#### Development of the expert questionnaires

The research team identified alternative outcomes through the former systematic review of international and domestic regulatory guidance documents on clinical drug evaluation, guidelines, expert consensus, and relevant RCTs on CHIs for AECOPD. A semi-open questionnaire was used, and the importance of each outcome was assessed according to the ranking method for outcomes described in the WHO Handbook for Guideline Development (2nd Edition) [38]. The questionnaire consisted of three parts. The first part collected basic information about the experts, including name, gender, affiliation, profession, years of experience, education, occupation, and title. The second part evaluated the importance of each outcome, allowing experts to assign scores from 1 to 9 (with 1–3 intervals indicating unimportant, 4–6 intervals indicating important but not critical, and 7–9 intervals indicating important and critical). Experts were also invited to provide suggestions and additional outcomes. The third part assessed the experts’ familiarity with the questionnaire and the basis for their judgments. Familiarity was rated on a five-point scale: very familiar (1 point), quite familiar (0.8 points), moderately familiar (0.6 points), not very familiar (0.4 points), and unfamiliar (0.2 points). The judgment basis was divided into four aspects: practical experience, theoretical analysis, relevant literature from domestic and international sources, and intuition, which were rated on a scale of low, medium, and high impact (with scores ranging from 0.1 to 0.3 for theoretical analysis, 0.3 to 0.5 for practical experience, 0.1 for all impact degree on relevant literature from domestic and international sources and tuition, where the highest point indicates high impact) [39, 40].

After the first round of the survey, the results and expert opinions were summarized and analyzed. A group meeting was held based on the statistical analysis results, and revisions were made to selecting outcomes for assessing Tan-Re-Qing injection for AECOPD. The questionnaire survey can be conducted for 2–3 rounds until a consensus is reached.

#### Selection of survey participants

We purposively sampled fifteen scholars. Selection criteria for scholars include a. A bachelor’s degree or higher. b. At least five years of experience in clinical medicine, evidence-based medicine, traditional Chinese medicine, or related fields. c. Demonstrated enthusiasm for the research and the ability to provide comprehensive opinions or suggestions from different perspectives. d. Willingness to participate in the expert questionnaire survey and commitment to cooperating fully.

#### Survey implementation and data analysis

The questionnaire was distributed online using the Wen-Juan-Xing platform (https://www.wjx.cn). The collected questionnaires were imported into Excel to establish a database. Statistical analysis using SPSS 26.0 software was conducted to assess the expert enthusiasm, credibility, and concentration, and coordination of their opinions. The mean, standard deviation, score frequency, and coefficient of variation were calculated.

##### Expert enthusiasm

The enthusiasm of experts is represented by the questionnaire response rate.

##### Expert authority

The degree of expert authority was represented by expert authority coefficient (Cr), calculated as Cr = (Ca + Cs)/2. Generally, the higher Cr is, the higher the prediction accuracy. A Cr value greater than 0.7 is considered to indicate acceptable reliability [41].

Ca represented judgement coefficient, considering the basis used by the experts when making a judgement, calculated as Ca = (0.3 × the frequency of high impact of theoretical analysis + 0.2 × the frequency of medium impact of theoretical analysis + 0.1 × the frequency of low impact of theoretical analysis)/15 + (0.5 × the frequency of high impact of practical experience + 0.4 × the frequency of medium impact of practical experience + 0.3 × the frequency of low impact of practical experience)/15+ (0.1 × the frequency of high impact of literature sources + 0.1 × the frequency of medium impact of literature sources + 0.1 × the frequency of low impact of literature sources)/15 + (0.1 × the frequency of high impact of tuition + 0.1 × the frequency of medium impact of tuition + 0.1 × the frequency of low impact of tuition)/15.

Cs represented familiarity coefficient, representing the degree of the expert’s familiarity with the problem, calculated as Cs = 1 × the frequency of very familiar + 0.8 × the frequency of quite familiar + 0.6 × the frequency of moderately familiar + 0.4 × the frequency of not very familiar + 0.2 × the frequency of unfamiliar [42, 43].

##### Degree of coordination and agreement of expert opinions

The degree of coordination of experts was presented by the coefficient of variation (CV). Coefficient of variation (CV) = standard deviation (SD)/mean. The mean is calculated by assigning a score of 1–9 to each item according to its importance and calculating the average. SD refers to the standard deviation of experts’ ratings of the importance of the item. A smaller CV indicates higher consistency in experts’ evaluation of the importance of the item. CV ≤ 30% is considered effective [44].

If the coefficient of expert agreement, represented by the percentage of votes (interval vote count/total number of votes) in 4–6 interval plus 7–9 any interval exceeded 80%, and the coefficient of variation was more than 0.3, the outcome is considered to be selected.

## Ethical approval statement

Institutional ethical approval for this study was obtained from the Ethics Committee of IRB of The Affiliated Hospital of Liaoning University of Traditional Chinese Medicine. The approval number is 2022117CS(KT)-091-01, issued on November 17^th^, 2022.

## Informed consent for participation statement

All experts provided informed consent prior to their participation in the study. Informed consent was obtained written from each participant, detailing the purpose of the study, procedures involved, potential risks, and their rights as participants.

## Results

### Literature research results

#### Characteristics of included publications

A total of 513 publications were retrieved, including four regulatory guidance documents, namely the WHO’s definition and requirements for Essential Medicines, China’s Essential Medicine System Policy, China’s Drug Supervision and Management Policies and Regulations, and EMA guideline, seven Guidelines and two Expert Consensus, one COS study [45], and 499 RCTs (see Table 3). Among the included publications, ten studies used the systematic review method, while another ten studies utilized the Delphi/NGT/other consensus group meeting method. Patient involvement was highlighted in one report, and healthcare professionals’ involvement was reported in 12 studies. For more detailed information about the categories and characteristics of the included publications, please refer to Table 4.

**Table 3.** Characteristics of included 497 RCTs.

ID(study, year)	Patients (no. I/C)	Type of intervention	Intervention	Control	Treatment duration(day)(D)
Cai 2010 [46]	40/40	CHI_CI versus CI	ChuanKZ_CI	CI	14D
Cai 2020 [47]	29/29	CHI_CI versus CI	XueBJ_CI	CI	7D
Cao 2012 [48]	42/40	CHI_CI versus CI	TanRQ_CI	CI	14D
Cha 2009 [49]	30/30	CHI_CI versus CI	TanRQ_CI	CI	14D
Chang 2007 [50]	29/29	CHI_CI versus CI	QingKL_CI	CI	14D
Chen 2007 [51]	40/36	CHI_CI versus CI	DanH_CI	CI	14D
Chen 2008 [52]	30/30	CHI_CI versus CI	DengZHS_CI	CI	7D
Chen 2009a [53]	48/48	CHI_CI versus CI	TanRQ_CI	CI	14D
Chen 2009b [54]	80/80	CHI_CI versus CI	TanRQ_CI	CI	14D
Chen 2011a [55]	43/43	CHI_CI versus CI	XueBJ_CI	CI	5D
Chen 2011b [56]	40/40	CHI_CI versus CI	TanRQ_CI	CI	10D
Chen 2011c [57]	28/32	CHI_CI versus CI	TanRQ_CI	CI	7D
Chen 2011d [58]	42/40	CHI_CI versus CI	YinXDM_CI	CI	14D
Chen 2011e [59]	30/30	CHI_CI versus CI	XueBJ_CI	CI	7D
Chen 2012a [60]	30/30	CHI_CI versus CI	ReDN_CI	CI	7–10D
Chen 2012b [61]	64/64	CHI_CI versus CI	TanRQ_CI	CI	N/A
Chen 2013 [62]	52/48	CHI_CI versus CI	DanH_CI	CI	14D
Chen 2014a [63]	77/77	CHI_CI versus CI	ReDN_CI	CI	7D
Chen 2014b [64]	30/30	CHI_CI versus CI	DanSDFSY_CI	CI	14D
Chen 2014c [65]	43/43	CHI_CI versus CI	ChuanXQ_CI	CI	10D
Chen 2016 [66]	64/64	CHI_CI versus CI	TanRQ_CI	CI	N/A
Chen 2017 [67]	42/40	CHI_CI versus CI	ShenQFZ_CI	CI	7D
Chen 2018 [68]	28/28	CHI_CI versus CI	XueBJ_CI	CI	14D
Chen 2019a [69]	45/45	CHI_CI versus CI	TanRQ_CI	CI	7D
Chen 2019b [70]	79/79	CHI_CI versus CI	TanRQ_CI	CI	14D
Chen 2020 [71]	37/37	CHI_CI versus CI	DanH_CI	CI	14D
Cheng 2011 [72]	60/30	CHI_CI versus CI	ChuanKZ_CI	CI	7D
Cheng 2013 [73]	45/45	CHI_CI versus CI	TanRQ_CI	CI	10D
Chi 2015 [74]	48/48	CHI_CI versus CI	ShenF_CI	CI	14D
Cui 2009 [75]	46/44	CHI_CI versus CI	TanRQ_CI	CI	12D
Cui 2010 [76]	24/24	CHI_CI versus CI	ChuanKZ_CI	CI	14D
Cui 2018 [77]	60/60	CHI_CI versus CI	TanRQ_CI	CI	N/A
Dai 2010 [78]	86/85	CHI_CI versus CI	TanRQ_CI	CI	8D
Dai 2018 [79]	86/88	CHI_CI versus CI	ShuXN_CI	CI	14D
Deng 2015 [80]	50/50	CHI_CI versus CI	DanS_CI	CI	28D
Ding 2007 [81]	36/32	CHI_CI versus CI	TanRQ_CI	CI	14–21D
Dong 2008 [82]	120/120	CHI_CI versus CI	XiXN_CI	CI	7–14D
Dong 2009 [83]	36/36	CHI_CI versus CI	TanRQ_CI	CI	10–14D
Dong 2016 [84]	32/30	CHI_CI versus CI	TanRQ_CI	CI	10D
Du 2009 [85]	50/50	CHI_CI versus CI	TanRQ_CI	CI	14D
Du 2014 [86]	30/30	CHI_CI versus CI	DengZHS_CI	CI	14D
Du 2015 [87]	60/60	CHI_CI versus CI	TanRQ_CI	CI	10D
Duan 2015 [88]	46/46	CHI_CI versus CI	XueBJ_CI	CI	7D
Fan 2012 [89]	86/86	CHI_CI versus CI	XueBJ_CI	CI	14D
Fang 2015 [90]	43/39	CHI_CI versus CI	TanRQ_CI	CI	14D
Fang 2019a [91]	58/58	CHI_CI versus CI	TanRQ_CI	CI	14D
Fang 2019b [92]	41/41	CHI_CI versus CI	TanRQ_CI	CI	7D
Fei 2021 [93]	30/30	CHI_CI versus CI	DanH_CI	CI	6D
Feng 2010 [94]	54/52	CHI_CI versus CI	TanRQ_CI	CI	14D
Feng 2013 [95]	40/40	CHI_CI versus CI	DanSCXQ_CI	CI	14D
Feng 2015 [96]	39/39	CHI_CI versus CI	TanRQ_CI	CI	14D
Feng 2018a [97]	69/68	CHI_CI versus CI	HuangQ_DanS_CI	CI	10D
Feng 2018b [98]	55/55	CHI_CI versus CI	DanSCXQ_CI	CI	10D
Feng 2019 [99]	20/20	CHI_CI versus CI	ShenF_CI	CI	14D
Feng 2020 [100]	33/33	CHI_CI versus CI	TanRQ_CI	CI	14D
Fu 2009a [101]	50/50	CHI_CI versus CI	TanRQ_CI	CI	10D
Fu 2009b [102]	65/63	CHI_CI versus CI	TanRQ_CI	CI	10D
Fu 2012 [103]	35/35	CHI_CI versus CI	TanRQ_CI	CI	15D
Fu 2014a [104]	30/27	CHI_CI versus CI	XueBJ_CI	CI	5D
Fu 2014b [105]	40/40	CHI_CI versus CI	XueBJ_CI	CI	10D
Fu 2016 [106]	30/30	CHI_CI versus CI	XueBJ_CI	CI	28D
Gao 2010a [107]	40/40	CHI_CI versus CI	XiXN_CI	CI	7D
Gao 2010b [108]	35/35	CHI_CI versus CI	ShenM_CI	CI	10D
Gao 2014 [109]	51/50	CHI_CI versus CI	XingNJ_CI	CI	10D
Gao 2021 [110]	31/31	CHI_CI versus CI	TanRQ_CI	CI	7D
Ge 2015 [111]	30/30	CHI_CI versus CI	TanRQ_CI	CI	10D
Geng 2018 [112]	42/41	CHI_CI versus CI	ShenF_CI	CI	10D
Gong 2009 [113]	44/43	CHI_CI versus CI	TanRQ_CI	CI	14D
Gu 2008 [114]	35/35	CHI_CI versus CI	TanRQ_CI	CI	15D
Guan 2008 [115]	16/16	CHI_CI versus CI	HuangQ_CI	CI	10D
Guan 2011 [116]	40/40	CHI_CI versus CI	DanH_CI	CI	14D
Guo 2009 [117]	35/35	CHI_CI versus CI	ShenM_CI	CI	15D
Guo 2010 [118]	33/30	CHI_CI versus CI	ShenM_CI	CI	14D
Guo 2018 [119]	36/36	CHI_CI versus CI	TanRQ_CI	CI	14D
Han 2010 [120]	20/20	CHI_CI versus CI	XueBJ_CI	CI	7D
Han 2012 [121]	23/27	CHI_CI versus CI	XiYP_CI	CI	14D
Han 2013 [122]	36/36	CHI_CI versus CI	ShenF_CI	CI	14D
Han 2015 [123]	62/60	CHI_CI versus CI	XiYP_CI	CI	14D
Hao 2010 [124]	30/30	CHI_CI versus CI	XueBJ_CI	CI	10D
Hao 2012 [125]	33/32	CHI_CI versus CI	TanRQ_CI	CI	10D
Hao 2017a [126]	75/75	CHI_CI versus CI	XiYP_CI	CI	10D
Hao 2017b [127]	75/75	CHI_CI versus CI	TanRQ_CI	CI	7D
Hao 2019a [128]	67/67	CHI_CI versus CI	KuSS_CI	CI	10D
Hao 2019b [129]	75/75	CHI_CI versus CI	XingNJ_CI	CI	9D
He 2008 [130]	45/45	CHI_CI versus CI	QingKL_CI	CI	7D
He 2010 [131]	20/20	CHI_CI versus CI	DanSDFSY_CI	CI	14D
He 2012 [132]	40/42	CHI_CI versus CI	DanS_CI	CI	10–14D
He 2014 [133]	50/50	CHI_CI versus CI	TanRQ_CI	CI	N/A
He 2015 [134]	30/30	CHI_CI versus CI	ShuXN_CI	CI	14D
He 2016 [135]	35/35	CHI_CI versus CI	TanRQ_CI	CI	7D
He 2019 [136]	44/44	CHI_CI versus CI	XueBJ_CI	CI	14D
Hong 2008 [137]	22/21	CHI_CI versus CI	TanRQ_CI	CI	10D
Hou 2016 [138]	30/30	CHI_CI versus CI	XiYP_CI	CI	10D
Hu 2014 [139]	30/30	CHI_CI versus CI	XiYP_CI	CI	10–14D
Hu 2016a [140]	45/45	CHI_CI versus CI	TanRQ_CI	CI	10D
Hu 2016b [141]	50/50	CHI_CI versus CI	XiYP_CI	CI	5D
Hu 2016c [142]	50/50	CHI_CI versus CI	TanRQ_CI	CI	7D
Hu 2018 [143]	43/43	CHI_CI versus CI	ChuanKZ_CI	CI	14D
Hu 2019 [144]	60/60	CHI_CI versus CI	TanRQ_CI	CI	10D
Hu 2020 [145]	60/60	CHI_CI versus CI	TanRQ_CI	CI	10D
Huang 2004 [146]	26/15	CHI_CI versus CI	ChuanXQ_CI	CI	14D
Huang 2006 [6]	30/30	CHI_CI versus CI	TanRQ_C	CI	14d
Huang 2009 [147]	50/48	CHI_CI versus CI	TanRQ_CI	CI	10D
Huang 2012 [148]	60/60	CHI_CI versus CI	TanRQ_CI	CI	7–14D
Huang 2013 [149]	45/43	CHI_CI versus CI	ChuanKZ_CI	CI	7D
Huang 2014a [150]	30/30	CHI_CI versus CI	DanSDFSY_CI	CI	14D
Huang 2014b [151]	50/50	CHI_CI versus CI	ShenM_CI	CI	7D
Huang 2017a [152]	16/16	CHI_CI versus CI	XueBJ_CI	CI	7D
Huang 2017b [153]	30/30	CHI_CI versus CI	ReDN_CI	CI	7D
Ji 2013 [154]	44/44	CHI_CI versus PLACEBO_CI	XueBJ_CI	PLACEBO_CI	10D
Jia 2018 [155]	40/40	CHI_CI versus CI	TanRQ_CI	CI	7–10D
Jiang 2014 [156]	33/30	CHI_CI versus CI	XueBJ_CI	CI	14D
Jiang 2015 [157]	18/18	CHI_CI versus CI	ShenQFZ_CI	CI	N/A
Jiang 2017 [158]	36/36	CHI_CI versus CHI_CI	DanS_CI	DengZHS_CI	10D
Jiang 2020 [159]	30/30	CHI_CI versus CI	TanRQ_CI	CI	15D
Jin 2018 [160]	34/36	CHI_CI versus CI	ShenF_CI	CI	14D
Jing 2015 [161]	35/35	CHI_CI versus CI	TanRQ_CI	CI	N/A
Ju 2012 [162]	30/30	CHI_CI versus CI	TanRQ_CI	CI	14D
Lai 2013 [163]	28/28	CHI_CI versus CI	TanRQ_CI	CI	10D
Lei 2016 [164]	30/30	CHI_CI versus CI	TanRQ_CI	CI	7D
Li 2007 [165]	28/24	CHI_CI versus CI	TanRQ_CI	CI	10D
Li 2008a [166]	30/28	CHI_CI versus CI	XingNJ_CI	CI	7D
Li 2008b [167]	34/34	CHI_CI versus CI	TanRQ_CI	CI	7D
Li 2008c [168]	30/30	CHI_CI versus CI	TanRQ_CI	CI	14D
Li 2008d [169]	28/24	CHI_CI versus CI	TanRQ_CI	CI	10D
Li 2008e [170]	40/40	CHI_CI versus CI	TanRQ_CI	CI	10D
Li 2008f [171]	54/36	CHI_CI versus CI	TanRQ_CI	CI	7–10D
Li 2009a [172]	36/36	CHI_CI versus CI	TanRQ_CI	CI	N/A
Li 2009b [173]	48/48	CHI_CI versus CI	TanRQ_CI	CI	14D
Li 2010a [174]	28/27	CHI_CI versus CI	ShenM_CI	CI	14D
Li 2010b [175]	28/27	CHI_CI versus CI	ShenM_CI	CI	14D
Li 2010c [176]	48/48	CHI_CI versus CI	ChuanXQ_CI	CI	10–14D
Li 2010d [177]	24/24	CHI_CI versus CI	TanRQ_CI	CI	14D
Li 2011 [178]	30/30	CHI_CI versus CI	ShenM_CI	CI	14D
Li 2011a [179]	28/28	CHI_CI versus CI	TanRQ_CI	CI	7D
Li 2012b [180]	40/40	CHI_CI versus CI	TanRQ_CI	CI	12D
Li 2012c [181]	60/60	CHI_CI versus CI	XueBJ_CI	CI	10D
Li 2013 [182]	40/40	CHI_CI versus CI	TanRQ_CI	CI	7D
Li 2014a [183]	60/60	CHI_CI versus CI	ChuanKZ_CI	CI	7D
Li 2014b [184]	38/38	CHI_CI versus CI	TanRQ_CI	CI	7D
Li 2014c [185]	45/45	CHI_CI versus CI	TanRQ_CI	CI	14D
Li 2015a [186]	50/50	CHI_CI versus CI	XueBJ_CI	CI	N/A
Li 2015b [187]	33/33	CHI_CI versus CI	XueBJ_CI	CI	N/A
Li 2015c [188]	40/40	CHI_CI versus CI	TanRQ_CI	CI	N/A
Li 2015d [189]	44/44	CHI_CI versus CI	DengZHS_CI	CI	10D
Li 2015e [190]	56/52	CHI_CI versus CI	TanRQ_CI	CI	10–14D
Li 2015f [191]	50/50	CHI_CI versus CI	XueBJ_CI	CI	7D
Li 2016a [192]	64/59	CHI_CI versus CI	TanRQ_CI	CI	10–14D
Li 2016b [193]	40/40	CHI_CI versus CI	DanSDFSY_CI	CI	7D
Li 2016c [194]	20/20	CHI_CI versus CI	DanSDFSY_CI	CI	6D
Li 2016d [195]	65/65	CHI_CI versus CI	DanSDFSY_CI	CI	14D
Li 2016e [196]	41/41	CHI_CI versus CI	XueBJ_CI	CI	14D
Li 2016f [197]	50/50	CHI_CI versus CI	XueBJ_CI	CI	15D
Li 2017 [198]	30/30	CHI_CI versus CI	DanS_CI	CI	14D
Li 2018a [199]	38/38	CHI_CI versus CI	XueBJ_CI	CI	10D
Li 2018b [200]	40/40	CHI_CI versus CI	XiXN_CI	CI	10D
Li 2018c [201]	45/45	CHI_CI versus CI	TanRQ_CI	CI	10D
Li 2019a [202]	45/45	CHI_CI versus CI	ChuanKZ_CI	CI	14D
Li 2019b [203]	40/40	CHI_CI versus CI	ChuanKZ_CI	CI	7D
Li 2019c [204]	42/42	CHI_CI versus CI	TanRQ_CI	CI	14D
Li 2019d [205]	42/42	CHI_CI versus CI	ShenQFZ_CI	CI	7D
Li 2019e [206]	70/68	CHI_CI versus CI	TanRQ_CI	CI	10D
Li 2020a [207]	25/25	CHI_CI versus CI	ChuanKZ_CI	CI	15D
Li 2020b [208]	41/41	CHI_CI versus CI	XueBJ_CI	CI	8D
Li 2021a [209]	50/50	CHI_CI versus CI	TanRQ_CI	CI	14D
Li 2021b [210]	72/72	CHI_CI versus CI	TanRQ_CI	CI	10D
Li 2021c [211]	69/68	CHI_CI versus CI	ChuanKZ_CI	CI	14D
Lian 2007 [212]	30/30	CHI_CI versus CI	TanRQ_CI	CI	10D
Lian 2018 [213]	51/51	CHI_CI versus CI	ShuXN_CI	CI	14D
Lian 2019 [214]	100/100	CHI_CI versus CI	XueBJ_CI	CI	5D
Liang 2009a [215]	30/30	CHI_CI versus CI	DanS_CI	CI	14D
Liang 2009b [216]	30/26	CHI_CI versus CI	TanRQ_CI	CI	15D
Liang 2012 [217]	15/15	CHI_CI versus CI	XiYP_CI	CI	14D
Liang 2016 [218]	40/40	CHI_CI versus CI	TanRQ_CI	CI	7D
Liao 2008 [219]	30/28	CHI_CI versus CI	ShenF_CI	CI	14D
Liao 2015 [220]	35/35	CHI_CI versus CI	QingKL_CI	CI	14D
Lin 2005 [221]	88/80	CHI_CI versus CI	TanRQ_CI	CI	7–10D
Lin 2009 [222]	30/30	CHI_CI versus CI	XueBJ_CI	CI	7D
Lin 2014 [223]	46/46	CHI_CI versus CI	DanSDFSY_CI	CI	10D
Liu 2007 [224]	100/98	CHI_CI versus CI	TanRQ_CI	CI	7D
Liu 2008a [225]	60/60	CHI_CI versus CI	TanRQ_CI	CI	14D
Liu 2008b [226]	61/61	CHI_CI versus CI	TanRQ_CI	CI	7D
Liu 2009 [227]	44/42	CHI_CI versus CI	TanRQ_CI	CI	N/A
Liu 2010a [228]	46/46	CHI_CI versus CI	ShenM_CI	CI	14D
Liu 2010b [229]	30/30	CHI_CI versus CI	XiXN_CI	CI	7–10D
Liu 2010c [230]	40/40	CHI_CI versus CI	ChuanKZ_CI	CI	15D
Liu 2010d [231]	50/46	CHI_CI versus CI	DanH_CI	CI	14D
Liu 2011a [232]	58/50	CHI_CI versus CI	TanRQ_CI	CI	21D
Liu 2011b [233]	30/30	CHI_CI versus CI	YinX_CI	CI	14D
Liu 2011c [234]	50/46	CHI_CI versus CI	YinX_CI	CI	14D
Liu 2011d [235]	60/60	CHI_CI versus CI	TanRQ_CI	CI	10–14D
Liu 2012a [236]	31/31	CHI_CI versus CI	TanRQ_CI	CI	14D
Liu 2012b [237]	100/100	CHI_CI versus CI	XiXN_CI	CI	N/A
Liu 2013a [238]	31/27	CHI_CI versus CI	ShenM_CI	CI	14D
Liu 2013b [239]	40/40	CHI_CI versus CI	DanH_CI	CI	10D
Liu 2014a [240]	50/50	CHI_CI versus CI	TanRQ_CI	CI	7D
Liu 2014b [241]	60/60	CHI_CI versus CI	ChuanXQ_CI	CI	10D
Liu 2014c [242]	56/54	CHI_CI versus CI	TanRQ_CI	CI	10D
Liu 2015a [243]	36/36	CHI_CI versus CI	DanSCXQ_CI	CI	15D
Liu 2015b [244]	25/25	CHI_CI versus CI	ChuanKZ_CI	CI	7–10D
Liu 2016 [245]	30/30	CHI_CI versus CI	XiYP_CI	CI	N/A
Liu 2017a [246]	45/45	CHI_CI versus CI	DanSCXQ_CI	CI	10D
Liu 2017b [247]	45/45	CHI_CI versus CI	DanSCXQ_XiYP_CI	CI	10D
Liu 2017c [248]	51/51	CHI_CI versus CI	XueBJ_CI	CI	N/A
Liu 2017d [249]	61/61	CHI_CI versus CI	ChuanKZ_CI	CI	5–14D
Liu 2017e [250]	58/58	CHI_CI versus CI	TanRQ_CI	CI	14D
Liu 2017f [251]	39/39	CHI_CI versus CI	TanRQ_CI	CI	42D
Liu 2018a [252]	30/30	CHI_CI versus CI	TanRQ_CI	CI	10D
Liu 2018b [253]	6/6	CHI_CI versus CI	TanRQ_CI	CI	14D
Liu 2019a [254]	39/39	CHI_CI versus CI	XiXN_CI	CI	14D
Liu 2019b [255]	35/35	CHI_CI versus CI	ShuXT_CI	CI	14D
Liu 2019c [256]	30/30	CHI_CI versus CI	DanH_CI	CI	14D
Liu 2019d [257]	30/30	CHI_CI versus CI	ShuXT_CI	CI	14D
Liu 2020a [258]	61/61	CHI_CI versus CI	ChuanKZ_CI	CI	14D
Liu 2020b [259]	40/40	CHI_CI versus CI	DanH_CI	CI	14D
Liu 2021 [260]	34/34	CHI_CI versus CI	TanRQ_CI	CI	14D
Long 2012 [261]	64/60	CHI_CI versus CI	TanRQ_CI	CI	7D
Long 2013 [262]	89/89	CHI_CI versus CI	XiXN_CI	CI	7D
Lou 2011a [263]	30/30	CHI_CI versus CI	ShuXN_CI	CI	14D
Lou 2011b [264]	30/30	CHI_CI versus CI	ShuXN_CI	CI	14D
Lou 2011c [265]	30/30	CHI_CI versus CI	ShuXN_CI	CI	14D
Lu 2010 [266]	28/30	CHI_CI versus CI	ChuanXQ_CI	CI	10D
Lu 2013a [267]	30/30	CHI_CI versus CI	TanRQ_CI	CI	10D
Lu 2013b [268]	49/49	CHI_CI versus CI	TanRQ_CI	CI	14D
Lu 2014 [269]	35/35	CHI_CI versus CI	TanRQ_CI	CI	14D
Lu 2015 [270]	48/48	CHI_CI versus CI	XiXN_CI	CI	14D
Lu 2018 [271]	54/54	CHI_CI versus CI	QingKL_CI	CI	15D
Luo 2009 [272]	35/32	CHI_CI versus CI	TanRQ_CI	CI	14D
Luo 2015 [273]	20/20	CHI_CI versus CI	XueBJ_CI	CI	5D
Luo 2017 [274]	24/24	CHI_CI versus CI	XueBJ_CI	CI	14D
Luo 2019 [275]	41/40	CHI_CI versus CI	TanRQ_CI	CI	10D
Lv 2015 [276]	36/36	CHI_CI versus CI	ShengM_CI	CI	7D
Ma 2009a [277]	30/30	CHI_CI versus CI	TanRQ_CI	CI	14D
Ma 2009b [278]	60/52	CHI_CI versus CI	TanRQ_CI	CI	14–21D
Ma 2011 [279]	30/30	CHI_CI versus CI	XueBJ_CI	CI	10D
Ma 2013 [280]	60/60	CHI_CI versus CI	TanRQ_CI	CI	10D
Ma 2015 [281]	41/39	CHI_CI versus CI	ChuanXQ_CI	CI	15D
Ma 2020a [282]	44/40	CHI_CI versus CI	DanH_CI	CI	14D
Ma 2020b [283]	43/43	CHI_CI versus CI	ReDN_CI	CI	10D
Ma 2021 [284]	63/63	CHI_CI versus CI	TanRQ_CI	CI	N/A
Mao 2007 [285]	79/88	CHI_CI versus CI	TanRQ_CI	CI	10D
Mao 2021 [286]	50/50	CHI_CI versus CI	XingNJ_CI	CI	N/A
Meng 2018 [287]	40/41	CHI_CI versus CI	DanSCXQ_CI	CI	14D
Min 2014 [288]	43/41	CHI_CI versus CI	ShenF_CI	CI	14D
Ni 2012 [289]	32/28	CHI_CI versus CI	XueBJ_CI	CI	7D
Nie 2013 [290]	50/50	CHI_CI versus CI	ReDN_CI	CI	3D
Ou 2007 [291]	30/30	CHI_CI versus CI	HuangQ_CI	CI	7D
Pa 2014 [292]	57/40	CHI_CI versus CI	TanRQ_CI	CI	10D
Pan 2009 [293]	60/60	CHI_CI versus CI	TanRQ_CI	CI	14D
Pan 2015 [294]	71/71	CHI_CI versus CI	TanRQ_CI	CI	15D
Pang 2015 [295]	55/55	CHI_CI versus CI	ReDN_CI	CI	14D
Peng 2007 [296]	30/30	CHI_CI versus CI	TanRQ_CI	CI	12D
Peng 2008a [297]	44/43	CHI_CI versus CI	TanRQ_CI	CI	12D
Peng 2008b [298]	32/30	CHI_CI versus CI	XueBJ_CI	CI	3D
Peng 2012a [299]	40/40	CHI_CI versus CI	YinX_CI	CI	14D
Peng 2012b [300]	26/26	CHI_CI versus CI	TanRQ_CI	CI	14D
Peng 2016 [301]	60/60	CHI_CI versus CI	ChuanKZ_CI	CI	7D
Peng 2021 [302]	36/36	CHI_CI versus CI	ReDN_CI	CI	10D
Qi 2008 [303]	40/40	CHI_CI versus CI	QingKL_CI	CI	7D
Qian 2008 [304]	30/30	CHI_CI versus CI	TanRQ_CI	CI	7D
Qian 2013 [305]	30/30	CHI_CI versus CI	XiYP_CI	CI	10D
Qian 2015 [306]	32/32	CHI_CI versus CI	ChuanKZ_CI	CI	14D
Qian 2020 [307]	24/24	CHI_CI versus CI	TanRQ_CI	CI	7D
Qian 2021 [308]	60/60	CHI_CI versus CI	ChuanKZ_CI	CI	14D
Qiao 2013 [309]	46/46	CHI_CI versus CI	TanRQ_CI	CI	7D
Qin 2010 [310]	35/35	CHI_CI versus CI	ShenF_CI	CI	7D
Qiu 2013a [311]	74/73	CHI_CI versus CI	HuangQ_CI	CI	N/A
Qiu 2013b [312]	35/35	CHI_CI versus CI	XueBJ_CI	CI	10D
Qiu 2013c [313]	31/27	CHI_CI versus CI	XueBJ_CI	CI	7D
Qu 2014 [314]	30/30	CHI_CI versus CI	HongJT_CI	CI	10D
Quan 2019 [315]	50/50	CHI_CI versus CI	TanRQ_CI	CI	7D
Rao 2012 [316]	50/50	CHI_CI versus CI	ReDN_CI	CI	7D
Ren 2013 [317]	35/35	CHI_CI versus CI	ShenF_CI	CI	14D
Ren 2021 [318]	90/90	CHI_CI versus CI	TanRQ_CI	CI	7D
Ruan 2017 [319]	64/64	CHI_CI versus CI	ShenM_CI	CI	14D
Shan 2008 [320]	20/20	CHI_CI versus CI	XueBJ_CI	CI	7D
Shao 2013 [321]	30/30	CHI_CI versus CI	XueBJ_CI	CI	9D
Shao 2014 [322]	50/50	CHI_CI versus CI	ShuXT_CI	CI	10D
Shao 2019 [323]	34/34	CHI_CI versus CI	TanRQ_CI	CI	N/A
Shen 2017a [324]	56/56	CHI_CI versus CI	DanH_CI	CI	14D
Shen 2017b [325]	40/40	CHI_CI versus CI	ShuXT_CI	CI	14D
Shen 2017c [326]	65/65	CHI_CI versus CI	ShenM_CI	CI	14D
Shi 2009 [327]	23/23	CHI_CI versus CI	DanH_CI	CI	14D
Shi 2012 [328]	45/41	CHI_CI versus CI	TanRQ_CI	CI	10D
Shi 2013 [329]	42/42	CHI_CI versus CI	TanRQ_CI	CI	10D
Shi 2016 [330]	40/40	CHI_CI versus CI	ShuXN_CI	CI	14D
Shi 2020 [331]	42/42	CHI_CI versus CI	XingNJ_CI	CI	5D
Shi 2021 [332]	42/42	CHI_CI versus CI	TanRQ_CI	CI	14D
Shu 2009 [333]	60/60	CHI_CI versus CI	ShuangHL_CI	CI	14D
Su 2017 [334]	32/32	CHI_CI versus CI	ShuXT_CI	CI	10D
Su 2019 [335]	30/30	CHI_CI versus CI	ChuanKZ_CI	CI	7D
Sun 2008 [336]	115/115	CHI_CI versus CI	XiXN_CI	CI	10D
Sun 2012a [337]	52/50	CHI_CI versus CI	ReDN_CI	CI	7–10D
Sun 2012b [338]	28/28	CHI_CI versus CI	TanRQ_CI	CI	7D
Sun 2013 [339]	43/43	CHI_CI versus CI	ChuanKZ_CI	CI	14D
Sun 2014 [340]	60/55	CHI_CI versus CI	FuFDG_CI	CI	7D
Sun 2018a [341]	44/44	CHI_CI versus CI	YinXDM_CI	CI	14D
Sun 2018b [342]	58/58	CHI_CI versus CI	TanRQ_CI	CI	14D
Tan 2017 [343]	90/90	CHI_CI versus CI	TanRQ_CI	CI	30D
Tang 2008 [344]	40/40	CHI_CI versus CI	DanSCXQ_CI	CI	14D
Tang 2012 [103]	26/24	CHI_CI versus CI	TanRQ_CI	CI	15D
Tang 2013 [345]	30/30	CHI_CI versus CI	XueBJ_CI	CI	7D
Tang 2014 [346]	23/23	CHI_CI versus CI	TanRQ_CI	CI	14D
Tang 2018 [347]	44/42	CHI_CI versus CI	ShenF_CI	CI	7D
Tian 2015 [348]	37/37	CHI_CI versus CI	ReDN_CI	CI	10D
Tong 2012 [349]	40/38	CHI_CI versus CI	YinXDM_CI	CI	14D
Tong 2018 [350]	60/60	CHI_CI versus CI	ShenF_KuDZ_CI	CI	14D
Tu 2010 [351]	24/24	CHI_CI versus CI	XueBJ_CI	CI	7D
Wan 2013 [352]	38/38	CHI_CI versus CI	ChuanXQ_CI	CI	14D
Wang 2005 [353]	50/50	CHI_CI versus CI	TanRQ_CI	CI	7D
Wang 2006 [354]	34/33	CHI_CI versus CI	TanRQ_CI	CI	12D
Wang 2007a [355]	46/40	CHI_CI versus CI	TanRQ_CI	CI	7–10D
Wang 2007b [356]	65/61	CHI_CI versus CI	XueBJ_CI	CI	10D
Wang 2007c [357]	32/28	CHI_CI versus CI	ShengM_CI	CI	14D
Wang 2008 [358]	50/46	CHI_CI versus CI	TanRQ_CI	CI	7–14D
Wang 2009a [359]	60/60	CHI_CI versus CI	ShenF_CI	CI	10D
Wang 2009b [360]	43/43	CHI_CI versus CI	XueBJ_CI	CI	14D
Wang 2011a [361]	72/70	CHI_CI versus CI	TanRQ_CI	CI	10–14D
Wang 2011b [362]	28/28	CHI_CI versus CI	YinXDM_CI	CI	15D
Wang 2011c [363]	39/39	CHI_CI versus CI	TanRQ_CI	CI	14D
Wang 2012a [364]	39/40	CHI_CI versus CI	ShuXN_CI	CI	14D
Wang 2012b [365]	50/50	CHI_CI versus CI	XueBJ_CI	CI	7D
Wang 2012c [366]	40/40	CHI_CI versus CI	DanH_CI	CI	14D
Wang 2012d [367]	30/30	CHI_CI versus CI	TanRQ_CI	CI	7D
Wang 2013a [368]	30/30	CHI_CI versus CI	ChuanXQ_CI	CI	14D
Wang 2013b [369]	47/47	CHI_CI versus CI	TanRQ_CI	CI	10D
Wang 2013c [370]	43/42	CHI_CI versus CI	DanH_CI	CI	15D
Wang 2013d [371]	30/30	CHI_CI versus CI	ShenF_CI	CI	N/A
Wang 2014a [372]	37/37	CHI_CI versus CI	ChuanKZ_CI	CI	15D
Wang 2014b [373]	26/26	CHI_CI versus CI	ShuXT_CI	CI	10D
Wang 2015a [374]	43/42	CHI_CI versus CI	ChuanKZ_CI	CI	84D
Wang 2015b [375]	46/46	CHI_CI versus CI	TanRQ_CI	CI	7D
Wang 2015c [376]	30/30	CHI_CI versus CI	XueBJ_CI	CI	8D
Wang 2015d [377]	50/50	CHI_CI versus CI	DanS_CI	CI	15D
Wang 2016a [378]	31/31	CHI_CI versus CI	XueBJ_CI	CI	7D
Wang 2016b [379]	25/25	CHI_CI versus CI	ChuanKZ_CI	CI	7D
Wang 2017a [380]	60/60	CHI_CI versus CI	TanRQ_CI	CI	10D
Wang 2017b [381]	80/80	CHI_CI versus CI	ChuanKZ_CI	CI	14D
Wang 2018 [382]	45/45	CHI_CI versus CI	ChuanXQ_CI	CI	14D
Wang 2019a [383]	60/60	CHI_CI versus CI	DanH_CI	CI	14D
Wang 2019b [384]	43/43	CHI_CI versus CI	TanRQ_CI	CI	14D
Wang 2019c [385]	59/59	CHI_CI versus CI	TanRQ_CI	CI	7D
Wei 2011 [386]	40/40	CHI_CI versus CI	TanRQ_CI	CI	14D
Wei 2014a [387]	37/33	CHI_CI versus CI	ReDN_CI	CI	10D
Wei 2014b [388]	32/32	CHI_CI versus CI	YinX_CI	CI	14D
Wei 2017 [389]	60/60	CHI_CI versus CI	ChuanXQ_CI	CI	14D
Wen 2018 [390]	44/44	CHI_CI versus CI	ChuanKZ_CI	CI	15D
Wu 2008 [391]	30/30	CHI_CI versus CI	ChuanXQ_CI	CI	14D
Wu 2009a [392]	40/40	CHI_CI versus CI	TanRQ_CI	CI	7D
Wu 2009b [393]	35/33	CHI_CI versus CI	TanRQ_CI	CI	14D
Wu 2010a [394]	51/54	CHI_CI versus CI	TanRQ_CI	CI	7D
Wu 2010b [395]	40/40	CHI_CI versus CI	TanRQ_CI	CI	7D
Wu 2011 [396]	40/40	CHI_CI versus CI	XiXN_CI	CI	15D
Wu 2013a [397]	25/25	CHI_CI versus CI	ChuanKZ_CI	CI	7D
Wu 2013b [398]	98/90	CHI_CI versus CI	ChuanXQ_CI	CI	12D
Wu 2015a [399]	50/50	CHI_CI versus CI	TanRQ_CI	CI	15D
Wu 2015b [400]	38/38	CHI_CI versus CI	DanSDFSY_CI	CI	14D
Wu 2016 [401]	56/64	CHI_CI versus CI	TanRQ_CI	CI	10D
Wu 2017 [402]	40/40	CHI_CI versus CI	ChuanKZ_CI	CI	7D
Wu 2018 [403]	41/41	CHI_CI versus CI	TanRQ_CI	CI	7D
Wu 2019 [404]	41/41	CHI_CI versus CI	TanRQ_CI	CI	14D
Wu 2020 [405]	60/60	CHI_CI versus CI	TanRQ_CI	CI	14D
Xi 2016 [406]	41/40	CHI_CI versus CI	TanRQ_CI	CI	7–14D
Xi 2017 [407]	39/39	CHI_CI versus CI	XueBJ_CI	CI	10D
Xia 2014a [408]	56/54	CHI_CI versus CI	ShenM_CI	CI	12D
Xia 2014b [409]	46/46	CHI_CI versus CI	DanSCXQ_CI	CI	12D
Xiang 2013 [410]	50/50	CHI_CI versus CI	ShenQFZ_CI	CI	7D
Xiang 2019 [411]	60/60	CHI_CI versus CI	XiXN_CI	CI	5D
Xiang 2020 [412]	60/60	CHI_CI versus CI	TanRQ_CI	CI	10D
Xiao 2007 [413]	32/32	CHI_CI versus CI	ShenM_CI	CI	14D
Xiao 2018 [414]	46/46	CHI_CI versus CI	HuangQ_CI	CI	14D
Xie 2005 [415]	92/30	CHI_CI versus CI	XiXN_CI	CI	15D
Xie 2019 [416]	35/35	CHI_CI versus CI	TanRQ_CI	CI	10–15D
Xie 2020 [417]	40/40	CHI_CI versus CI	TanRQ_CI	CI	14D
Xiong 2013 [418]	56/56	CHI_CI versus CI	HuangQ_CI	CI	14D
Xiong 2021 [419]	34/34	CHI_CI versus CI	TanRQ_CI	CI	7D
Xu 2006 [420]	27/25	CHI_CI versus CI	TanRQ_CI	CI	12D
Xu 2007 [421]	37/30	CHI_CI versus CI	ShenF_CI	CI	10D
Xu 2008 [422]	25/25	CHI_CI versus CI	ChuanKZ_CI	CI	7D
Xu 2010a [423]	106/102	CHI_CI versus CI	XiXN_CI	CI	14D
Xu 2010b [424]	15/15	CHI_CI versus CI	ReDN_CI	CI	7D
Xu 2010c [425]	35/25	CHI_CI versus CI	TanRQ_CI	CI	14D
Xu 2010d [426]	30/30	CHI_CI versus CI	TanRQ_CI	CI	10D
Xu 2011a [427]	28/28	CHI_CI versus CI	XingNJ_CI	CI	14D
Xu 2011b [428]	15/15	CHI_CI versus CI	ReDN_CI	CI	7D
Xu 2014 [429]	30/30	CHI_CI versus CI	DanST_CI	CI	7D
Xu 2016 [430]	24/25	CHI_CI versus CI	XueBJ_CI	CI	7D
Xu 2019 [431]	49/49	CHI_CI versus CI	DanSCXQ_CI	CI	10D
Yan 2007 [432]	30/30	CHI_CI versus CI	ShuXN_CI	CI	7–14D
Yan 2014 [433]	55/55	CHI_CI versus CI	TanRQ_CI	CI	7D
Yan 2015 [434]	30/30	CHI_CI versus CI	ChuanKZ_CI	CI	7D
Yan 2017 [435]	39/39	CHI_CI versus CI	DengZHS_YinXDM_CI	CI	14D
Yan 2019 [436]	47/47	CHI_CI versus CI	XueBJ_CI	CI	7D
Yan 2020 [437]	30/30	CHI_CI versus CI	YinX_CI	CI	14D
Yang 2006 [438]	25/25	CHI_CI versus CI	TanRQ_CI	CI	7D
Yang 2008 [439]	30/30	CHI_CI versus CI	TanRQ_CI	CI	10D
Yang 2010a [440]	42/40	CHI_CI versus CI	DanH_CI	CI	14D
Yang 2010b [441]	33/33	CHI_CI versus CI	TanRQ_CI	CI	14D
Yang 2013 [442]	55/55	CHI_CI versus CI	ReDN_CI	CI	7D
Yang 2014 [443]	60/60	CHI_CI versus CI	TanRQ_CI	CI	7D
Yang 2015 [444]	50/50	CHI_CI versus CI	ChuanXQ_CI	CI	10–15D
Yang 2018a [445]	30/30	CHI_CI versus CI	ReDN_CI	CI	10D
Yang 2018b [446]	48/48	CHI_CI versus CI	DengZHS_CI	CI	10D
Yao 2020 [447]	75/75	CHI_CI versus CI	TanRQ_CI	CI	14D
Ye 2009 [448]	30/27	CHI_CI versus CI	XueBJ_CI	CI	10D
Ye 2010 [449]	30/27	CHI_CI versus CI	XueBJ_CI	CI	10D
Ye 2015a [450]	45/45	CHI_CI versus CI	XiXN_CI	CI	7D
Ye 2015b [451]	40/40	CHI_CI versus CI	ShenM_CI	CI	14D
Ye 2016a [452]	40/40	CHI_CI versus CI	ReDN_CI	CI	7D
Ye 2016b [453]	43/43	CHI_CI versus CI	ShenM_CI	CI	7D
Yin 2009a [454]	41/41	CHI_CI versus CI	TanRQ_CI	CI	5D
Yin 2009b [455]	32/30	CHI_CI versus CI	TanRQ_CI	CI	10D
Yin 2011 [456]	32/30	CHI_CI versus CI	TanRQ_CI	CI	10D
Ying 2012 [457]	35/35	CHI_CI versus CI	ShenM_CI	CI	14D
Yu 2014a [458]	52/52	CHI_CI versus CI	TanRQ_CI	CI	14D
Yu 2014b [459]	50/50	CHI_CI versus CI	ReDN_CI	CI	N/A
Yu 2019 [460]	61/60	CHI_CI versus CI	TanRQ_CI	CI	14D
Yu 2020 [461]	50/50	CHI_CI versus CI	TanRQ_CI	CI	7D
Yuan 2008 [462]	60/60	CHI_CI versus CI	ShuangHL_CI	CI	14D
Yuan 2011 [463]	30/30	CHI_CI versus CI	TanRQ_CI	CI	14D
Yuan 2014 [464]	39/39	CHI_CI versus CI	ChuanKZ_CI	CI	7D
Zan 2008 [465]	30/30	CHI_CI versus CI	TanRQ_CI	CI	7D
Zeng 2012a [466]	41/39	CHI_CI versus CI	ShuXN_CI	CI	10–14D
Zeng 2012b [467]	100/96	CHI_CI versus CI	XiXN_CI	CI	7D
Zeng 2014 [468]	114/110	CHI_CI versus CI	ReDN_CI	CI	10D
Zeng 2017 [469]	50/50	CHI_CI versus CI	TanRQ_CI	CI	7–14D
Zeng 2018 [470]	59/59	CHI_CI versus CI	XiYP_CI	CI	14D
Zhai 2012 [471]	40/40	CHI_CI versus CI	TanRQ_CI	CI	7–10D
Zhang 2004 [472]	29/29	CHI_CI versus CI	TanRQ_CI	CI	12D
Zhang 2006a [473]	37/37	CHI_CI versus CI	TanRQ_CI	CI	7D
Zhang2006b [474]	44/44	CHI_CI versus CI	XiXN_CI	CI	15D
Zhang 2008 [475]	21/21	CHI_CI versus CI	ChuanXQ_CI	CI	15D
Zhang 2010 [476]	20/20	CHI_CI versus CI	ShenM_CI	CI	14D
Zhang 2011a [477]	32/30	CHI_CI versus CI	XiYP_CI	CI	10D
Zhang 2011b [478]	45/44	CHI_CI versus CI	ReDN_CI	CI	10D
Zhang 2011c [479]	40/40	CHI_CI versus CI	XiYP_CI	CI	10D
Zhang 2011d [480]	44/44	CHI_CI versus CI	YinXDM_CI	CI	14D
Zhang 2011e [481]	40/34	CHI_CI versus CI	ChuanKZ_CI	CI	7D
Zhang 2012a [482]	45/44	CHI_CI versus CI	ReDN_CI	CI	10D
Zhang 2012b [483]	40/40	CHI_CI versus CI	XueBJ_CI	CI	7D
Zhang 2012c [484]	26/25	CHI_CI versus CI	HuangQ_CI	CI	10D
Zhang 2012d [485]	60/60	CHI_CI versus CI	TanRQ_CI	CI	7D
Zhang 2013 [486]	61/61	CHI_CI versus CI	DanH_CI	CI	14D
Zhang 2014a [487]	40/40	CHI_CI versus CI	XiYP_CI	CI	10D
Zhang 2014b [488]	34/34	CHI_CI versus CI	TanRQ_CI	CI	7D
Zhang 2014c [489]	40/40	CHI_CI versus CI	HongJT_CI	CI	10D
Zhang 2014d [490]	64/64	CHI_CI versus CI	DanH_CI	CI	14D
Zhang 2014e [491]	46/46	CHI_CI versus CI	XiYP_CI	CI	14D
Zhang 2014f [492]	120/120	CHI_CI versus CI	ChuanKZ_CI	CI	7D
Zhang 2015a [493]	43/42	CHI_CI versus CI	ShenF_CI	CI	12D
Zhang 2015b [494]	50/50	CHI_CI versus CI	XueBJ_CI	CI	7D
Zhang 2015c [495]	100/100	CHI_CI versus CI	DengZHS_CI	CI	14D
Zhang 2016a [496]	47/47	CHI_CI versus CI	Hong H	CI	15D
Zhang 2016b [497]	42/35	CHI_CI versus CI	XueBJ_CI	CI	7D
Zhang 2017a [498]	68/68	CHI_CI versus CHI_CI	TanRQ_CI	XiYP_CI	10D
Zhang 2017b [499]	39/39	CHI_CI versus CI	ShenF_CI	CI	14D
Zhang 2017c [500]	45/45	CHI_CI versus CI	XiYP_CI	CI	10D
Zhang 2017d [501]	30/30	CHI_CI versus CI	ZhiCL_CI	CI	7D
Zhang 2018 [502]	44/45	CHI_CI versus CI	TanRQ_CI	CI	N/A
Zhang 2019 [503]	60/60	CHI_CI versus CI	YinXDM_CI	CI	14D
Zhang 2020 [504]	31/31	CHI_CI versus CI	DanSCXQ_CI	CI	7D
Zhang 2021 [505]	63/62	CHI_CI versus CI	DanH_CI	CI	14D
Zhao 2008 [506]	32/32	CHI_CI versus CI	XueBJ_CI	CI	7D
Zhao 2009 [507]	44/40	CHI_CI versus CI	TanRQ_CI	CI	10D
Zhao 2013 [508]	30/30	CHI_CI versus CI	ChuanKZ_CI	CI	14D
Zhao 2014a [509]	26/26	CHI_CI versus CI	ShuXN_CI	CI	14D
Zhao 2014b [510]	40/40	CHI_CI versus CI	ShenM_CI	CI	14D
Zhao 2015 [511]	40/40	CHI_CI versus CI	TanRQ_CI	CI	14D
Zhao 2016a [512]	39/38	CHI_CI versus CI	ChuanKZ_CI	CI	14D
Zhao 2016b [513]	50/50	CHI_CI versus CI	DanH_CI	CI	14D
Zhao 2016c [514]	50/50	CHI_CI versus CI	ShuangHL_CI	CI	10D
Zhao 2019 [515]	49/47	CHI_CI versus CI	XiYP_CI	CI	7D
Zheng 2019 [516]	49/49	CHI_CI versus CHI_CI	TanRQ_DanSCXQ_CI	DanSCXQ_CI	7D
Zheng 2012 [517]	32/33	CHI_CI versus CI	ShenM_CI	CI	14D
Zheng 2015a [518]	30/28	CHI_CI versus CI	ShenQFZ_CI	CI	10D
Zheng 2015b [519]	58/58	CHI_CI versus CI	DanSCXQ_CI	CI	10D
Zheng 2020 [520]	40/40	CHI_CI versus CI	ShenM_CI	CI	14D
Zheng 2021 [521]	63/62	CHI_CI versus CI	DanH_CI	CI	14D
Zhong 2015 [522]	32/32	CHI_CI versus CHI_CI	DanS_CI	DengZHS_CI	10D
Zhong 2017 [523]	33/33	CHI_CI versus CI	ShenF_CI	CI	7D
Zhong 2019 [524]	132/132	CHI_CI versus CI	TanRQ_CI	CI	14D
Zhou 2008a [525]	24/24	CHI_CI versus CI	XueBJ_CI	CI	14D
Zhou 2008b [526]	43/38	CHI_CI versus CI	TanRQ_CI	CI	14D
Zhou 2009a [527]	40/40	CHI_CI versus CI	XiXN_CI	CI	14D
Zhou 2009b [528]	72/50	CHI_CI versus CI	TanRQ_CI	CI	10–14D
Zhou 2014 [529]	30/30	CHI_CI versus CI	ReDN_CI	CI	7D
Zhou 2016a [530]	54/54	CHI_CI versus CI	DanH_CI	CI	14D
Zhou 2016b [531]	30/30	CHI_CI versus CI	HuangQ_CI	CI	14D
Zhu 2010a [532]	77/77	CHI_CI versus CI	XiXN_CI	CI	N/A
Zhu 2010b [533]	77/77	CHI_CI versus CI	XiXN_CI	CI	N/A
Zhu 2016 [534]	38/38	CHI_CI versus CI	XueBJ_CI	CI	8D
Zhu 2017 [535]	44/44	CHI_CI versus CI	DanSCXQ_CI	CI	14D
Zhu 2018 [536]	40/40	CHI_CI versus CI	DanH_CI	CI	14D
Zhu 2020 [537]	100/100	CHI_CI versus CI	XingNJ_CI	CI	7D
Zou 2011 [538]	60/60	CHI_CI versus CI	TanRQ_CI	CI	10D
Zhou 2014 [539]	40/40	CHI_CI versus CI	DanSDFSY_CI	CI	14D
Zuo 2010 [540]	30/30	CHI_CI versus CI	DanH_CI	CI	7D

**Table 4.** Characteristics of included publications.

Characteristics		Number (contents)
Total number included	513	
Type of publication		
Regulatory guidance	4	
WHO guidance	1 WHO’s definition and requirements for Essential Medicines [541]	
China’s essential medicines system policy	1 Opinions on Improving the National Essential Medicines System [542]	
China’s basic medical insurance drug management policy	0	
China’s good clinical practice for drug trial quality management standards	0	
Chinese drug supervision and management policies and regulations	1	General Considerations for the Conduct of Clinical Trials with Medicinal Products (2017) [543]
FDA regulatory documents	0	
EMA regulatory documents	1 EMA guideline [544]	
Guidelines and expert consensus	9	
Guidelines	7	GOLD 2021 [545], AAFP guideline [546], Swiss Guideline 2018 [547], European and American Society Guideline 2017 [548], American and Canadian Society Guideline 2022 [549], Chinese Guideline 2021 [550], TCM guideline 2018 [551],
Expert consensus	2	European Expert Consensus 2021 [552], Chinese Expert Consensus 2017 [553]
COS study	1	
ERS statement [45]	1	
RCTs	499	
Methods		
Initial literature (trials)	499	
Systematic review	10	ERS Statement [45], GOLD 2021 [545], AAFP guideline [546], European Expert Consensus 2021 [552], Swiss Guideline 2018 [547], European and American Society Guideline 2017 [548], American and Canadian Society Guideline 2022 [549], Chinese Expert Consensus 2017 [553], Chinese Guideline 2021 [550], TCM guideline 2018 [551]
Interviews/focus groups	0	
Delphi/ NGT/ other consensus group meeting	10	ERS Statement [45], GOLD 2021 [545], AAFP guideline [546], European Expert Consensus 2021 [552], Swiss Guideline 2018 [547], European and American Society Guideline 2017 [548], American and Canadian Society Guideline 2022 [549], Chinese Expert Consensus 2017 [553], Chinese Guideline 2021 [550], TCM guideline 2018 [551]
Patient involvement	1	ERS Statement [45]
HCP involvement	12	ERS Statement [45], GOLD 2021 [545], AAFP guideline [546], European Expert Consensus 2021 [552], Swiss Guideline 2018 [547], European and American Society Guideline 2017 [548], American and Canadian Society Guideline 2022 [549], Chinese Expert Consensus 2017 [553], Chinese Guideline 2021 [550], TCM guideline 2018 [551], EMA guideline 2012 [544], General Considerations for the Conduct of Clinical Trials with Medicinal Products (2017) [543]

Note: WHO: World Health Organization; EMA: European Medicines Agency; FDA: Food and Drug Administration; HCP: health care professionals; ERS: European Respiratory Society.

#### Quality assessment

##### Quality assessment of COS research

The included COS study was conducted and reported following the COMET handbook [23], COS-STAD [31], and STAndards [554] for Reporting. Delphi survey and consensus meetings were conducted, and patients were involved in the COS development. The included study provided adequate information about scope (COS-STAD items 1–4), stakeholders Involved (COS-STAD items 5–7), and consensus process (COS-STAD items 8–11).

##### Quality assessment of guidelines and expert consensus

The methodological quality of the 7 guidelines and 2 expert consensus was evaluated. The scores indicated that the highest ratings were achieved in the areas of ‘Scope and Purpose of the Guidelines’ and ‘Clarity of Guideline Expression’, while the lowest ratings were seen in the areas of ‘Applicability of the Guidelines’ and ‘Rigour of Guideline Development’.

In terms of ‘Scope and Purpose of the Guidelines’, the average score was 69%. The GOLD guideline, AAFP guideline, and the 2021 European expert consensus demonstrated excellent clarity in scope and purpose.

Regarding the ‘Guideline Development Panel’, the average score was 50%. The AAFP guideline performed well in terms of panel composition.

In the area of ‘Rigour of Guideline Development’, the average score was 48%. Both the AAFP guideline and the Traditional Chinese Medicine Diagnosis and Treatment Guideline for Chronic Obstructive Pulmonary Disease (2018 edition) exhibited strong rigor in their development.

As for ‘Clarity of Guideline Expression’, the average score was 74%. The GOLD guideline, AAFP guideline, and the management guideline for exacerbations of COPD provided clear and concise expression. However, three guidelines developed by Chinese scholars were criticized for having ambiguous and less clear expressions, making them harder to understand.

In terms of ‘Applicability of the Guidelines’, the average score was 36%. The GOLD guideline and the AAFP guideline were deemed more applicable.

Regarding the ‘Independence of Guideline Editors’, the average score was 58%. The management guideline for exacerbations of COPD showed good independence of editors.

Furthermore, two Western medicine guidelines from China lacked clear methodological reporting, failing to provide detailed information on literature search methods and recommendation formation. On the other hand, the Traditional Chinese Medicine guideline and the GOLD guideline provided clearer methodological reporting.

Overall, 7 studies were rated as Level B, originating from the United States, Europe, Canada, and China. These studies’ recommendations can be recommended in clinical practice with slight modifications. Additionally, there were two guidelines rated as Level C, which are two revised medicine guidelines (Expert Consensus on Diagnosis and Treatment of Acute Exacerbation of Chronic Obstructive Pulmonary Disease (2017 revised edition) and Diagnosis and Treatment Guideline for COPD 2021). Extra caution is needed when considering recommendations from these guidelines. Finally, the three evaluators showed good consistency, with ICC values greater than 0.05 and P-values less than 0.05. Specific numerical values can be found in Table 5.

**Table 5.** The quality assessment of included AECOPD guidelines and expert consensus.

Guidelines and expert consensus	GOLD 2021 [545]	AAFP guideline [546]	European Expert Consensus 2021 [552]	Chinese Expert Consensus 2017 [553]	Chinese Guideline 2021 [550]	TCM guideline 2018 [551]	European and American Society Guideline 2017 [548]	American and Canadian Society Guideline 2022 [549]	Swiss Guideline 2018 [547]	Mean
Domain 1 Scope and Purpose	96%	93%	65%	33%	20%	61%	91%	80%	85%	69%
Item 1 Clearly states the overall objective of the guideline	7.00	6.67	5.33	1.67	1.67	5.33	7.00	7.00	7.00	
Item 2 Clearly describes the healthcare questions addressed by the guideline	7.00	6.67	5.33	5.33	3.33	6.00	6.33	6.00	5.33	
Item 3 Clearly describes the target population, including patients, the public, etc.	6.33	6.33	4.00	2.00	1.67	2.67	6.00	6.33	6.00	
Domain 2 Stakeholder Involvement	59%	87%	35%	7%	15%	48%	67%	61%	67%	50%
Item 4 The guideline development group includes individuals from all relevant professional groups	6.67	6.33	3.33	2.33	2.67	5.67	7.00	6.67	7.00	
Item 5 The guideline considers the values and preferences of the target population (patients, the public, etc.)	1.33	6.33	0.67	1.00	1.33	1.00	1.33	1.33	1.33	
Item 6 The users of the guideline are clearly identified and described	5.67	6.00	5.33	1.00	1.67	5.00	6.67	6.00	6.67	
Domain 3 Rigor of Development	64%	85%	23%	19%	20%	82%	87%	34%	21%	48%
Item 7 The guideline uses a systematic approach to search for existing evidence	6.33	5.67	2.33	2.00	1.33	6.33	6.67	2.00	2.00	
Item 8 The guideline explicitly states and reports the criteria used for selecting the evidence	2.67	5.33	2.00	2.00	1.67	6.67	6.67	7.00	2.67	
Item 9 The guideline clearly describes the strengths and limitations of the body of evidence	2.67	6.67	1.00	2.33	1.33	6.00	6.33	1.33	1.33	
Item 10 The guideline clearly describes the methods and processes used to formulate the final recommendations	5.67	5.67	6.67	1.33	1.67	6.33	6.67	5.67	2.67	
Item 11 The guideline considers the health outcomes, benefits, and risks, as well as potential side effects for the target population when formulating recommendations	6.67	5.67	2.33	2.33	1.33	4.33	6.00	2.33	2.00	
Item 12 There is a clear link between the guideline’s recommendations and the supporting evidence	7.00	6.67	2.33	3.33	2.33	6.00	6.00	2.33	2.00	
Item 13 The guideline undergoes external review by relevant experts before publication	1.67	6.33	1.33	2.00	2.67	5.33	5.67	2.33	1.00	
Item 14 The guideline provides a process for updating the guideline	6.00	6.67	1.00	1.67	5.33	6.33	5.67	1.33	4.33	
Domain 4: Clarity of Expression	98%	87%	69%	31%	50%	80%	100%	78%	76%	74%
Item 15: The recommendations in this guideline are clear and unambiguous	6.67	6.33	5.00	2.67	2.00	5.67	7.00	6.33	6.33	
Item 16: The guideline clearly presents different options for a specific situation or health issue	7.00	5.67	5.33	3.00	5.33	6.00	7.00	4.67	5.00	
Item 17: The main recommendations in this guideline are clear and easily identifiable	7.00	6.67	5.00	3.00	4.67	5.67	7.00	6.00	5.33	
Domain 5: Applicability	56%	57%	32%	24%	36%	4%	53%	38%	26%	36%
Item 18: The guideline describes factors that promote or hinder the actual application of the guideline	2.33	6.33	3.33	1.00	0.67	1.00	4.33	4.33	3.33	
Item 19: The guideline provides specific recommendations and/or corresponding tools on how to apply the recommendations in clinical practice	3.00	5.33	4.67	2.33	4.67	1.67	5.00	1.33	1.67	
Item 20: The guideline considers potential health economic issues such as resource implications when applying the recommended practices	5.33	3.67	2.33	1.33	2.33	1.33	3.33	2.33	3.00	
Item 21: The guideline provides specific criteria for monitoring and/or auditing to be followed	6.67	2.33	1.33	5.00	5.00	1.00	4.00	5.00	2.33	
Domain 6: Editorial Independence	22%	72%	47%	11%	25%	86%	92%	86%	86%	58%
Item 22: The views of funding sources do not influence the content and recommendations of the guideline	3.00	4.00	2.33	2.33	3.00	6.00	6.33	5.33	5.33	
Item 23: The guideline extensively documents and considers conflicts of interest among the members of the development group	1.67	6.67	5.33	1.00	2.00	6.33	6.67	7.00	7.00	
Recommendation Level	B	B	B	C	C	B	B	B	B	
ICC	0.734	0.549	0.711	0.088	0.503	0.291	0.512	0.422	0.481	

Note: Scores in the table represent the average ratings given by three evaluators. The percentage represents the standardized percentage of the maximum possible score for each domain. ICC: intra-class correlation coefficient.

##### Quality evaluation of RCTs

**Figure 1.**
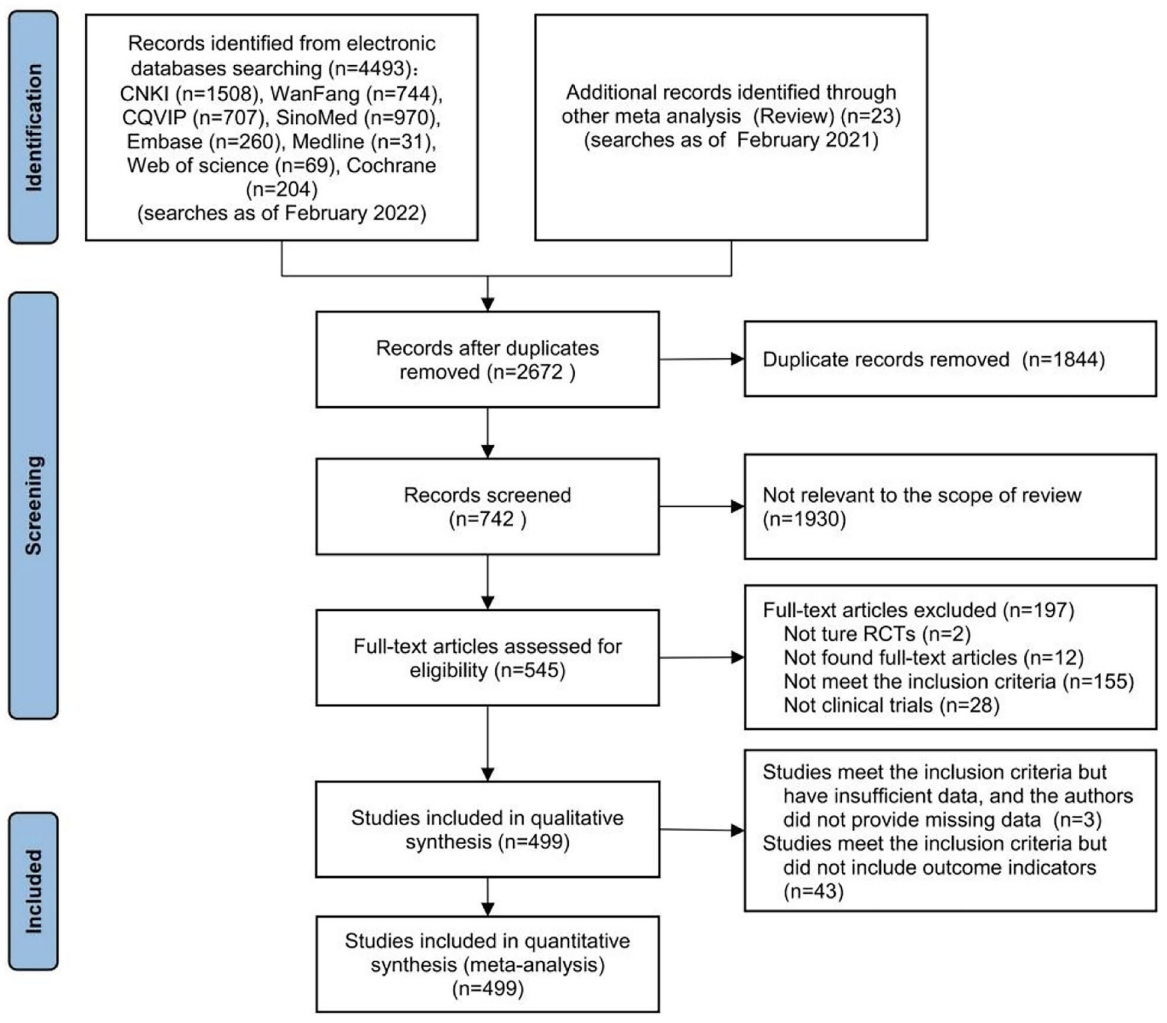
The flowchart of meta-analysis.

**Figure 2.**
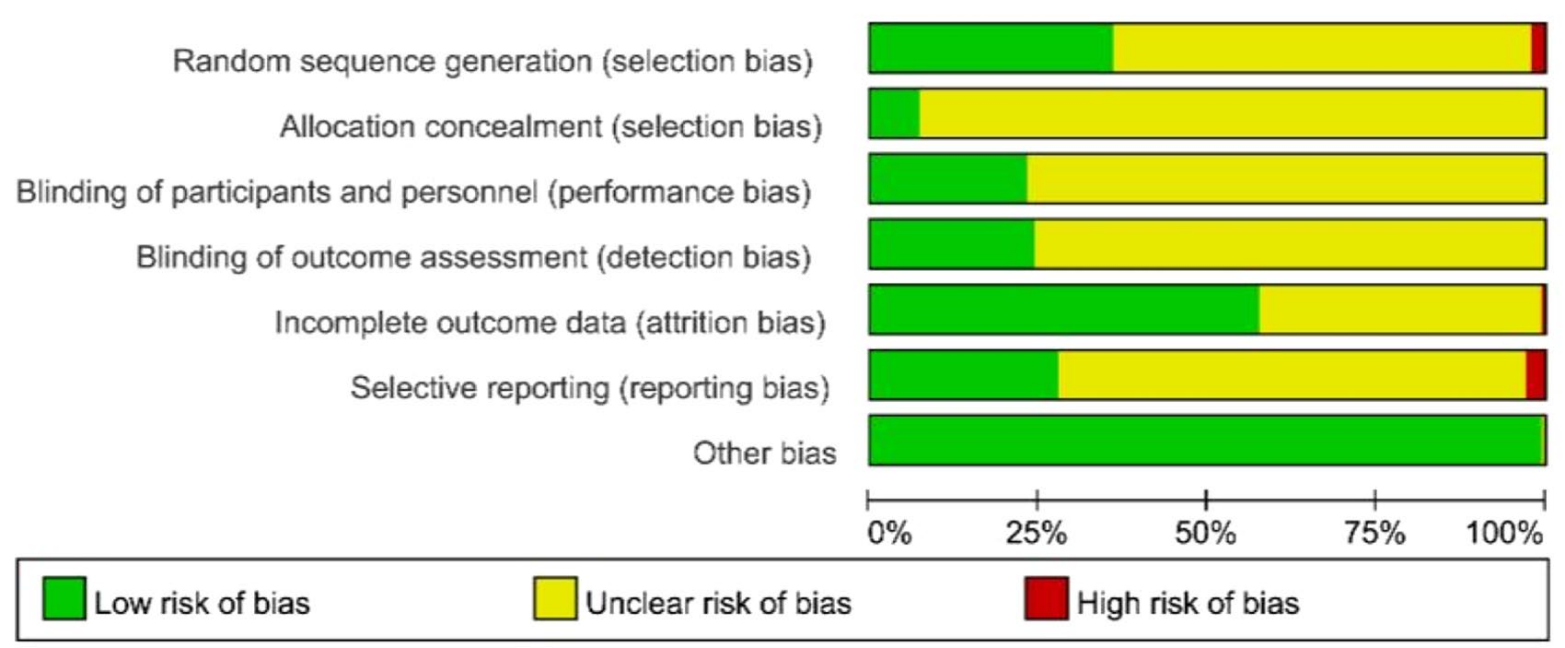
ROB assessment of included RCTs.

Please see Figure 1 for the flow diagram of meta-analysis and Figure 2 for the risk of bias assessment for the included 499 RCTs. The allocation sequence generation method was explicitly reported in 179 trials, indicating a low risk of bias. However, in 310 trials, the method was not described, leading to an unclear risk of bias. Ten trials reported non-random allocation sequences, indicating a high risk of bias. Allocation concealment was explicitly reported in 34 trials, indicating a low risk of bias, while 464 trials lacked this information, resulting in an unclear risk of bias. Blinding of participants and outcome assessments was unclear in many trials. Incomplete outcome data were adequately addressed in 325 trials, but 172 trials had insufficient information. Selective reporting bias was observed in some trials. Overall, most trials had an unclear risk of bias due to insufficient information.

### Selection of core outcomes

#### Recommendations from regulatory guidance documents

##### WHO guidance

Unfortunately, we were unable to locate any regulatory guidance documents pertaining to outcomes for clinical trials on the official WHO website. However, we have taken into account the definition and requirements for ‘Essential Medicines’ provided by the WHO [541] in order to establish the domains or dimensions that should be encompassed by our drug clinical evaluation outcomes.

The WHO defines ‘Essential Medicines’ as drugs that meet the fundamental health needs of individuals, selected based on evidence of their effectiveness, safety, and cost-effectiveness in light of the current public health situation. Based on this understanding, we have identified efficacy, safety, and health economics as the three key dimensions that should be emphasized when conducting clinical evaluations for our drug.

##### China’s essential medicines system policy

The State Council General Office recently issued the document ‘Opinions on Improving the National Essential Medicines System’ [542], which provides clear guidelines for the role and selection of essential medicines in China. The policy emphasizes the importance of essential medicines in meeting healthcare needs, ensuring availability, prioritizing use, maintaining quality, and reducing financial burdens on patients. The policy prioritizes basic drugs with proven efficacy, safety, and cost-effectiveness, while also addressing the withdrawal of market-discontinued drugs, those causing severe adverse reactions, or those with superior alternatives. This aligns closely with WHO’s emphasis on efficacy, safety, and health economics in clinical evaluation.

##### Chinese drug supervision and management policies and regulations

The ‘General Considerations for the Conduct of Clinical Trials with Medicinal Products’ [543] does not provide specific recommendations for clinical outcomes in AECOPD, but offer specific AECOPD clinical outcome recommendations but provides guidance on outcome selection. It states that outcomes in trials should be clearly defined, specifying their attributes and observation methods. Primary outcomes should reflect main clinical effects aligned with study objectives, while secondary outcomes assess additional drug effects. Trial outcomes and analysis plans must be pre-specified in protocols. Surrogate outcomes can be primary only if they reliably predict clinical benefits. Evaluation methods should be accurate, precise, and responsive over time, whether subjective or objective.

##### EMA guideline

According to the EMA Guideline [544] for COPD, outcomes are divided into efficacy endpoints and safety outcomes. The efficacy endpoints include lung function, exacerbation, patients’ and investigators’ reported outcomes, health status and health-related quality of life (HRQoL), dyspnea, COPD symptom scales, patients’ questionnaires or diary cards, exercise capacity, rescue medication, composite scores, imaging CT (Computerized Tomography), and other potential outcomes such as physical activity and biomarkers of systemic inflammation. Safety outcomes include specific safety concerns, extent of exposure, and long-term safety data. Specific complete issues include all-cause mortality, Body mass index, Obstruction, Dyspnea, and Exercise capacity (BODE) index, cardiovascular adverse effects when combined with long-acting beta2 agonists and anticholinergics, and incidence of pneumonia or lower respiratory tract infection, and exposure and long-term safety data should be reliable, prospective safety data for at least 1 year of treatment.

We were unable to locate any specific documents or information related to China’s Basic Medical Insurance Drug Management Policy, China’s GCP for Drug Trial Quality Management Standards, and FDA regulatory documents.

### Recommendations from the COS study

The core outcome set recommended by COS research [45] includes death, treatment success, need for higher-level care, arterial blood gas levels, patient-reported outcomes, future impact, safety, and treatment adherence. Measurement instruments are proposed (see Table 6). Both all-cause mortality and AECOPD-specific mortality are considered. Treatment success is defined as significant symptom improvement without additional systemic treatment. Need for higher-level care includes hospitalization and ICU admission. Patient-reported outcomes cover symptoms and quality of life. Future impact assessment includes disease progression and AECOPD recurrence. Safety evaluation includes drug-resistant bacteria and pneumonia progression. Treatment adherence assessment is emphasized for intervention-specific outcomes.

**Table 6.** Recommendations from COS study.

Outcome		Measure instrument
Death		
	Death from any cause	
	Death from a COPD exacerbation	
Treatment success (sufficient improvement of the signs and symptoms of the exacerbation that no additional systemic treatments (antibiotics or systemic corticosteroids) are required)		
Need for higher level of care		
	Need for hospital admission for the presenting exacerbation	
	Need for admission to the intensive care unit for the exacerbation	
Levels of oxygen and carbon dioxide in the blood (arterial blood gases)		
Patient reported outcomes		
	Breathlessness	The modified Borg’s scale
	Health related quality of life	The COPD Assessment Test (CAT)
	Activities of daily living	The Capacity of Daily Living in the Morning Questionnaire (CDLM) and The Manchester Activities of Daily Living Questionnaire (MRADL)
	Worsening of symptoms after the initial treatment	The modified Borg’s scale and the COPD assessment test(CAT)
Future Impact		
	Disease progression	Lung function (Forced Expiratory Volume in 1 second (FEV_1_), forced vital capacity (FVC) and FEV_1_/FVC ratio)
		The number of exacerbations
	Future exacerbations	The number of exacerbations after treatment success is confirmed
	Future hospital admissions	
Safety		
	Serious adverse events from treatments	
	Development of resistant bacteria	Bacterial resistance to the administered antibiotics in spontaneous sputum
	Development of pneumonia	The presence of new consolidation in the chest x-ray or other imaging modalities of the chest
Treatment adherence		Clearly reported intervention specific outcome

Note: The greened-out items represent domains and outcomes suggested by COS study, regulatory guidance documents, guidelines and expert consensus, and RCTs.

### Guidelines and consensus-recommended outcomes

AECOPD refers to the acute exacerbation of symptoms in individuals with pre-existing COPD. The assessment of AECOPD is often based on the evaluation of COPD itself. Therefore, this study provides a comprehensive summary of the assessment outcomes for both COPD and AECOPD.

The recommended outcomes do not include any that are solely endorsed by two guidelines/consensus as grade C. Each guideline also focuses on patient-centered outcomes such as mortality, treatment failure/success, future impact, complications, and quality of life. The following measures are recommended to define treatment failure outcomes: mortality rate after 30 days of discharge, readmission rate to the intensive care unit, readmission rate, re-use of antibiotics or corticosteroids for AECOPD, occurrence of new complications, and an increase in short-acting inhaler therapy.

‘GOLD 2021’ emphasizes airflow limitation severity, health impairment, and future risk of events. All guidelines stress airflow limitation, symptom evaluation, exacerbation risk, and comorbidity assessment.

Additionally, the included guidelines and expert consensus recommend physical signs, lab tests, exacerbation severity, exercise capacity, complications, and disease management evaluation, along with predicting long-term survival. For detailed assessment content, indicators, and evidence sources, see Table 5.

### Outcomes reported in RCTs

In 499 RCT [6, 46–540] reports, various outcomes were examined, including mortality, treatment outcomes, future impact, higher-level care needs, complications, airflow limitation assessment, symptom evaluation, lab tests, exercise capacity, disease management, oxygen therapy, antibiotic treatment, safety, and health economics. Lab tests covered arterial blood gases, pulmonary function, leukocyte count, cytokines, oxidative stress markers, immune factors, hemodynamics, and others. Disease management included oxygen and antibiotic evaluation. 361 studies used custom composite endpoints, 32 developed symptom scoring systems, and 20 used TCM syndrome scales. See Table 7 for detailed evaluation domains and outcomes.

**Table 7.** Outcomes retrieved from the included RCTs.

Domain	Outcome	Number of reported RCT
Death		
	All-cause mortality	6
Treatment success/failure		
	Efficiency (customized composite outcome)	361
	Readmission rate (within 3 months)	2
Future impact		
	Future exacerbation	
	Future AECOPD exacerbation (within 3 months)	1
	Disease progression	
	FEV1%	52
	FVC	79
	FEV1/FVC	111
Need for higher level of care		
	Length of stay in the ICU	8
Complication		
	Incidence of complications	3
Assessment of airway limitation severity		
	FEV_1_	108
Health related quality of life		
	COPD assessment test (CAT) score	16
Symptom and physical signs assessment		
	The time for clinical symptoms and signs to disappear or resolve	25
	Length of hospital stay	39
	Duration of fever	5
	Improvement rate of symptoms and signs	1
	Score of symptoms and signs assessment	32
	Acute Physiology and Chronic Health Evaluation II (APACHE II) score	8
	Body Mass Index (BMI)	2
	Traditional Chinese Medicine Syndrome Score (assessed by customized scale)	20
	Body-Mass Index, Airflow Obstruction, Dyspnea, and Exercise Capacity (BODE) score	1
	Modified British Medical Research Council (mMRC) score	5
	St. George’s Respiratory Questionnaire (SGRQ) score	3
	Clinical Pulmonary Infection Score (CPIS)	3
	Asthma Quality of Life Questionnaire (AQLQ) assessment	1
	National Institutes of Health Stroke Scale (NIHSS) score	1
Laboratory tests		
	Arterial blood gas analysis	
	Partial pressure of carbon dioxide (PaCO_2_)	195
	Partial pressure of oxygen (PaO_2_)	190
	Oxygen saturation (SaO_2_)	28
	Potential of hydrogen (PH)	88
	Oxygenation index (PaO2/FiO2)	3
	Pulmonary function	
	Peak expiratory flow (PEF)	17
	Residual volume (RV)	2
	Residual volume to total lung capacity ratio (RV/TLC)	2
	Maximal mid-expiratory flow (MMEF)	4
	White blood cell	
	WBCC	63
	White blood cell classification	1
	Granulocyte percentage (GR%)	39
	Neutrophil classification	2
	Cytokines	
	C-reactive protein (CRP)	109
	Time to normalize CRP	1
	High-sensitivity C-reactive protein (hs-CRP)	10
	Procalcitonin (PCT)	42
	Eosinophils (EOS)	1
	Interleukin-1 (IL-1)	3
	Interleukin-1 beta (IL-1β)	8
	Interleukin-2 (IL-2)	3
	Interleukin-4 (IL-4)	3
	Interleukin-6 (IL-6)	32
	Interleukin-8 (IL-8)	46
	Interleukin-10 (IL-10)	16
	Interleukin-13 (IL-13)	1
	Interleukin-17 (IL-17)	7
	Interleukin-18 (IL-18)	7
	Tumor necrosis factor-alpha (TNF-a)	63
	Endotoxin (ET)	2
	NF-κB DNA binding activity	4
	Soluble triggering receptor expressed on myeloid cells 1 (sTREM-1)	3
	Neuron-specific enolase (NSE)	1
	Matrix metalloproteinase-9 (MMP-9)	4
	Transforming growth factor-beta (TGF-β)	4
	Tissue inhibitor of metalloproteinases-1 (TIMP-1)	2
	Calcitonin gene-related peptide(CGRP)	4
	Leukotriene B4 (LTB-4)	2
	Surfactant protein D (SP-D)	3
	Toll-like receptor 4 (TLR4)	3
	8-isoprostane prostaglandin F2-alpha (8-iso-PGF2α)	6
	Endothelin-1 (ET-1)	8
	Nitric oxide (NO)	5
	Serum amyloid A (SAA)	2
	Alpha-1-acid glycoprotein (AAG)	1
	Endothelial cell selectin	1
	Vascular cell adhesion molecule-1 (VCAM-1)	2
	Intercellular adhesion molecule-1 (ICAM-1)	4
	Myeloperoxidase (MPO)	2
	Fibroblast growth factors (FGFs)	1
	Hypoxia-inducible factor-1 alpha protein expression level (HIF-1a)	1
	C-C motif chemokine ligand 18(CCL18)	1
	N-terminal pro-brain natriuretic peptide (NT-proBNP)	1
	Brain natriuretic peptide (BNP)	2
	Oxidative stress markers	
	Superoxide dismutase (SOD)	6
	Malondialdehyde (MDA)	6
	Reduced glutathione (GSH)	1
	Glutathione peroxidase (GSH-Px)	3
	Glutathione S-transferase (GSH-ST)	1
	Vitamin E (VitE)	1
	Lipid peroxidation (LPO)	2
	Catalase	1
	Total antioxidant capacity (T-AOC)	1
	4-Hydroxynonenal (HNE)	1
	T lymphocyte subset determination	
	Total T lymphocytes (CD3+)	14
	Total lymphocytes (CD45+)	1
	Helper T cells (CD3 + CD4+)	18
	Cytotoxic T cells (CD3 + CD8+)	16
	Ratio of T helper cells to T suppressor cells (CD3 + CD4+/CD3 + CD8+)	13
	NK cells (CD3-CD16 + CD56+)	4
	B cells (CD3-CD19+)	1
	Immunoglobulins	
	Immunoglobulin M	6
	Immunoglobulin G	6
	Immunoglobulin A	6
	Red blood cell immune function: Red blood cell C3b receptor rosette (RBC-C3bR) Red blood cell immune complex rosette (RBC-ICR) Rate of red blood cell C3b receptor rosetting promoting the red blood cell immune regulator (RFER) Red blood cell C3b receptor rosette inhibition by the red blood cell immune regulator (RFIR)	2
	Hemorheology	
	Whole blood viscosity	1
	Whole blood reduced viscosity	1
	Plasma viscosity	1
	Blood plasma viscosity	2
	Hematocrit (HCT)	14
	Whole blood low shear viscosity	6
	Whole blood high shear viscosity	6
	Red blood cell aggregation index	4
	Fibrinogen (FBG)	13
	Erythrocyte sedimentation rate (ESR)	2
	Coagulation function	
	Prothrombin time (PT)	9
	Thrombin time (TT)	6
	Activated partial thromboplastin time (APTT)	9
	Platelet count (PLT)	3
	Tissue factor (TF)	2
	Tissue factor pathway inhibitor (TFPI)	1
	D-dimer	7
	Thrombomodulin	5
	von Willebrand factor (vWF)	1
	Platelet aggregation rate	
	Analysis of gut microbiota diversity	1
	Chest X-ray examination	31
	Computed tomography of the chest	2
	Hemodynamics	
	The internal diameter of the right ventricular outflow tract	2
	Cardiac output (CO)	2
	Left ventricular ejection fraction (LVEF)	5
	Pulmonary artery systolic pressure (PASP)	8
	Pulmonary artery diastolic pressure (PADP)	5
	Mean pulmonary artery pressure (MPAP)	8
	Pulmonary vascular resistance (PVR)	1
Assessment of exercise capacity		
	6-minute walk test (6-minute walking distance)	5
Assessment of the treatment of the disease		
Oxygen therapy		
	Average non-invasive positive pressure ventilation (NIPPV) time	1
	Respirator parameters (respiratory mechanics indicators)	2
	Pressure support ventilation (PSV)	2
	Positive end-expiratory pressure (PEEP)	1
	Number of endotracheal intubations	1
	Mechanical ventilation time (invasive ventilation time + non-invasive ventilation time)	12
	Invasive mechanical ventilation time	5
	Weaning success rate/failure rate	9
	Ventilator-associated pneumonia (VAP) incidence rate	3
	Incidence rate of ventilator-associated pneumonia	1
	Rate of re-intubation after extubation	1
Use of antibiotics		
	Duration of intravenous antibiotic administration	4
Safety		
	Adverse event incidence rate	59
	Sputum culture (assessment of drug-resistant bacteria)	12
	Vital signs: respiration, heart rate, blood pressure, and oxygen saturation	22
	Complete blood count	68
	Urinalysis	36
	Stool analysis	29
	Liver function test	48
	Kidney function test	48
	Electrocardiogram	21
Healthcare economics		
	Hospitalization costs	3

Note: Outcome measures with a blue background are further subjected to network meta-analysis.

ICU: Intensive care unit.

NF-κB: Nuclear factor-kappa B.

DNA: Deoxyribonucleic acid.

In summary, commonly recommended outcome domains and specific outcomes from literature sources of the above four steps are as follows:

1. Death (all causes mortality)
2. Treatment success/failure
3. Future impact (future AECOPD exacerbation)
4. Safety (the proportion of adverse events)
5. Health-related quality of life
  – CAT (COPD Assessment Test)
  – SF-36 (Short Form 36)
  – EADL (European Academy of Dermatology and Venereology Life Quality Index)

There were variations in the definition of treatment success/failure and the recommended tools for assessing health-related quality of life among different sources of literature.

However, some commonly reported outcomes fall into different domains, such as:

- Oxygen and carbon dioxide levels in the blood (arterial blood gases)
- Lung function measurements (FEV_1_, FVC, FEV_1_/FVC ratio)
- Patient-reported outcomes (breathlessness)

To ensure comprehensive coverage, we have selected commonly reported outcome domains from different literature sources and outcomes that appeared in the included RCTs and outcomes from included RCTs with a frequency exceeding 6% as core outcomes (highlighted in blue in Table 8).

**Table 8.** Recommended evaluation domains, outcomes, and measurement tools from regulatory guidance documents, guidelines and expert consensus.

Domain	Outcomes and measurement tools	Sources of evidence (type of studies)	Recommended guidelines and/or consensus
Death	All-cause mortality	RCT, systematic Review (Moderate quality evidence), Delphi survey	GOLD 2021 [545], AAFP guideline [546], European Expert Consensus 2021 [552], Swiss Guideline 2018 [547], European and American Society Guideline 2017 [548], American and Canadian Society Guideline 2022 [549], Chinese Guideline 2021 [550], Chinese Expert Consensus 2017 [553]
	Survival time	Delphi survey, systematic review	European Expert Consensus 2021 [552], European and American Society Guideline 2017 [548]
Treatment success/failure	It can be defined as death, readmission to the intensive care unit, re-hospitalization, reuse of antibiotics or steroids for AECOPD, new complications, or an increase in short-acting inhaled treatments (after 30 days of discharge)	Delphi survey, systematic review	European Expert Consensus 2021 [552], European and American Society Guideline 2017 [548]
Future impact	Future exacerbation: The number of exacerbation	Observational study, Delphi survey, cross-sectional survey, RCT, systematic review	GOLD 2021 [545], European Expert Consensus 2021 [552], Swiss Guideline 2018 [547], American and Canadian Society Guideline 2022 [549], Chinese Guideline 2021 [550], Chinese Expert Consensus 2017 [553],
Complications	Incidence of complications	Systematic review	European and American Society Guideline 2017 [548]
	Chest CT scan	Unreported	GOLD 2021 [545]
Health related quality of life	Quality of life scale score	Systematic review	European and American Society Guideline 2017 [548]
	Health status and health-related quality of life (HRQoL, SF-36, EADL)	Unreported	EMA guideline 2012 [544]
Assessment of airway limitation severity	Forced Expiratory Volume in 1 second (FEV_1_) after bronchodilator use	Retrospective cohort study	GOLD 2021 [545], Swiss Guideline 2018 [547], Chinese Guideline 2021 [550], EMA guideline 2012 [544]
	FEV_1_, Inspiratory Capacity (IC), Functional Residual Capacity (FRC), Residual Volume to Total Lung Capacity ratio (RV/TLC), Forced Vital Capacity (FVC), Slow Vital Capacity, and Diffusing Capacity of the Lungs for Carbon Monoxide (DLCO).	Unreported	EMA guideline 2012 [544]
Symptom assessment	Modified British medical research council (mMRC)	Perspective cohort study, Delphi survey	GOLD 2021 [545], European Expert Consensus 2021 [552], Swiss Guideline 2018 [547], Chinese Guideline 2021 [550]
	COPD Assessment test (CAT)	Systematic review, Delphi survey	GOLD 2021 [545], European Expert Consensus 2021 [552], Swiss Guideline 2018 [547], Chinese Guideline 2021 [550]
	The Chronic Respiratory Disease Questionnaire (CRQ) and the St. George’s Respiratory Questionnaire (SGRQ) (more complex, lower clinical applicability, not recommended)	Unreported	GOLD 2021 [545]
	The indices of breathlessness, including the Clinical Dyspnea Scale, Baseline Dyspnea Index (BDI), Transitional Dyspnea Index (TDI), and the measure of breathlessness in the Chronic Respiratory Questionnaire (CRQ)	Unreported	EMA guideline 2012 [544]
	The Borg Category Ratio Scale for Rating Perceived Breathlessness (CR10) and the Visual Analog Scale (VAS)	Unreported	EMA guideline 2012 [544]
	The Chronic Obstructive Pulmonary Disease (COPD) Symptom Scale (Symptoms to be recorded include: nighttime symptoms, nighttime awakenings, daytime symptoms, cough, wheezing, breathlessness, and sputum production)	Unreported	EMA guideline 2012 [544]
	Patient-reported and investigator-reported outcomes (including disease-specific questionnaires, breathlessness, and symptom scales)	Unreported	EMA guideline 2012 [544]
	Comprehensive Score (BODE Index, airflow obstruction measured by FEV_1_, breathlessness assessed by Dyspnea Scale, exercise capacity measured by 6-minute walk distance composite score)	Unreported	EMA guideline 2012 [544]
	Symptoms that must be collected include: wheezing, increased sputum production, sepsis, cough, fever, use of rescue medications, decreased exercise tolerance (assessed using a scale), delirium, loss of consciousness, and orthopnea.	Delphi survey	European Expert Consensus 2021 [552]
	Symptoms advised to be collected include: chest tightness, chest pain, hemoptysis, nighttime cough, muscle pain, fatigue, excessive sleepiness, poor appetite, palpitations, sore throat, nasal congestion, rhinorrhea, and headache.	Delphi survey	European Expert Consensus 2021 [552], Chinese Expert Consensus 2017 [553]
Physical signs assessment	Blood oxygen saturation, oxygen supplementation, heart rate, respiratory rate, hemodynamics, use of accessory respiratory muscles, improvement in mental status, blood pressure, body temperature, sputum color, Body Mass Index (BMI), and weight	Delphi survey	European Expert Consensus 2021 [552], Chinese Expert Consensus 2017 [553]
Laboratory tests	Mandatory tests: complete blood count, urinalysis, electrolytes, creatinine, troponin, brain natriuretic peptide, blood glucose, liver function test, arterial blood gas analysis, chest X-ray, electrocardiogram, echocardiogram, sputum culture, influenza virus throat swab	Delphi survey	European Expert Consensus 2021 [552], Chinese Expert Consensus 2017 [553]
	Biomarkers of systemic inflammation CT imaging (changes in lung parenchyma)	Unreported	EMA guideline 2012 [544]
The evaluation of the severity of acute exacerbation	The assessment of mild, moderate, and severe exacerbation can be based on the treatment measures required.	Unreported	GOLD 2021 [545], Chinese Guideline 2021 [550], Chinese Expert Consensus 2017 [553]
	Evaluate the frequency and/or severity of acute exacerbations (reducing the number of acute attacks, annual incidence rate, and severity, time to first exacerbation)	Unreported	EMA guideline 2012 [544]
	Patient questionnaire or diary card	Unreported	EMA guideline 2012 [544]
Assessment of the risk of disease exacerbation	number of hospitalizations	Perspective cohort study	GOLD 2021 [545], European Expert Consensus 2021 [552], Swiss Guideline 2018 [547], Chinese Expert Consensus 2017 [553]
	Frequency of exacerbation	Delphi survey	European Expert Consensus 2021 [552], Chinese Guideline 2021 [550]
	Alpha-1 antitrypsin	Perspective cohort study, cross-sectional study	Swiss Guideline 2018 [547]
	The use of rescue medications (such as β2 agonists, reliever inhalers), the frequency of daytime and nighttime rescue medication usage as well as the number of puffs used each time	Unreported	EMA guideline 2012 [544]
Comorbidity assessment	Lung cancer, skeletal muscle dysfunction, metabolic syndrome, cardiovascular disease, osteoporosis, anxiety disorder, and depression	Unreported	GOLD 2021 [545], Swiss Guideline 2018 [547]
Assessment of exercise capacity	6-minute walk test (6-minute walking distance)	Perspective cohort study, systematic review, Delphi survey	GOLD 2021 [545], European Expert Consensus 2021 [552], EMA guideline 2012 [544]
Assessment of the treatment of the disease			
Treatment for stable COPD	The severity assessment tool for chronic obstructive pulmonary disease (COPD)	Unreported	GOLD 2021 [545], European Expert Consensus 2021 [552]
Oxygen therapy	Blood oxygen measurement and arterial blood gas analysis	Perspective cohort study, cross-sectional study	GOLD 2021 [545]
Use of antibiotics	High-sensitivity C-reactive protein (hs-CRP) and procalcitonin	Systematic review, Delphi survey	GOLD 2021 [545], European Expert Consensus 2021 [552]
Use of corticosteroids	Eosinophils	Cohort study	GOLD 2021 [545]
Assessment of lung volume reduction surgery	CT scan of the lungs	RCT	GOLD 2021 [545]
Safety	Overall mortality, BODE index, cardiovascular adverse reactions (myocardial infarction, angina pectoris, hypertension, atrial fibrillation, heart failure, cardiovascular death, prolonged QT interval, systemic embolism, stroke, etc.) when long-acting β_2_ agonists are used in combination with anticholinergic drugs, incidence of pneumonia or lower respiratory tract infections	Unreported	EMA guideline 2012 [544]
	Exposure level and long-term safety data (Reliable prospective safety data from at least 1 year of treatment)	Unreported	EMA guideline 2012 [544]

Note: Guidelines/consensus with a gray background indicating an AGREE II evaluation of C.

The greened-out items represent domains and outcomes suggested by COS study, regulatory guidance documents, guidelines and expert consensus, and RCTs.

RCT: Randomized controlled trial.

HRQoL: Health-related quality of life.

SF-36: 36-item short form health survey.

EADL: Extended activities of daily living.

CT: Computed tomography.

BODE: Body-mass index, airflow obstruction, dyspnea, and exercise capacity.

### Potential efficacy outcomes of traditional Chinese medicine injection in AECOPD intervention

We used the above core outcomes to conduct a network meta-analysis and explore the potential efficacy outcomes of Tan-Re-Qing injection for AECOPD. It is important to note that in network meta-analysis, we did not assess treatment success/failure. This is because in the included RCT studies, all outcomes on treatment success/failure were customized composite outcomes defined based on the overall improvement in clinical symptoms or signs which made the summary of data impossible.

Under the comparison of CHI plus CIs versus CIs, we discovered that Tan-Re-Qing injection plus CIs (TanRQ_CI) showed the highest probability of being associated with a higher forced expiratory volume in one second predicted (FEV_1_%). The mean difference (MD) was 8.47 (95%CI 5.96 to 10.93), indicating a statistically significant improvement compared to CIs. The surface under the cumulative ranking curve (SUCRA) value (a ranking method) for this outcome was 75.65%, suggesting a high likelihood of superiority. Similar findings were observed for the forced vital capacity (FVC) and the forced expired volume in one second to forced vital capacity ratio (FEV_1_/FVC), where TanRQ_CI was associated with higher values compared to CIs, with MDs of 0.34 (95%CI 0.23 to 0.45) and 6.15 (95%CI 4.8 to 7.53), respectively.

In terms of arterial blood gas analysis, TanRQ_CI was associated with lower partial pressure of carbon dioxide in arterial blood (PaCO_2_) and higher oxygen saturation in arterial blood (SaO_2_) compared to CIs, with MDs of −7.26 (95%CI −8.62 to −5.91) and 6.26 (95%CI 4.02 to 8.59), respectively.

In terms of inflammatory markers, TanRQ_CI was also associated with lower C-reaction protein (CRP) (MD −7.13, 95%CI −10.13 to −4.17), lower White blood cell count (WBCC) (MD −0.86, 95%CI −1.44 to −0.29) compared to CIs, lower Procalcitonin (PCT) (MD −0.5, 95%CI −0.86 to −0.14), lower interleukin-6 (IL-6) (MD −9.11, 95%CI −13.04 to −5.08), and lower interleukin-8 (IL-8) (MD −6.56, 95%CI −9.05 to −4.1).

When comparing CHI plus CIs versus placebo plus CIs, TanRQ_CI was associated with a lower IL-8 (MD −5.96, 95%CI −11.46 to −0.46), based on one trial with 58 participants.

No statistically significant differences were found in other outcomes, such as Proportion of participants with one or more adverse events (PAE), FEV_1_, FVC, and IL-8, when comparing Tan-Re-Qing injection to CIs.

Please refer to Figures 3–14 and Table 9 for a visual representation of the findings.

**Figure 3.**
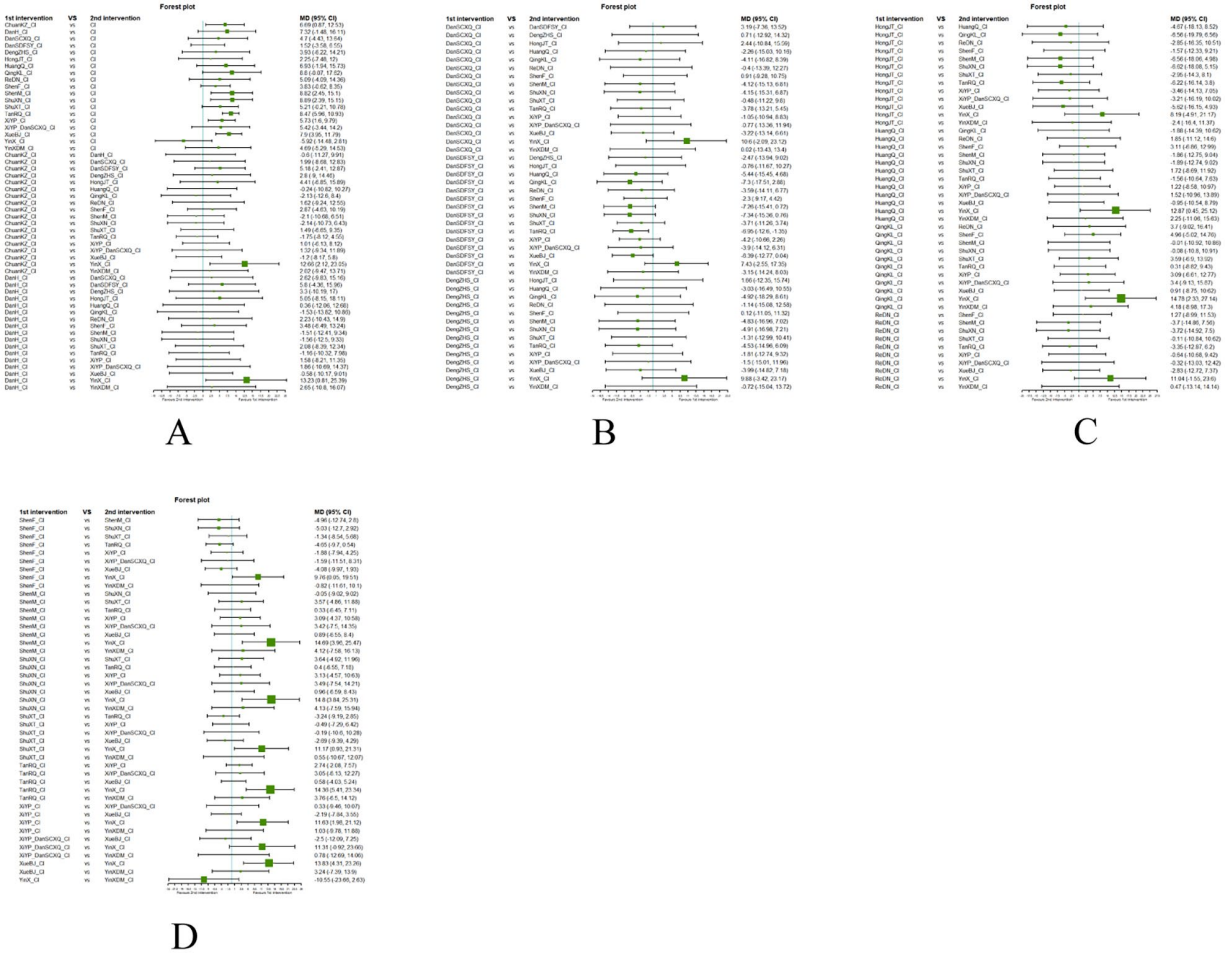
Forest plot of FEV1% under the comparison of CHIs plus CIs versus CLs. Note: We divided the forest plot of FEV1% into four parts which are subplots A to D of Figure 3.

**Figure 4.**
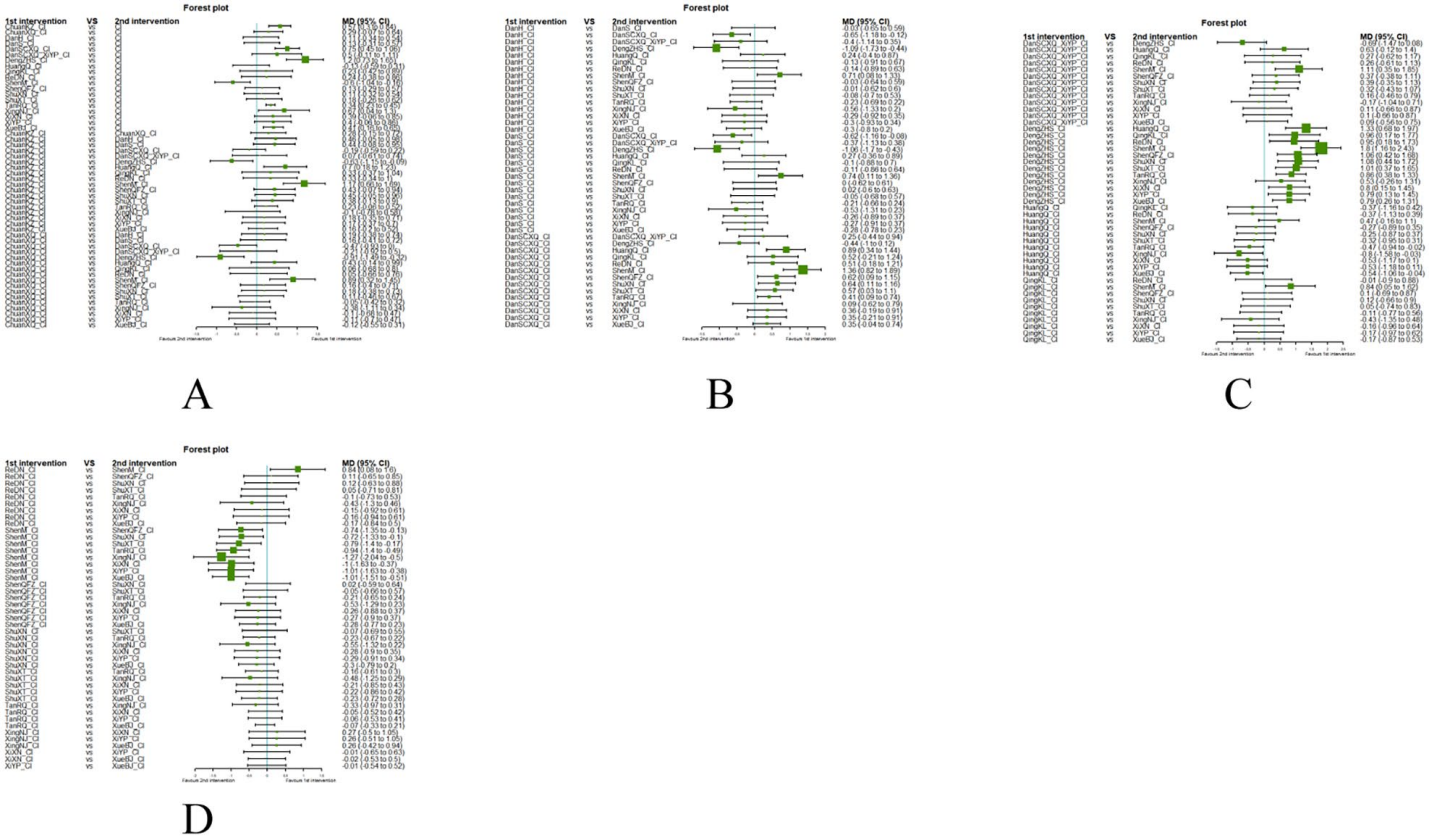
Forest plot of FVC under the comparison of CHIs plus CLs versus CLs. Note: We divided the forest plot of FVC into four parts which are subplots A to D of Figure 4.

**Figure 5.**
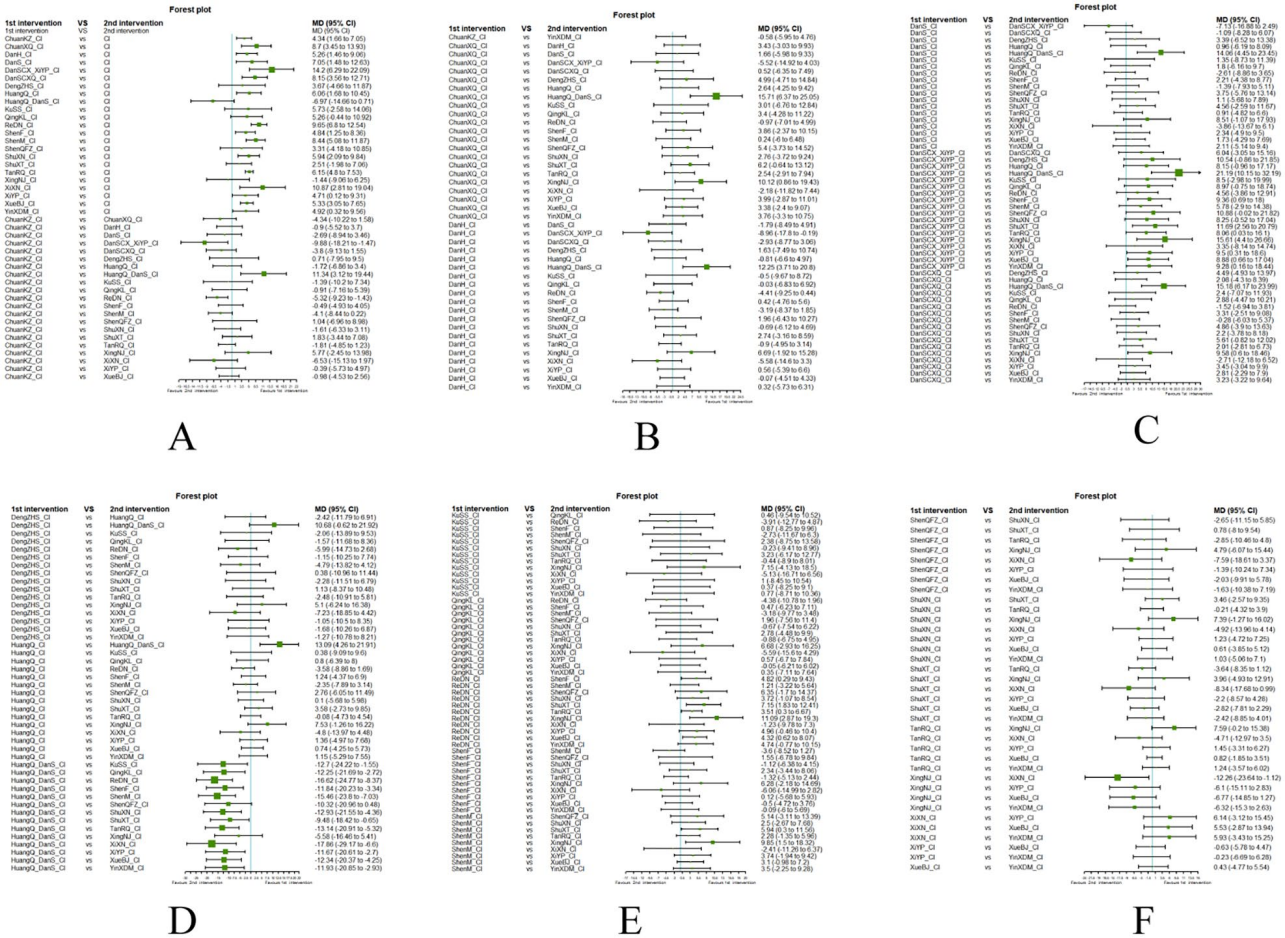
Forest plot of FEVI/FVC under the comparison of CHIs plus CIs versus CLs. Note: We divided the forest plot of FEVI/FVC into six parts which are subplots A to F of Figure 5.

**Figure 6.**
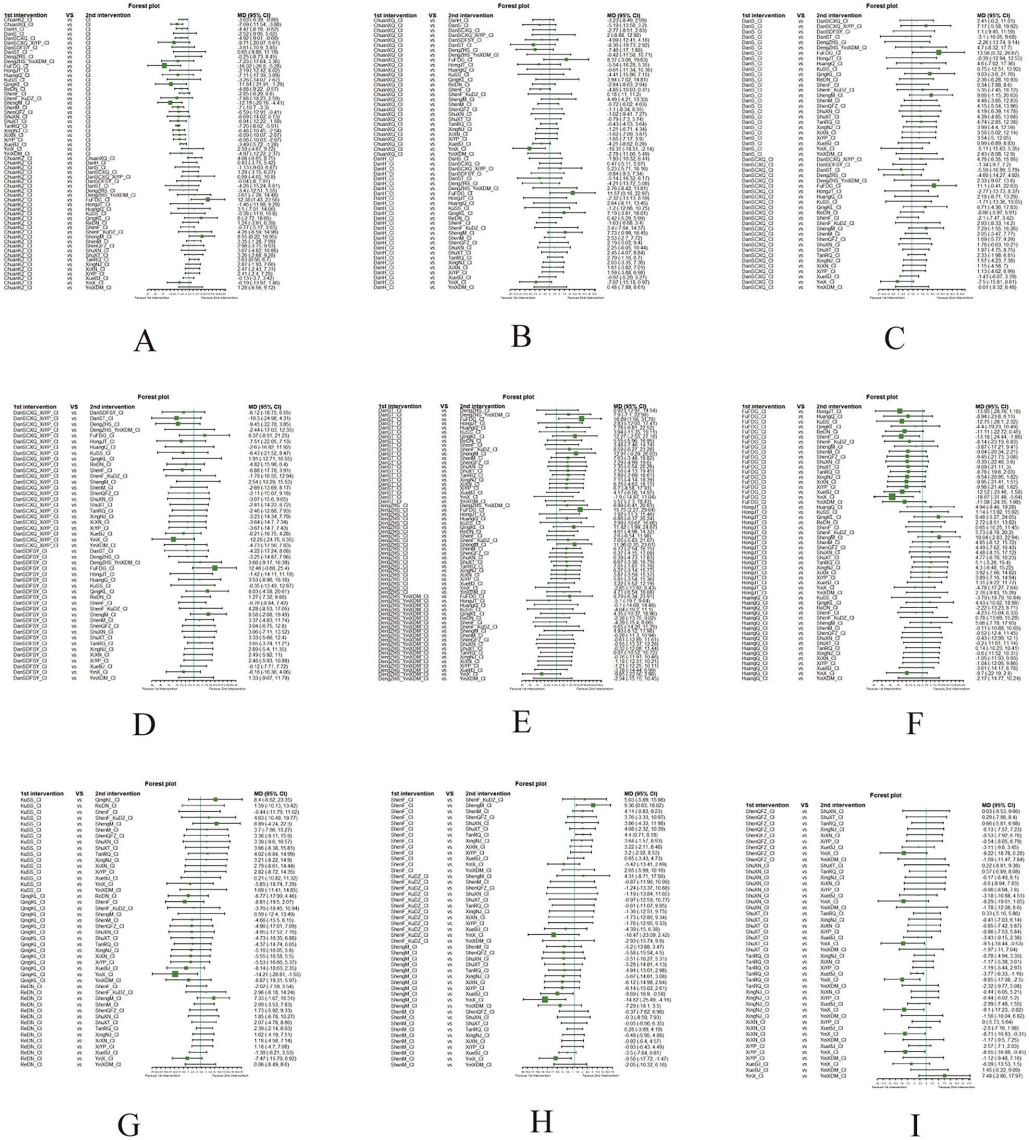
Forest plot of PaCO, under the comparison of CHIs plus CIs versus CIs. Note: We divided the forest plot of PaCO, into nine parts which are subplots A to I of Figure 6.

**Figure 7.**
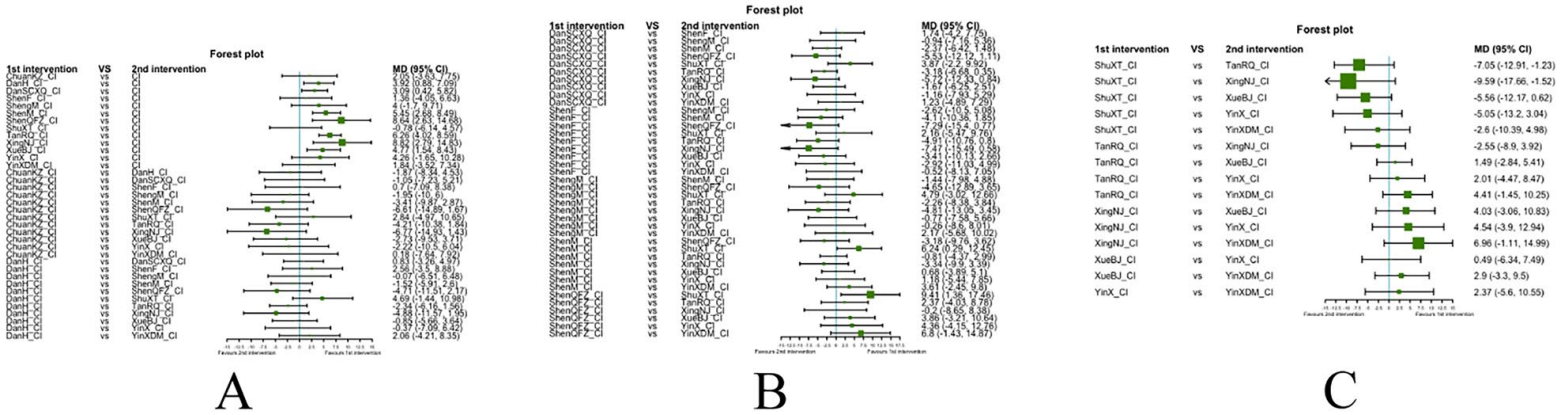
Forest plot of SaO, under the comparison of CHIs plus CIs versus CI. Note: We divided the forest plot of SaO, into three parts which are subplots A to C of Figure 7.

**Figure 8.**
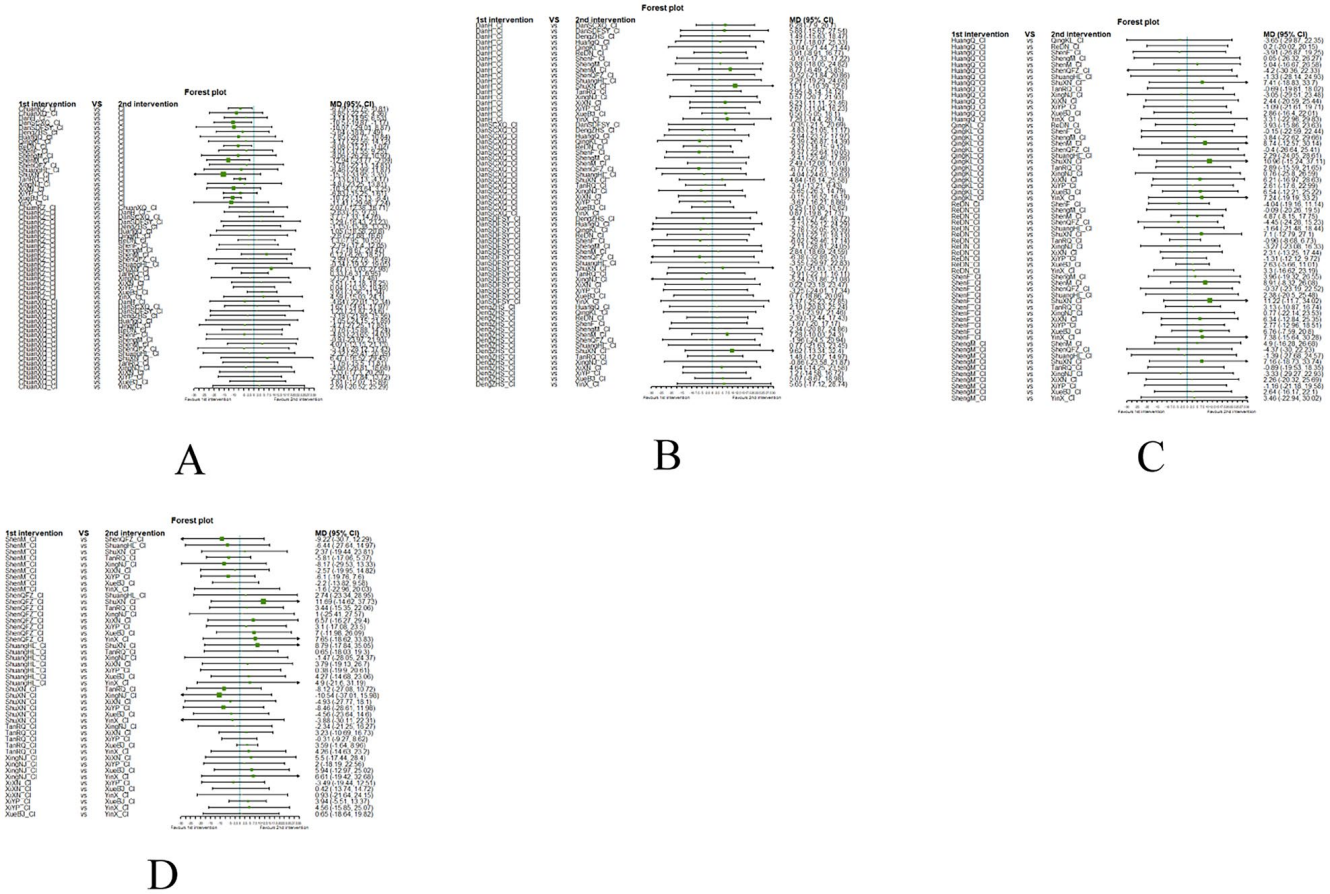
Forest plot of CRP under the comparison of CHIs plus CIs versus CLs. Note: We divided the forest plot of CRP into four parts which are subplots A to D of Figure 8.

**Figure 9.**
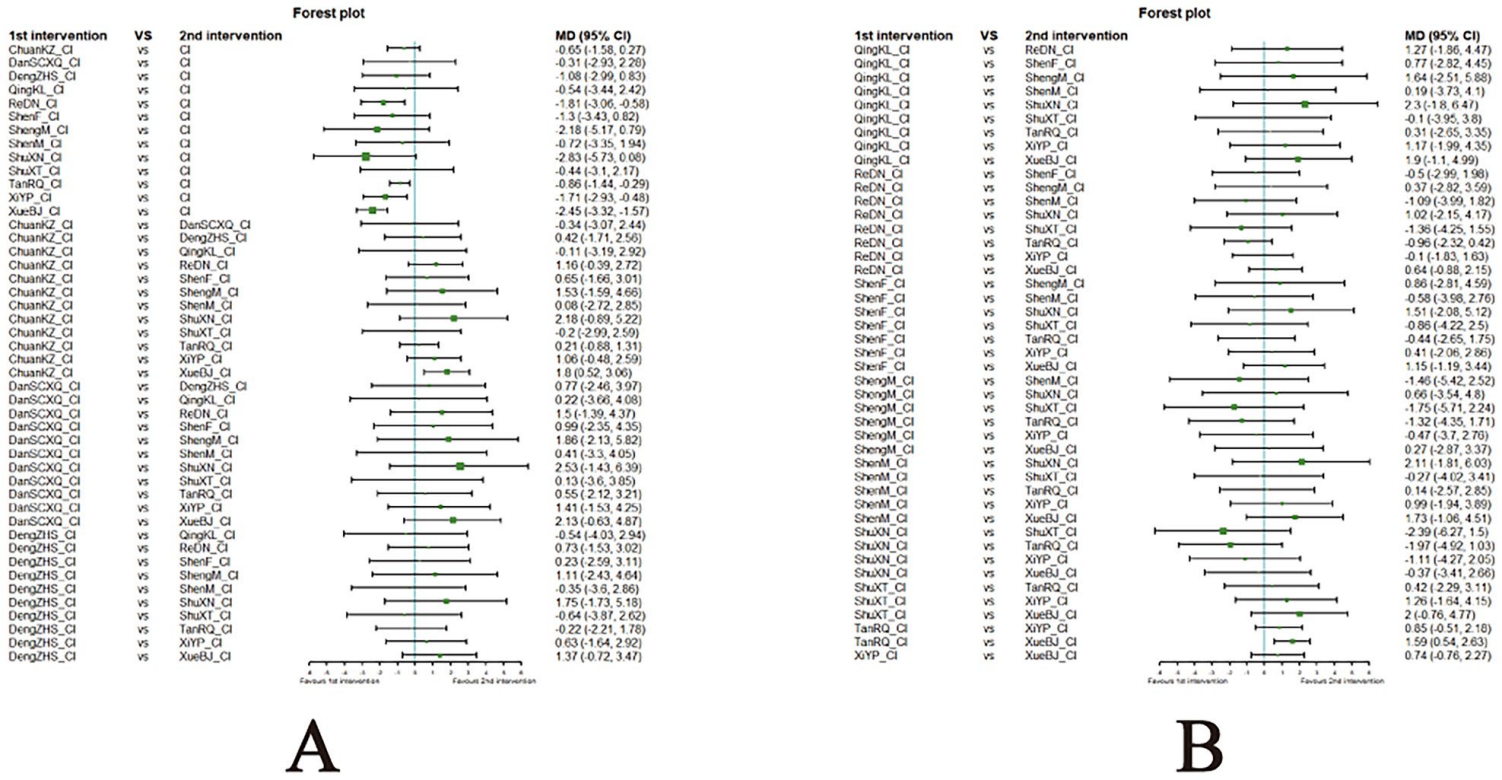
Forest plot of WBCC under the comparison of CHIs plus CIs versus CLs. Note: We divided the forest plot of WBCC into two parts which are subplots A to B of Figure 9.

**Figure 10.**
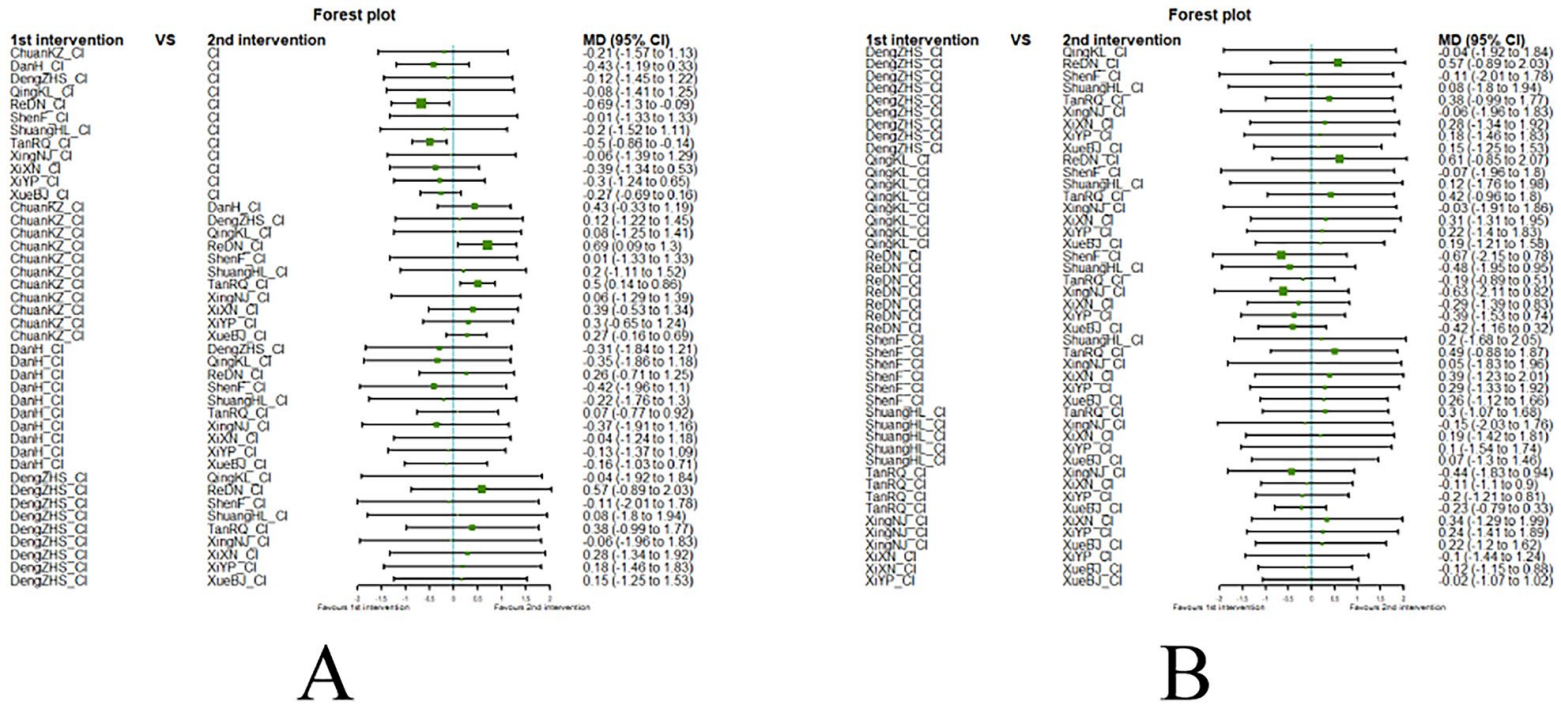
Forest plot of PCT under the comparison of CHIs plus CIs versus CLs. Note: We divided the forest plot of PCT into two parts which are subplots A to B of Figure 10.

**Figure 11.**
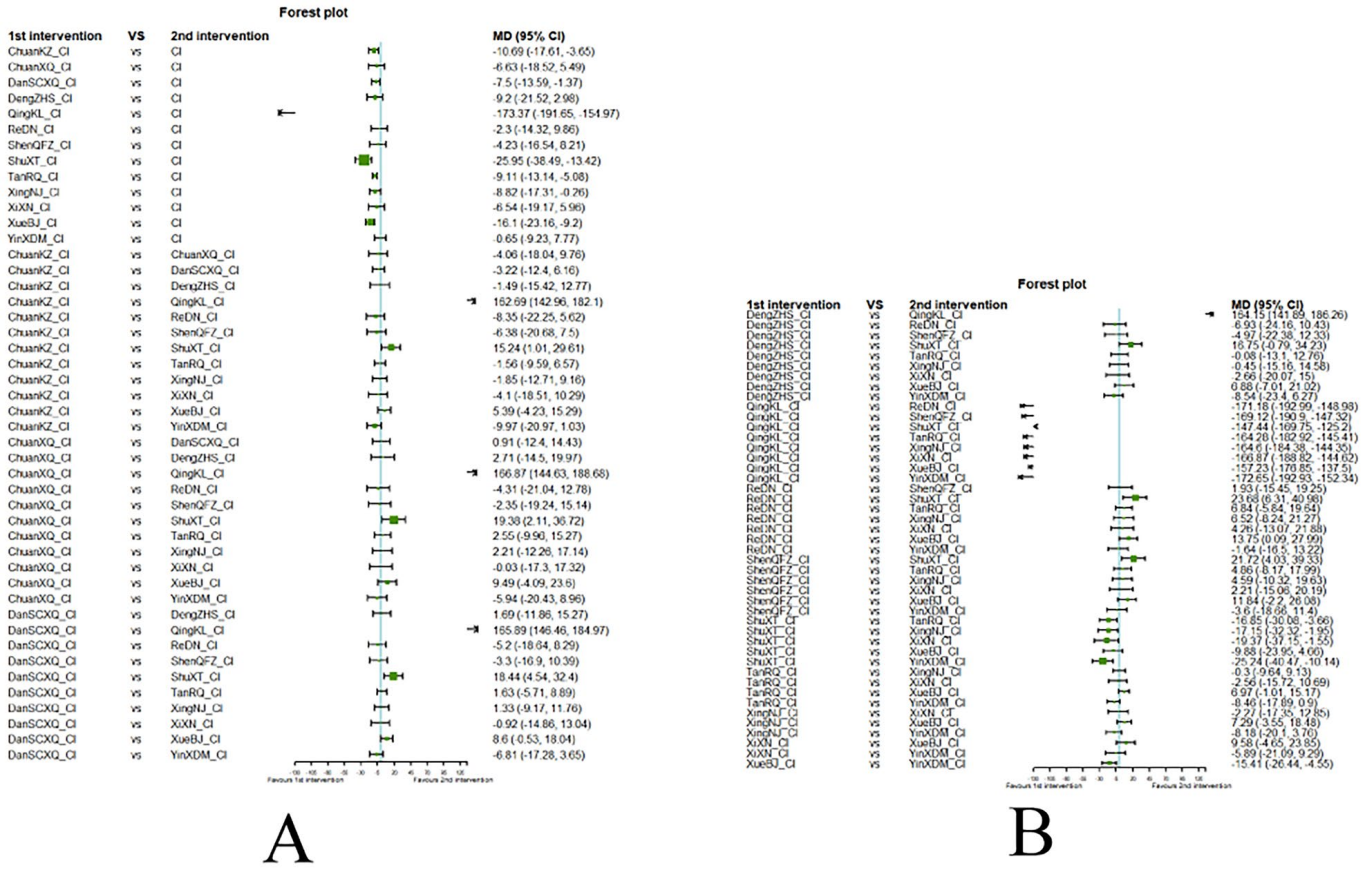
Forest plot of IL-6 under the comparison of CHIs plus CIs versus CLs. Note: We divided the forest plot of IL-6 into two parts which are subplots A to B of Figure 11.

**Figure 12.**
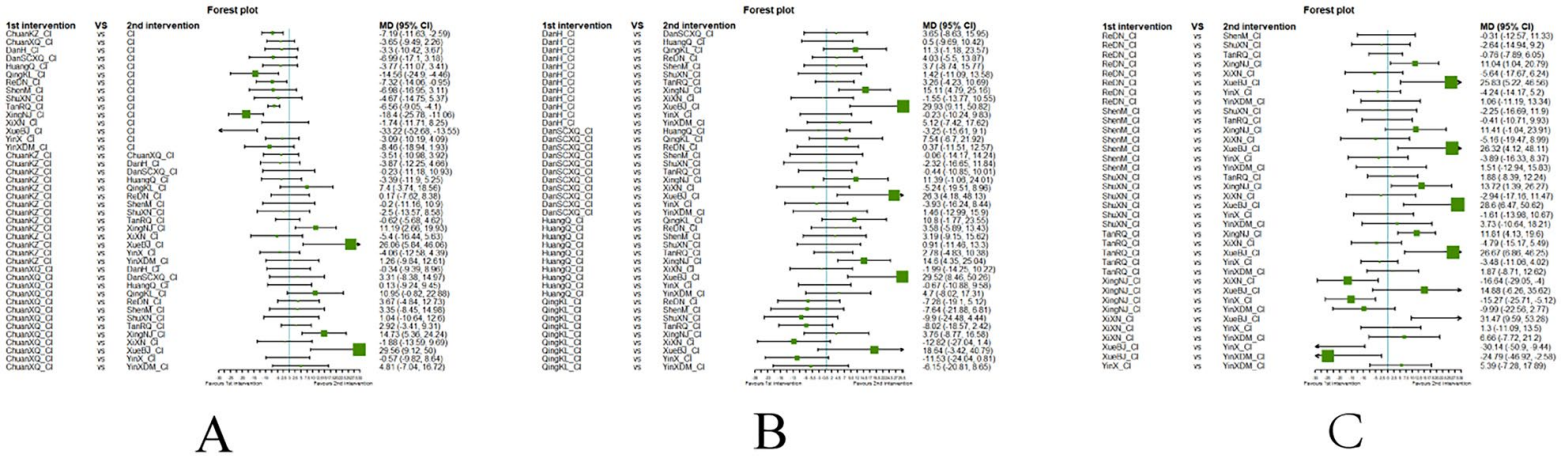
Forest plot of IL-8 under the comparison of CHIs plus CIs versus CIs. Note: We divided the forest plot of IL-8 into three parts which are subplots A to C of Figure 12.

**Figure 13.**
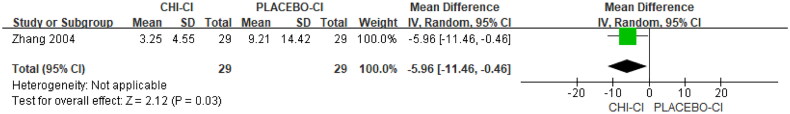
Forest plot of IL-8 under the comparison of CHIs plus CIs versus placebo plus CIs.

**Figure 14.**
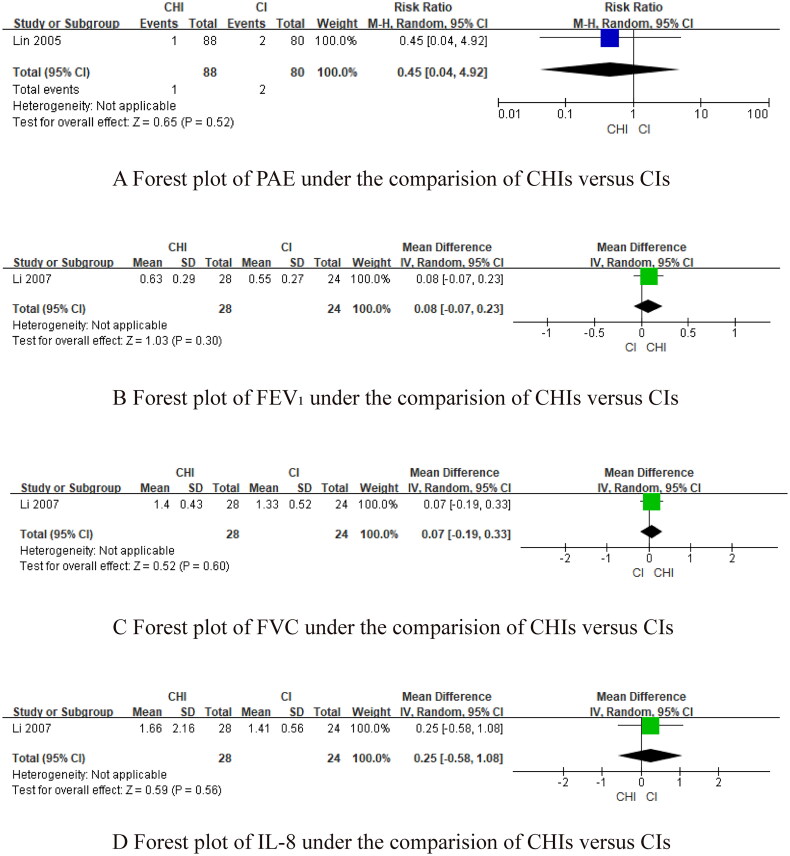
Forest plot of PAE, FEV, FVC and IL-8 under the comparison of CHIs versus CIs.

**Table 9.** NMA results of Tan-Re-Qing injection.

The type of comparisons	The type of outcome measures	Outcomes	MD/RR	95%CI	SUCRA	Rank
Chinese herbal injection plus CIs versus CIs	Pulmonary function test	FEV_1_%	MD: 8.47	(5.96 to 10.93)	75.65%	1
		FVC	MD: 0.34	(0.23 to 0.45)	55.52%	9
		FEV_1_/FVC	MD: 6.15	(4.8 to 7.53)	56.96%	8
	Blood gas analysis	PaCO_2_	MD: −7.26	(−8.62 to −5.91)	67.98%	6
		SaO_2_	MD: 6.26	(4.02 to 8.59)	77.39%	3
	Inflammatory markers	CRP	MD: −7.13	(−10.13 to −4.17)	47.04%	12
		WBCC	MD: −0.86	(−1.44 to −0.29)	40.86%	8
		PCT	MD: −0.5	(−0.86 to −0.14)	68.24%	2
		IL-6	MD: −9.11	(−13.14 to −5.08)	53.95%	5
		IL-8	MD: −6.56	(−9.05 to −4.1)	52.53%	7
Chinese herbal injection plus CIs versus placebo plus CIs	Inflammatory markers	IL-8	MD: −5.96	(−11.46 to −0.46)	–	–
Chinese herbal injection versus CIs	Pulmonary function test	FEV_1_	MD: 0.08	(−0.07 to 0.23)	–	–
		FVC	MD: 0.07	(−0.19 to 0.33)	–	–
	Inflammatory markers	IL-8	MD: −0.25	(−0.58 to 1.08)	–	–
	Proportion of participants with one or more adverse events	PAE	RR: 0.45	(0.04 to 4.92)	–	–

In light of the literature research conducted so far and in alignment with the objectives of our clinical study, our project team has engaged in productive discussions to establish the ‘Initial List of Outcomes for RCT of Tan-Re-Qing injection for AECOPD’. For further reference and details, please consult Supplementary Material 1.

### Consensus-based outcomes selection

#### Focus group interview recommendations for outcome measure selection

**Figure 15.**
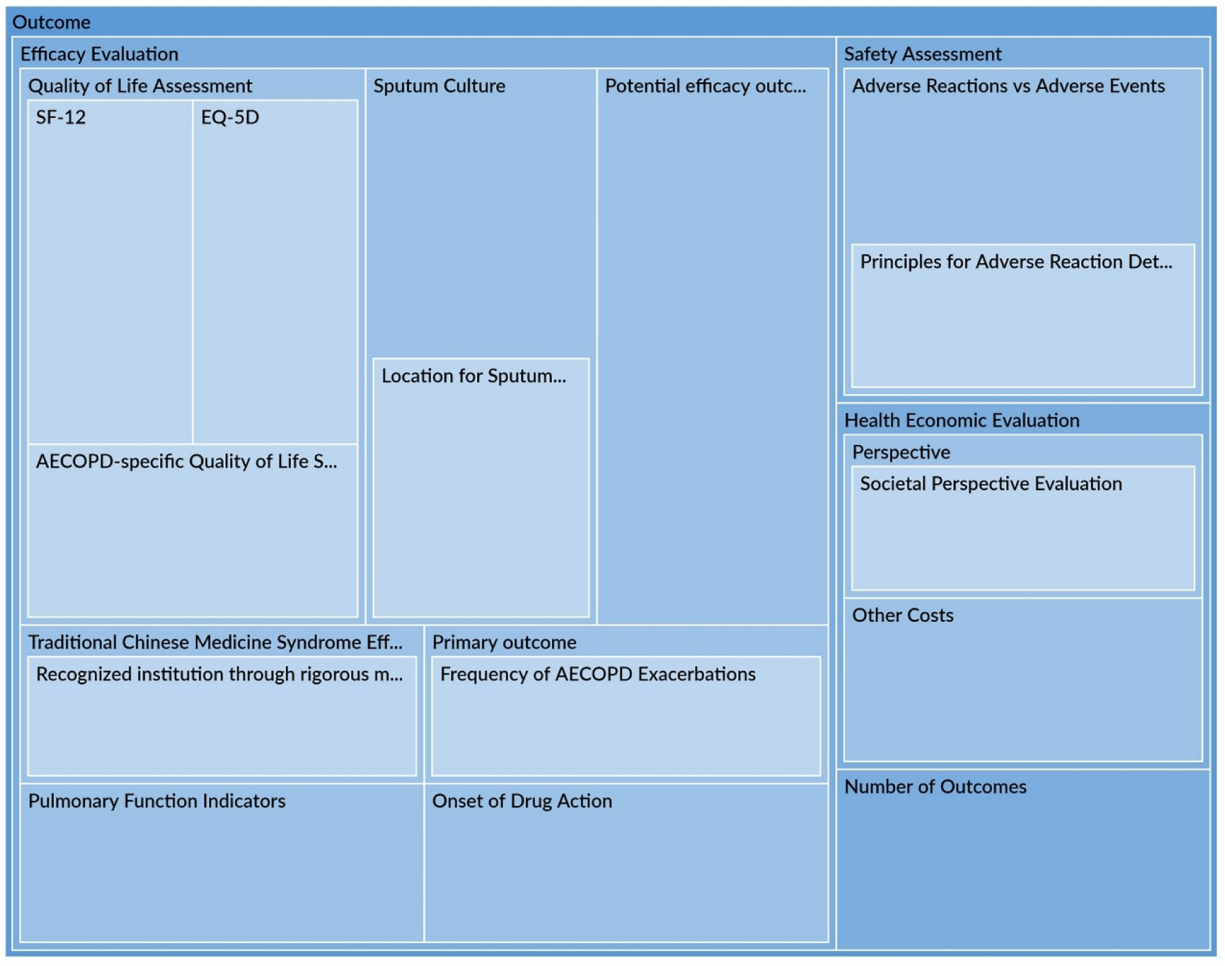
Hierarchical structure diagram of focus group interview topics.

The focus group interview was conducted online using Tencent Meeting on May 4, 2023. There were no refusals or dropouts among the participants. We presented the selected outcomes based on previous literature research (see Supplementary Material 1) to the members of the focus group and sought their opinions and recommendations regarding the selection of outcomes. A total of 53 codes were used to code the interview data. The main theme was outcomes, which comprised sub-themes such as efficacy evaluation, health economic evaluation, safety evaluation, and the number of outcomes. These themes were derived from the collected data. The hierarchical structure diagram of focus group interview topics are shown in Figure 15.

Based on the feedback received from the group members, we have made the following recommendations to further enhance efficacy evaluation, building upon the initial list of outcomes:

1. Quality of Life Assessment: It is recommended to use SF-12, EQ-5D, or the COPD-specific quality of life questionnaire (CAT) to assess the quality of life.
2. Focus on AECOPD Frequency and Lung Function: Pay close attention to the frequency of AECOPD and monitor lung function.
3. Incorporate Longitudinal Data Analysis Methods: Utilize longitudinal data analysis methods to analyze the time it takes for the drug to show its effectiveness.
4. Further Clarify Clinical Efficacy Advantages: Provide more clarity on the clinical efficacy advantages of the Tan-Re-Qing injection.
5. Consistent Sputum Culture Testing: Whenever possible, conduct sputum culture testing in the same unit to ensure consistency.
6. Traditional Chinese Medicine Efficacy Evaluation: For evaluating the efficacy of traditional Chinese medicine, it is recommended to use a well-developed traditional Chinese medicine syndrome efficacy evaluation scale.
7. Health Economic Evaluation: Position the perspective of health economic evaluation at a societal level and consider cost savings related to other aspects.
8. Safety Evaluation: Emphasize the assessment of adverse events and reactions and clearly define the principles for determining adverse reactions in the study protocol. Additionally, streamline the number of outcomes to focus on the most relevant ones.

Based on the literature analysis and the results of the focus group interviews, the project team has updated the list of selected outcomes and formulated the ‘Questionnaire for the Selection of Outcomes for the Clinical Evaluation of Tan-Re-Qing Injection Combined with Conventional Interventions for Acute Exacerbation of Chronic Obstructive Pulmonary Disease (AECOPD)’ (including 43 outcomes, see Supplementary Material 2).

#### Delphi survey

##### Expert enthusiasm

The enthusiasm of experts is represented by the questionnaire response rate.

In this survey, a total of 15 questionnaires were distributed, and all 15 were collected, resulting in a 100% response rate. The experts displayed a high level of enthusiasm. Please see Table 10 for the basic information of experts.

**Table 10.** Basic information of experts.

Item	Content	Number of experts
Gender	Male	8
	Female	7
Education	Master degree	2
	Doctorate	13
Title	Chief physician	3
	Deputy chief physician	3
	Researcher	6
	Associate researcher	1
	Professor	2
Years of work experience	5–10 years (including 10 years)	1
	10–20 years	11
	More than 20 years (including 20 years)	3
Field of expertise	Clinical research	6
	Clinical epidemiology research	3
	Evidence-based medicine research	5
	Others	1

##### Expert authority

According to the analysis, the questionnaire demonstrates a high level of expert authority with Ca of 0.92 (refer to Table 11). Additionally, Cs was measured at 0.83 (refer to Table 12), indicating a significant level of expertise in the research field. Moreover, Cr was calculated at 0.875, confirming that the research findings are relatively reliable.

**Table 11.** Table of expert assessment criteria.

Item	High	Medium	Low
Theoretical analysis	7	8	0
Practical experience	11	3	1
Relevant literature from domestic and international sources	9	6	0
Tuition	0	4	11

**Table 12.** Levels of expert familiarity.

Item	Frequency
Very familiar	5
Quite familiar	7
Moderately familiar	3
Not very familiar	0
Unfamiliar	0

#### Degree of concentration and coordination of expert opinions

##### Degree of concentration of expert opinions

According to the results of this study, the mean scores of all outcomes are greater than 4.0, with expert agreement being at least 60%. This indicates that the opinions of the experts involved in this research are relatively concentrated. Please refer to Table 13.

**Table 13.** Table of expert consensus agreement and coefficient of variation.

Outcomes	Importance	Frequency	Coefficient of agreement for importance(%)	Coefficient of agreement for selection(%)	Coefficient of variation	Mean score
Outcomes for efficacy evaluation						
The treatment failure rate	Important and critical	15	100	100	0.09	8.40
	Important but not critical	0	0			
	Unimportant	0	0			
Hospital readmission rate	Important and critical	12	80	100	0.15	7.27
	Important but not critical	3	20			
	Unimportant	0	0			
CAT score (The COPD Assessment Test)	Important and critical	12	80	93.33	0.19	6.80
	Important but not critical	2	13.33			
	Unimportant	1	6.67			
Score of the Acute Exacerbation of Chronic Obstructive Pulmonary Disease Symptoms and Treatment Efficacy Scale (AECOPD-STES)	Important and critical	12	80	100	0.15	7.33
	Important but not critical	3	20			
	Unimportant	0	0			
Length of hospital stay	Important and critical	11	73.33	100	0.18	7.20
	Important but not critical	4	26.67			
	Unimportant	0	0			
Proportion of patients requiring ICU treatment	Important and critical	11	73.33	100	0.17	7.27
	Important but not critical	4	26.67			
	Unimportant	0	0			
Frequency of AECOPD	Important and critical	10	66.67	100	0.2	7.20
	Important but not critical	5	33.33			
	Unimportant	0	0			
Proportion of patients receiving invasive mechanical ventilation	Important and critical	10	66.67	100	0.19	6.93
	Important but not critical	5	33.33			
	Unimportant	0	0			
Proportion of patients with AECOPD-related complications	Important and critical	10	66.67	100	0.19	7.07
	Important but not critical	5	33.33			
	Unimportant	0	0			
Duration of recurrent AECOPD	Important and critical	9	60	93.33	0.26	6.33
	Important but not critical	5	33.33			
	Unimportant	1	6.67			
Duration of fever	Important and critical	9	60	93.33	0.31	6.60
	Important but not critical	5	33.33			
	Unimportant	1	6.67			
HRQOL-5 scale score	Important and critical	9	60	93.33	0.24	6.33
	Important but not critical	5	33.33			
	Unimportant	1	6.67			
Score of Chinese version of EQ-5D Quality of Life Scale	Important and critical	8	53.33	93.33	0.26	6.40
	Important but not critical	6	40			
	Unimportant	1	6.67			
Forced expiratory volume in one second predicted(FEV_1_%)	Important and critical	8	53.33	100	0.22	6.53
	Important but not critical	7	46.67			
	Unimportant					
Forced expired volume in one second to forced vital capacity ratio (FEV1/FVC)	Important and critical	7	46.67	93.34	0.25	6.40
	Important but not critical	7	46.67			
	Unimportant	1	6.67			
Forced vital capacity (FVC)	Important and critical	6	40	100	0.19	6.20
	Important but not critical	9	60			
	Unimportant	0	0			
Duration of ICU treatment	Important and critical	5	33.33	93.33	0.25	6.00
	Important but not critical	9	60			
	Unimportant	1	6.67			
White blood cell count	Important and critical	5	33.33	73.33	0.38	5.07
	Important but not critical	6	40			
	Unimportant	4	26.67			
C-reactive protein level	Important and critical	5	33.33	80	0.41	5.27
	Important but not critical	7	46.67			
	Unimportant	3	20			
Interleukin-6 (IL-6) level	Important and critical	3	20	73.33	0.37	4.80
	Important but not critical	8	53.33			
	Unimportant	4	26.67			
Procalcitonin (PCT) level	Important and critical	3	20	73.33	0.39	4.93
	Important but not critical	8	53.33			
	Unimportant	4	26.67			
Prevalence of drug-resistant bacteria	Important and critical	3	20	73.33	0.37	5.13
	Important but not critical	8	53.33			
	Unimportant	4	26.67			
PaO_2_ level	Important and critical	2	13.33	86.66	0.26	5.20
	Important but not critical	11	73.33			
	Unimportant	2	13.33			
PaCO_2_ level	Important and critical	2	13.33	86.66	0.29	5.47
	Important but not critical	11	73.33			
	Unimportant	2	13.33			
Number of drug-resistant bacteria	Important and critical	2	13.33	60	0.45	4.60
	Important but not critical	7	46.67			
	Unimportant	6	40			
Interleukin-8 (IL-8) level	Important and critical	2	13.33	60	0.38	4.40
	Important but not critical	7	46.67			
	Unimportant	6	40			
Outcomes for safety evaluation						
Proportion of patients with serious adverse events	Important and critical	15	100	100	0.11	8.20
	Important but not critical	0	0			
	Unimportant	0	0			
Proportion of patients with non-serious adverse events	Important and critical	11	73.33	93.33	0.22	6.87
	Important but not critical	3	20			
	Unimportant	1	6.67			
Proportion of patients with abnormal renal function	Important and critical	11	73.33	93.33	0.26	6.93
	Important but not critical	3	20			
	Unimportant	1	6.67			
Proportion of patients with abnormal liver function	Important and critical	10	66.67	93.34	0.26	6.87
	Important but not critical	4	26.67			
	Unimportant	1	6.67			
Proportion of patients with pneumonia progression	Important and critical	9	60	100	0.24	6.60
	Important but not critical	6	40			
	Unimportant	0				
Proportion of patients with abnormal urinalysis	Important and critical	7	46.67	80	0.43	5.40
	Important but not critical	5	33.33			
	Unimportant	3	20			
Proportion of patients with abnormal coagulation function	Important and critical	6	40	80	0.42	5.60
	Important but not critical	6	40			
	Unimportant	3	20			
Duration of antibiotic use	Important and critical	4	26.67	93.34	0.31	5.87
	Important but not critical	10	66.67			
	Unimportant	1	6.67			
Transition time from intravenous to oral antibiotics	Important and critical	4	26.67	86.67	0.36	5.33
	Important but not critical	9	60			
	Unimportant	2	13.33			
Cumulative dose of antibiotics	Important and critical	4	26.67	86.67	0.35	5.33
	Important but not critical	9	60			
	Unimportant	2	13.33			
Cumulative dose of corticosteroids	Important and critical	4	26.67	93.34	0.3	5.73
	Important but not critical	10	66.67			
	Unimportant	1	6.67			
Duration of corticosteroid use	Important and critical	4	26.67	93.34	0.3	5.73
	Important but not critical	10	66.67			
	Unimportant	1	6.67			
Transition time from broad-spectrum to narrow-spectrum antibiotics	Important and critical	3	20	93.33	0.33	5.33
	Important but not critical	11	73.33			
	Unimportant	1	6.67			
Outcomes for healthcare economics evaluation						
Cost-effectiveness ratio (CER)	Important and critical	0	0	93.33	0.19	7.40
	Important and critical	14	93.33			
	Unimportant	1	6.67			
Incremental cost-effectiveness ratio	Important and critical	14	93.33	100	0.11	7.60
	Important but not critical	1	6.67			
	Unimportant	0				
Effectiveness outcomes	Important and critical	13	86.67	100	0.14	7.33
	Important but not critical	2	13.33			
	Unimportant	0				
Quality-adjusted life years (QALY)	Important and critical	12	80	100	0.17	7.13
	Important but not critical	3	20			
	Unimportant	0				
Health utility value	Important and critical	9	60	93.33	0.29	6.33
	Important but not critical	5	33.33			
	Unimportant	1	6.67			

Note: The outcome measures are ordered based on the level of agreement regarding the importance and criticality of the outcome.

PaO_2_: Partial pressure of oxygen in arterial blood.

PaCO_2_: Partial pressure of carbon dioxide in arterial blood.

The pinked-out items represent outcome measures suggested for exclusion by the Delphi questionnaire survey.

The red-out items represent finally excluded outcome measures.

##### Degree of coordination of expert opinions

This study identified outcomes with a CV of 0.3 or higher as indicators reflecting significant expert disagreement. The coefficients of expert agreement and CV values for each indicator can be found in Table 13. WBCC, IL-6 level, PCT level, the prevalence of drug-resistant bacteria, number of drug-resistant bacteria, and IL-8 level had a coefficient of expert agreement less than 80% and a CV of 0.3 or higher. However, based on network meta-analysis, white blood cell count, IL-6 level, PCT level, and IL-8 Level were identified as potential efficacy outcomes for AECOPD intervention using Tan-Re-Qing injection and thus were retained. The indicators prevalence of drug-resistant bacteria and number of drug-resistant bacteria were excluded.

In total, 41 outcomes were selected for the RCT designation, with the treatment failure rate being the primary outcome. For more details, please refer to Table 13.

## Discussion

The methodology employed in this study aimed to comprehensively evaluate the selection and evaluation of outcomes for the clinical evaluation of Tan-Re-Qing injection in the treatment of AECOPD. The research design incorporated a literature review, quality assessment of included publications, expert opinions through a Delphi survey, and focus group recommendations. Based on the results, we have gained valuable insights.

Firstly, the literature analysis revealed a total of 513 publications, including regulatory guidance documents, guidelines, expert consensus, and randomized controlled trials (RCTs). These publications provided a comprehensive overview of the current landscape of outcomes used in AECOPD intervention studies. The inclusion of diverse publication types ensured a comprehensive understanding of the topic. Although WHO does not establish recommendations regarding clinical research, it does have definitions and fundamental opinions regarding essential medicines. The requirements for essential medicines also provide some reference value for the clinical efficacy evaluation of drugs, thus, we considered WHO requirements for outcome selection in this study. Based on the analysis of regulatory guidelines, we have initially determined that three dimensions should be considered when clinically evaluating Tan-Re-Qing injection for AECOPD: clinical efficacy, safety, and cost-effectiveness. When determining outcomes, it is necessary to clarify the definition and measurement methods and tools of the outcome measures. The development of primary outcome measures should be based on the most important objectives of clinical research. According to these guidelines, the trial outcomes should be clearly defined, and the primary outcomes should reflect the main clinical effects and be selected based on the primary objectives of the study. Secondary outcomes, on the other hand, are used to evaluate other effects of the drug and may or may not be related to the primary outcomes. It is also important to pre-specify the trial outcomes and their analysis plans in the study protocol.

To assess the quality of the included publications, a rigorous quality assessment was conducted. The assessment focused on various aspects, including the methodological quality of COS research, guidelines and expert consensus, and RCTs.

The included COS study exhibited good research quality, and its recommended COS should be given due consideration. Although two of the included guidelines were rated as C-grade, their recommendations should be carefully considered. However, it should be noted that the outcomes recommended by these two guidelines were also recommended in the other seven guidelines/consensus, which were rated as B-grade. Therefore, the outcomes can be included in the scope of consideration.

Although the quality of the included RCTs was relatively poor, this study focuses on the selection of outcome measures. The reported outcome measures in the RCTs can serve as alternative options in the pool of potential outcome measures for reference.

This assessment provided valuable insights into the strengths and weaknesses of the included publications, enabling a critical evaluation of their recommendations. Based on the summary, the following domains were recommended for evaluation by various guidelines and RCTs: death, treatment success/failure, future impact, health-related quality of life, and safety.

The following outcomes were recommended by both guidelines and RCTs: Death from any cause, levels of oxygen and carbon dioxide in the blood (arterial blood gases), CAT, the number of exacerbations, serious adverse events from treatments, and Lung function (FEV_1_, FVC, and FEV_1_/FVC).

We also included outcome measures reported in RCTs that accounted for more than 6% of the total into the pool of alternative outcome measures.

Additionally, we conducted a network meta-analysis to explore potential specific therapeutic efficacy outcomes for AECOPD intervention with Tanreqing Injection. If the chosen outcomes in clinical trials lack clear association with the disease states or treatment effects, it can diminish their clinical reliability and practicality, thus constraining their guidance for clinical practice and decision-making. Furthermore, inadequate sensitivity or specificity of outcomes to treatment interventions may result in overlooking genuine treatment effects or misinterpreting changes induced by other factors as treatment effects. Hence, by conducting a network meta-analysis, we assessed whether the selected outcome measures from the aforementioned steps demonstrate clear correlation with the disease states or treatment effects and possess sufficient sensitivity, thereby offering essential guidance for selecting outcomes in future clinical research within the same domain. And outcomes such as inflammatory markers like WBCC, GR%, IL-6, and IL-8 were identified.

The focus group recommendations played a crucial role in updating the list of selected outcomes.

The focus group recommended using SF-12, EQ-5D, or the COPD-specific quality of life questionnaire (CAT) to assess quality of life. Emphasis was placed on evaluating AECOPD frequency and lung function. Furthermore, it was highlighted that clinical advantages of Tan-Re-Qing injection should be emphasized and explored, along with specific outcomes for evaluating the efficacy of traditional Chinese medicine interventions. The focus group discussions provided an opportunity for in-depth exploration and consideration of various perspectives, contributing to the robustness of the outcome measure selection process. Based on the focus group recommendations, a list of selected outcomes was updated and a questionnaire was formulated. This questionnaire included 43 outcomes, which were carefully selected based on the expert opinions and literature analysis.

Expert opinions were obtained through a Delphi survey. The high response rate and coefficients of expert authority, familiarity, and reliability indicated a significant level of expertise and reliability in the research. Additionally, the degree of concentration and coordination of expert opinions was assessed to determine the level of agreement among the experts. The results showed that the experts’ opinions in this research were relatively concentrated, indicating a high level of agreement on the selected outcomes.

Considering medicine such as Tan-Re-Qing injection for AECOPD can impact the quantity and other properties of sputum, currently no outcome evaluating mucus were included. Potential outcomes include Mucin 5 Subtype AC (MUC5AC) level, sputum rheology, CT imaging scan (to determine a mucus score), and cough/sputum specific questionnaire such as Sputum Color Chart can be considered.

The methodology employed in this study had several strengths. The comprehensive literature review encompassed a wide range of publication types, ensuring a thorough understanding of the topic. The quality assessment of included publications provided an objective evaluation of their methodological rigor. The focus group recommendations further enriched the selection process of outcomes. The incorporation of expert opinions through a Delphi survey ensured the involvement of key stakeholders and the consideration of diverse perspectives.

## Limitations

It is important to acknowledge the limitations of the methodology. The exclusion of potentially relevant studies and guidelines may have limited the scope of the literature review. The Delphi survey and focus group recommendations relied on a specific panel of experts, and their opinions may not fully represent the entire field and the perspectives of all relevant stakeholders.

## Conclusion

In conclusion, the methodology employed in this study provided a robust framework for selecting and evaluating outcomes for the clinical evaluation of Tan-Re-Qing injection in AECOPD. The combination of a comprehensive literature review, quality assessment of included publications, expert opinions, and focus group recommendations ensured a thorough and systematic approach to the research. The findings from this study contribute to the development of evidence-based practices in the field of AECOPD intervention studies. Future research should consider addressing the limitations identified in this study to further enhance the methodology.

## Supplementary Material

Supplemental Material

## Data Availability

The data that support the findings of this study are available on request from the corresponding author. The data are not publicly available due to their containing information that could compromise the privacy of research participants.
