# Supplementary material for: Methodology for the selection and evaluation of outcomes for Chinese herbal injection in acute exacerbation of chronic obstructive pulmonary disease (AECOPD): a comprehensive study
Source: Ann Med. 2024 Sep 10;56(1):2396567. doi: 10.1080/07853890.2024.2396567 (PMC11389646; doi:10.1080/07853890.2024.2396567)
Supplement: Supplemental Material [file IANN_A_2396567_SM3152.docx]

## Appendix 1 Initial List of outcomes for RCT of Tan-Re-Qing for AECOPD

### 1 Outcomes for Efficacy Evaluation

### Primary Outcome

### The treatment failure rate (%)

### Definition: defined as the combination of the following three endpoints from the start of treatment to the 12-week follow-up: (1) intensification treatment with systemic corticosteroids or mechanical ventilation due to respiratory reasons, (2) intensified hospitalization, including transfer to the intensive care unit (ICU) or readmission due to respiratory reasons, or (3) all-cause mortality. The occurrence of any of these endpoints is considered treatment failure.

**1.2 Secondary Outcomes**

1.2.1 Length of hospital stay

Definition: the number of days from the start of treatment to the patient’s discharge from the hospital.

1.2.2 Hospital readmission rate (%)

Definition: the proportion of patients who are readmitted due to COPD or related complications within a 24-week follow-up period after treatment discharge, in relation to the total number of patients in the group.

1.2.3 Duration of recurrent AECOPD (days)

Definition: the number of days from treatment discharge until the first occurrence of AECOPD within a 24-week follow-up period.

1.2.4 All-cause mortality (%)

Definition: the measurement of deaths from all causes.

1.2.5 Proportion of patients requiring ICU treatment (%)

Definition: the proportion of patients in a group who require transfer to the Intensive Care Unit (ICU) for treatment during their treatment period. This is measured by the number of patients who require ICU treatment divided by the total number of patients in the group.

1.2.6 Proportion of patients receiving invasive mechanical ventilation (%)

Definition: the proportion of patients in a treatment group who require invasive mechanical ventilation during their treatment period, expressed as a percentage.

1.2.7 Proportion of patients with AECOPD-related complications (%):

Definition: the proportion of patients in a treatment group who experience AECOPD-related complications during their treatment period.

1.2.8 CAT score

Definition: the score of COPD Assessment Test.

1.2.9 mMRC score

Definition: the score of mMRC (Modified Medical Research Council Dyspnea Scale).

1.2.10 EXACT-PRO score

Definition: EXACT-PRO is a daily electronic diary tool used to assess the frequency, severity, and duration of exacerbations in patients with chronic obstructive pulmonary disease (COPD). It is completed by the participants before going to sleep, reflecting on their symptoms throughout the day, and it allows for the collection of daily changes in the patient's condition. It consists of 14 items grouped into four symptom clusters: breathlessness, cough and sputum, chest symptoms, and additional attributes. The scores range from 0 to 100, with higher scores indicating more severe symptoms. Changes in the total score are used to define the onset and resolution of exacerbation events, as well as the severity of these events. This evaluation is conducted daily during hospitalization.

1.2.11 SF-36 score

Definition: the score of SF-36.

1.2.12 EQ-5D score

Definition: the score of Chinese version of EQ-5D Quality of Life Scale

1.2.13 Score of the Acute Exacerbation of Chronic Obstructive Pulmonary Disease Symptoms and Treatment Efficacy Scale (AECOPD-STES)

1.2.14 Complete blood count

1.2.15 C-Reactive Protein Concentration (mg/L)

Definition: the concentration of C-reactive protein in the plasma.

1.2.16 Results of arterial blood gas analysis

1.2.17 Prevalence of Drug-Resistant Bacteria (%)

Definition: in sputum culture and antibiotic susceptibility testing, the proportion of individuals with drug-resistant bacteria to the total number of individuals in that group.

1.2.18 Number of Drug-Resistant Bacteria (Count)

Definition: in sputum culture and antibiotic susceptibility testing, when drug-resistant bacteria are identified, the number of strains resistant to specific antibiotics is recorded and the count of resistant bacteria is determined.

1.2.19 Forced expiratory volume in one second predicted（FEV_1_%）

Definition: in pulmonary function testing, the FEV1 (forced expiratory volume in one second) as a percentage of the predicted maximum value.

1.2.20 Forced Vital Capacity (FVC) (L)

Definition: in pulmonary function testing, the total volume of air that a person can forcefully exhale during a complete respiratory cycle, starting from maximum inhalation to maximum exhalation under maximal effort.

1.2.21 FEV_1_/FVC Ratio (%)

Definition: the ratio of forced expiratory volume in one second (FEV1) to forced vital capacity (FVC) in pulmonary function testing.

1.2.22 Proportion of Patients with Pneumonia Progression (%)

Definition: Refers to the proportion of patients who experience pneumonia progression during the treatment period, relative to the total number of patients in the treatment group. Pneumonia progression is defined as the presence of new consolidations observed through chest CT imaging.

1.2.23 Procalcitonin (PCT) Level (ng/mL)

Definition: The concentration of procalcitonin in the blood.

**2 Outcomes for Safety Evaluation**

2.1 Duration of Antibiotic Use (days)

Definition: the amount of time required from the first administration of an antibiotic to the first discontinuation of antibiotic use.

2.2 Cumulative Dose of Antibiotics (mg)

Definition: The cumulative dose of antibiotics used during the patient’s treatment period.

2.3 Cumulative Dose of Corticosteroids (mg)

Definition: The cumulative dose of corticosteroids used during the patient’s treatment period.

2.4 Duration of Corticosteroid Use (days)

Definition: The cumulative duration of corticosteroid usage during the patient’s treatment period.

2.5 Proportion of Patients with Abnormal Urinalysis (%)

Definition: refers to the proportion of patients who have abnormal results in urinalysis during the treatment period, relative to the total number of patients in the treatment group. Urinalysis includes evaluations of appearance, pH level, specific gravity, protein, glucose, ketones, nitrites, occult blood, leukocyte esterase, cellular count and morphology, bacteria and fungi, as well as crystal and particle detection.

2.6 Proportion of Patients with Abnormal Urinalysis (%)

Definition: refers to the proportion of patients who have abnormal results in urinalysis during the treatment period, relative to the total number of patients in the treatment group. Urinalysis includes evaluations of appearance, pH level, specific gravity, protein, glucose, ketones, nitrites, occult blood, leukocyte esterase, cellular count and morphology, bacteria and fungi, as well as crystal and particle detection.

2.7 Proportion of Patients with Abnormal Coagulation Function

Definition: refers to the proportion of patients who have abnormal results in coagulation function tests during the treatment period, relative to the total number of patients in the treatment group. Coagulation function tests include prothrombin time (PT), activated partial thromboplastin time (APTT), thrombin time (TT), fibrinogen, D-dimer, platelet count, and platelet function.

2.8 Proportion of Patients with Abnormal Liver Function

Definition: refers to the proportion of patients who have abnormal results in liver function tests during the treatment period, relative to the total number of patients in the treatment group. Liver function tests include hepatic enzyme tests: aspartate aminotransferase (AST), alanine aminotransferase (ALT), alkaline phosphatase (ALP), and gamma-glutamyl transferase (GGT), as well as serum levels of total protein and albumin, and serum levels of bilirubin and direct bilirubin.

2.9 Proportion of Patients with Abnormal Renal Function

Definition: refers to the proportion of patients who have abnormal results in renal function tests during the treatment period, relative to the total number of patients in the treatment group. Renal function tests include measurements such as serum creatinine, blood urea nitrogen (BUN), urea/creatinine ratio, uric acid, and serum uric acid clearance. These tests are used to assess the kidney’s ability to filter waste products from the blood and maintain proper fluid and electrolyte balance in the body.

2.10 Proportion of Patients with Abnormal Stool Routine Examination (%)

Definition: refers to the proportion of patients who have abnormal results in stool routine examination during the treatment period, relative to the total number of patients in the treatment group. Stool routine examination includes. It involves the observation and analysis of stool samples for various parameters such as appearance, color, consistency, pH level, occult blood, and presence of parasites. Through this examination, healthcare professionals can gain insights into the functioning of the digestive system and detect any signs of infection, inflammation, bleeding, or other abnormalities.

2.11 Proportion of Patients with Abnormal Electrocardiogram (%)

Definition: refers to the proportion of patients who have abnormal results in Electrocardiogram.

2.12 The incidence of COPD-related complication (%)

2.13 Proportion of Patients with Serious Adverse Events (%)

Definition: refers to the proportion of patients who experience serious adverse events during the treatment period, relative to the total number of patients in the treatment group. Serious adverse events include any unexpected medical events that may result in death, life-threatening conditions, hospitalization or extended hospital stay, significant or sustained disabilities, or congenital abnormalities or defects.

2.14 Proportion of Patients with Non-Serious Adverse Events (%)

Definition: refers to the proportion of patients who experience non-serious adverse events during the treatment period, relative to the total number of patients in the treatment group. Non-serious adverse events are adverse events that do not meet the definition of serious adverse events.

**3 Outcomes for Healthcare Economics Evaluation**

3.1 Effectiveness outcomes in healthcare economics evaluation: The outcomes for efficacy in this study are effectiveness outcomes in health economics evaluation.

3.2 Quality-Adjusted Life Years (QALY)

Definition: QALY is calculated by multiplying the duration of survival in a particular health state (in days) by the health utility value during that period and dividing it by 365 days.

3.3 Health utility value

Definition: The health utility value is calculated using the official Chinese version of the EQ-5D-5L scale and the EQ-5D utility scoring system preferred by the Chinese population in mainland China

3.4 Cost-Effectiveness Ratio (CER)

Definition: the ratio of the cost (C) to the effectiveness (E) for each intervention.

3.5 Incremental Cost-Effectiveness Ratio (ICER)

Definition: ICER is the ratio of the difference in costs and effects between the intervention group and the control group, specifically the additional cost required to increase one Quality-Adjusted Life Year (QALY) in the intervention group compared to the control group. ICER is calculated as ICER = ΔC / ΔQALY

**痰热清注射液联合常规干预治疗慢性阻塞性肺疾病急性加重(AECOPD)痰热壅肺证临床评价的结局指标选取调查问卷**

**Questionnaire for the Selection of Outcome Measures for the Clinical Evaluation of Tan-re-qing Injection Combined with Conventional Interventions for Acute Exacerbation of Chronic Obstructive Pulmonary Disease (AECOPD)**

尊敬的专家：

Dear expert:

基于课题组前期对于国际与国内药物临床评价监管指导文件、国际与国内权威机构发布的COPD、AECOPD指南与专家共识、COMET网站中核心结局指标集（COS）制定研究以及中药注射液干预AECOPD的临床随机对照试验进行检索、梳理，结合焦点小组访谈结果，课题组提取出43个结局指标。

Based on the previous research conducted by our team on the international and domestic regulatory guidelines for drug clinical evaluation, guidelines and expert consensus on COPD and AECOPD published by authoritative institutions, the studies on the development of core outcome sets (COS) on the COMET website, and the clinical randomized controlled trials on Chinese herbal injections for AECOPD, our research team has extracted 43 outcome measures.

为确保结局指标的合理性，客观评价治疗的**有效性、安全性和卫生经济性**，我们诚挚邀请您对以下43个结局指标的重要程度从1到9（由低到高）进行打分，1-3分表示不重要，4-6分表示对COPD临床评价而言很重要但不关键，7-9分表示对COPD临床评价而言重要且关键。请在数字前的“□”处或数字上打“√”。

In order to ensure the rationality of the outcome measures and objectively evaluate the **effectiveness, safety, and cost-effectiveness of the treatment**, we sincerely invite you to rate the importance of the following 43 outcome measures on a scale of 1 to 9 (from low to high). A score of 1-3 indicates unimportant for clinical evaluation of AECOPD, 4-6 indicates importance but not critical, and 7-9 indicates important and critical. Please place a “√” or a check mark on the corresponding number.

评分时，每个结局指标的重要程度相互独立，同时请您补充您认为非常重要但未纳入的结局指标，以及任何对结局指标的修改建议。补充的指标可参考附件一“结局指标池”中的指标进行补充，感谢您的支持!

When scoring, please consider each outcome measure independently in terms of its importance. Additionally, please provide any additional outcome measures that you consider to be very important but are not included, as well as any suggestions for modifying the outcome measures. Supplementary measures can be referenced from the outcome indicators in Appendix 1 “Outcome Measures Pool”. Thank you for your support!

为了方便您查阅，点击附件名称可以直接链接到相关资料。

To facilitate your reference, clicking on the attachment name will directly link you to the relevant materials.

《痰热清注射液联合常规干预治疗AECOPD痰热壅肺证临床评价》课题组

The Research Team of “Clinical Evaluation of Tan-re-qing Injection Combined with Conventional Interventions for the Treatment of AECOPD”

2023年7月

July, 2023

**专家信息采集表**

**Expert Information Collection Form**

| 姓名 (Name) |  | 性别 (Sex) | |  |
| --- | --- | --- | --- | --- |
| 手机  (Telephone Number) |  | 单位/公司  (Institution/Company) | |  |
| 职称  (Professional Title) | □主任医师 (Chief Physician) | | | |
|  | □副主任医师 (Associate Chief Physician) | | | |
|  | □研究员 (Researcher) | | | |
|  | □副研究员 (Associate Researcher) | | | |
|  | □教授 (Professor) | | | |
|  | □副教授 (Associate Professor) | | | |
|  | □其他 (Others)___________ | | | |
| 工作年限  (Work experience) | □20年以上 (More than 20 years) | | 学历学位 (Academic degree) | □博士研究生  (PhD degree) |
|  | □10-20年 (10-20 years) | |  | □硕士研究生  (Master degree) |
|  | □5-10年 (5-10 years) | |  | □本科 (Bachelor degree) |
|  | □5年以下 (less than 5 years) | |  | □其他(others):___________ |
| 研究领域  (Research field) | □基础研究 (Basic research) | | | |
|  | □临床研究 (Clinical research) | | | |
|  | □循证医学研究 (Evidence-based medicine research) | | | |
|  | □临床流行病学研究 (Clinical epidemiology research) | | | |
|  | □其他 (Others):___________ | | | |

| **结局指标 (Outcomes)** | **重要性(Importance)** |
| --- | --- |
| **疗效评价指标 (Outcomes for Efficacy Evaluation)** | |
| 1. 治疗失败率（%）   定义：从治疗开始至随访12周时的治疗失败（TF），TF定义为以下3个终点的组合：（1）由于呼吸系统原因使用全身性皮质类固醇或机械通气进行的强化治疗；（2）加强住院治疗，包括转移至重症监护病房（ICU）或因呼吸原因再次入院；或（3）全因死亡（出现其中一个终点即认为治疗失败）。  The treatment failure rate (%)  Definition: defined as the combination of the following three endpoints from the start of treatment to the 12-week follow-up: (1) intensification treatment with systemic corticosteroids or mechanical ventilation due to respiratory reasons, (2) intensified hospitalization, including transfer to the intensive care unit (ICU) or readmission due to respiratory reasons, or (3) all-cause mortality. The occurrence of any of these endpoints is considered treatment failure. | □ 1 □ 2 □ 3 □ 4  □ 5 □ 6 □ 7 □ 8 □ 9 |
|  | |
| 1. 住院时间   定义：从治疗开始至患者出院的天数。  Length of hospital stay: Definition: The number of days from the start of treatment to the patient’s discharge from the hospital. | □ 1 □ 2 □ 3 □ 4  □ 5 □ 6 □ 7 □ 8 □ 9 |
| 1. 再入院率（%）   定义：患者治疗出院后，随访期24周内再次因COPD或相关并发症入院的人数占该组总人数的比例。  Hospital readmission rate (%): Definition: The proportion of patients who are readmitted due to COPD or related complications within a 24-week follow-up period after treatment discharge, in relation to the total number of patients in the group. | □ 1 □ 2 □ 3 □ 4  □ 5 □ 6 □ 7 □ 8 □ 9 |
|  | |
| 1. AECOPD发作频次（次）   定义：患者治疗出院后，随访期24周内再次发作AECOPD的次数。  Frequency of AECOPD exacerbations (occurrences): Definition: The number of times a patient experiences a recurrence of AECOPD within a 24-week follow-up period after treatment discharge. | □ 1 □ 2 □ 3 □ 4  □ 5 □ 6 □ 7 □ 8 □ 9 |
| 1. 再次AECOPD时长（天）   定义：患者治疗出院后，至随访期24周内首次再发AECOPD的天数。  Duration of recurrent AECOPD (days): Definition: The number of days from treatment discharge until the first occurrence of AECOPD within a 24-week follow-up period. | □ 1 □ 2 □ 3 □ 4  □ 5 □ 6 □ 7 □ 8 □ 9 |
|  | |
| 1. 转入ICU治疗患者比例（%）   定义：患者治疗期间，需转入ICU治疗的患者人数占该组总人数的比例。  Proportion of patients requiring ICU treatment (%): Definition: The proportion of patients in a group who require transfer to the Intensive Care Unit (ICU) for treatment during their treatment period. This is measured by the number of patients who require ICU treatment divided by the total number of patients in the group. | □ 1 □ 2 □ 3 □ 4  □ 5 □ 6 □ 7 □ 8 □ 9 |
| 1. ICU治疗时长（天）   定义：患者治疗期间，在ICU治疗的总天数。  Duration of ICU treatment (days): Definition: The total number of days that a patient receives treatment in the Intensive Care Unit (ICU) during their treatment period. | □ 1 □ 2 □ 3 □ 4  □ 5 □ 6 □ 7 □ 8 □ 9 |
| 1. 有创机械通气患者比例（%）   定义：患者治疗期间，需要有创机械通气患者的比例。  Proportion of patients receiving invasive mechanical ventilation (%): Definition: The proportion of patients in a treatment group who require invasive mechanical ventilation during their treatment period, expressed as a percentage. | □ 1 □ 2 □ 3 □ 4  □ 5 □ 6 □ 7 □ 8 □ 9 |
| 1. AECOPD相关并发症发生率（%）   定义：患者治疗期间，发生AECOPD相关并发症的患者人数占该组总人数的比例。  Proportion of patients with AECOPD-related complications (%): Definition: The proportion of patients in a treatment group who experience AECOPD-related complications during their treatment period. | □ 1 □ 2 □ 3 □ 4  □ 5 □ 6 □ 7 □ 8 □ 9 |
|  | |
| 1. 退热时长（小时）   定义：患者治疗期间，从体温超过37.3℃到体温降到37.3℃的时长  Duration of fever (hours): Definition: The length of time it takes for a patient’s temperature to decrease from above 37.3℃ to 37.3℃ during their treatment period. | □ 1 □ 2 □ 3 □ 4  □ 5 □ 6 □ 7 □ 8 □ 9 |
|  |  |
| 1. CAT量表（The COPD Assessment Test）得分（量表见附件二）   CAT score (The COPD Assessment Test) (See Appendix 2 for the questionnaire) | □ 1 □ 2 □ 3 □ 4  □ 5 □ 6 □ 7 □ 8 □ 9 |
| 1. EQ-5D生存质量量表中文版（量表见附件三）   Score of Chinese version of EQ-5D Quality of Life Scale (See Appendix 3 for the questionnaire) | □ 1 □ 2 □ 3 □ 4  □ 5 □ 6 □ 7 □ 8 □ 9 |
| 1. HRQOL-5量表得分（量表见附件四）   HRQOL-5 scale score (see Appendix 4 for the questionnaire) | □ 1 □ 2 □ 3 □ 4  □ 5 □ 6 □ 7 □ 8 □ 9 |
| 1. 慢性阻塞性肺疾病急性加重期证候疗效评价量表（AECOPD-STES）得分（量表见附件五）   Score of the Acute Exacerbation of Chronic Obstructive Pulmonary Disease Symptoms and Treatment Efficacy Scale (AECOPD-STES) (see Appendix 5 for the questionnaire) | □ 1 □ 2 □ 3 □ 4  □ 5 □ 6 □ 7 □ 8 □ 9 |
|  | |
| 1. 白细胞计数（cells/µL）   定义：血常规检测中的白细胞计数  White Blood Cell Count (cells/µL) Definition: The measurement of white blood cells in a complete blood count | □ 1 □ 2 □ 3 □ 4  □ 5 □ 6 □ 7 □ 8 □ 9 |
| 1. C-反应蛋白浓度（mg/L）   定义：血浆C-反应蛋白浓度。  C-Reactive Protein Concentration (mg/L) Definition: The concentration of C-reactive protein in the plasma. | □ 1 □ 2 □ 3 □ 4  □ 5 □ 6 □ 7 □ 8 □ 9 |
| 1. 白细胞介素-6（IL-6）（pg/mL）   定义：血液中白细胞介素-6浓度。  Interleukin-6 (IL-6) Level (pg/mL)  Definition: The concentration of Interleukin-6 (IL-6) in the blood. |  |
| 1. 白细胞介素-8（IL-8）（pg/mL）   定义：血液中白细胞介素-8浓度。  Interleukin-8 (IL-8) Level (pg/mL)  Definition: The concentration of Interleukin-6 (IL-6) in the blood. |  |
| 1. PaO_2_水平（mmHg）   定义：动脉血气分析中的PaO_2_水平。  PaO_2_ Level (mmHg) Definition: The level of PaO_2_ in arterial blood gas analysis | □ 1 □ 2 □ 3 □ 4  □ 5 □ 6 □ 7 □ 8 □ 9 |
| 1. PaCO_2_水平（mmHg）   定义：动脉血气分析中的PaCO_2_水平。  PaCO_2_ Level (mmHg) Definition: The level of PaCO_2_ in arterial blood gas analysis | □ 1 □ 2 □ 3 □ 4  □ 5 □ 6 □ 7 □ 8 □ 9 |
|  | |
| 1. FEV_1_占最大预测值百分比（%）   定义：肺功能检测中，FEV1（用力呼气一秒容积）占最大预测值百分比。  Forced expiratory volume in one second predicted（FEV_1_%） Definition: In pulmonary function testing, the FEV1 (forced expiratory volume in one second) as a percentage of the predicted maximum value. | □ 1 □ 2 □ 3 □ 4  □ 5 □ 6 □ 7 □ 8 □ 9 |
| 1. 用力肺活量（FVC）（L）   定义：肺功能检测中，一个人在最大力气下从最大吸气到最大呼气的完整呼吸周期中能够排出的空气总量。  Forced Vital Capacity (FVC) (L) Definition: In pulmonary function testing, the total volume of air that a person can forcefully exhale during a complete respiratory cycle, starting from maximum inhalation to maximum exhalation under maximal effort. | □ 1 □ 2 □ 3 □ 4  □ 5 □ 6 □ 7 □ 8 □ 9 |
| 1. FEV_1_/FVC（%）   定义：用力呼气一秒容积（FEV1）与用力肺活量（FVC）的比值。  FEV_1_/FVC Ratio (%) Definition: The ratio of forced expiratory volume in one second (FEV_1_) to forced vital capacity (FVC) in pulmonary function testing. | □ 1 □ 2 □ 3 □ 4  □ 5 □ 6 □ 7 □ 8 □ 9 |
| 1. 降钙素原（PCT）（ng/mL）   定义：血液中降钙素原的浓度。  Procalcitonin (PCT) Level (ng/mL) Definition: The concentration of procalcitonin in the blood. | □ 1 □ 2 □ 3 □ 4  □ 5 □ 6 □ 7 □ 8 □ 9 |
| 1. 耐药菌出现率（%）   定义：痰培养及药敏试验中，出现耐药菌的人数占该组总人数的比例。  Prevalence of Drug-Resistant Bacteria (%) Definition: In sputum culture and antibiotic susceptibility testing, the proportion of individuals with drug-resistant bacteria to the total number of individuals in that group. | □ 1 □ 2 □ 3 □ 4  □ 5 □ 6 □ 7 □ 8 □ 9 |
| 1. 耐药菌耐药数量（个）   定义：痰培养及药敏试验中，出现耐药菌，采集耐药菌耐药的数量。  Number of Drug-Resistant Bacteria (Count) Definition: In sputum culture and antibiotic susceptibility testing, when drug-resistant bacteria are identified, the number of strains resistant to specific antibiotics is recorded and the count of resistant bacteria is determined. | □ 1 □ 2 □ 3 □ 4  □ 5 □ 6 □ 7 □ 8 □ 9 |
| **安全性评价指标 (Outcomes for Safety Evaluation)** | |
| 1. 使用抗菌素时间（天）   定义：从首次应用抗生素到首次停用抗菌素所需时间。  Duration of Antibiotic Use (days) Definition: The amount of time required from the first administration of an antibiotic to the first discontinuation of antibiotic use. | □ 1 □ 2 □ 3 □ 4  □ 5 □ 6 □ 7 □ 8 □ 9 |
| 1. 抗菌素由静脉改为口服时间（天）   定义：从静脉应用抗菌素到改为口服抗菌素所需天数。  Transition Time from Intravenous to Oral Antibiotics (days) Definition: Refers to the number of days required from the initial administration of antibiotics via the intravenous route to the transition to oral antibiotic therapy. | □ 1 □ 2 □ 3 □ 4  □ 5 □ 6 □ 7 □ 8 □ 9 |
| 1. 抗菌素由广谱改为窄谱时间（天）   定义：从抗菌素由广谱改为窄谱所需天数。  Transition Time from Broad-Spectrum to Narrow-Spectrum Antibiotics (days) Definition: Refers to the number of days required for the transition from broad-spectrum antibiotic therapy to narrow-spectrum antibiotic therapy. | □ 1 □ 2 □ 3 □ 4  □ 5 □ 6 □ 7 □ 8 □ 9 |
| 1. 抗菌素累积剂量（mg）   定义：患者治疗期间，累计使用抗菌素的剂量。  Cumulative Dose of Antibiotics (mg)  Definition: The cumulative dose of antibiotics used during the patient’s treatment period. | □ 1 □ 2 □ 3 □ 4  □ 5 □ 6 □ 7 □ 8 □ 9 |
|  | |
| 1. 皮质类固醇激素累积剂量（mg）   定义：患者治疗期间，累计使用皮质类固醇激素的剂量。  Cumulative Dose of Corticosteroids (mg)  Definition: The cumulative dose of corticosteroids used during the patient’s treatment period. | □ 1 □ 2 □ 3 □ 4  □ 5 □ 6 □ 7 □ 8 □ 9 |
| 1. 皮质类固醇激素使用天数（天）   定义：患者治疗期间，累计使用皮质类固醇激素的天数。  Duration of Corticosteroid Use (days)  Definition: The cumulative duration of corticosteroid usage during the patient’s treatment period. | □ 1 □ 2 □ 3 □ 4  □ 5 □ 6 □ 7 □ 8 □ 9 |
|  | |
| 1. 严重不良事件发生率（%）   定义：患者治疗期间，发生严重不良事件的患者例数占该组总人数的比例。严重不良事件指任何非预期的医疗事件，可导致死亡、危及生命、需要住院或延长住院、持续或重大残疾或无行为能力、先天性异常或先天性缺陷。  Proportion of Patients with Serious Adverse Events (%) Definition: Refers to the proportion of patients who experience serious adverse events during the treatment period, relative to the total number of patients in the treatment group. Serious adverse events include any unexpected medical events that may result in death, life-threatening conditions, hospitalization or extended hospital stay, significant or sustained disabilities, or congenital abnormalities or defects. | □ 1 □ 2 □ 3 □ 4  □ 5 □ 6 □ 7 □ 8 □ 9 |
| 1. 非严重不良事件发生率（%）   定义：患者治疗期间，发生非严重不良事件的患者例数占该组总人数的比例。非严重不良事件指不满足严重不良事件定义的不良事件。  Proportion of Patients with Non-Serious Adverse Events (%) Definition: Refers to the proportion of patients who experience non-serious adverse events during the treatment period, relative to the total number of patients in the treatment group. Non-serious adverse events are adverse events that do not meet the definition of serious adverse events. | □ 1 □ 2 □ 3 □ 4  □ 5 □ 6 □ 7 □ 8 □ 9 |
| 1. 肺炎进展率（%）   定义：患者治疗期间，发生肺炎进展患者例数占该组总人数的比例。肺炎进展定义为通过胸部CT线检查出现新的实变。  Proportion of Patients with Pneumonia Progression (%) Definition: Refers to the proportion of patients who experience pneumonia progression during the treatment period, relative to the total number of patients in the treatment group. Pneumonia progression is defined as the presence of new consolidations observed through chest CT imaging. | □ 1 □ 2 □ 3 □ 4  □ 5 □ 6 □ 7 □ 8 □ 9 |
|  | |
| 1. 尿常规异常率（%）   定义：患者治疗期间，发生尿常规异常患者例数占该组总人数的比例。尿常规包含外观、pH值、比重、蛋白质、葡萄糖、酮体、亚硝酸盐、潜血、白细胞酯酶、细胞计数与形态、细菌与真菌、结晶与颗粒检测。  Proportion of Patients with Abnormal Urinalysis (%) Definition: Refers to the proportion of patients who have abnormal results in urinalysis during the treatment period, relative to the total number of patients in the treatment group. Urinalysis includes evaluations of appearance, pH level, specific gravity, protein, glucose, ketones, nitrites, occult blood, leukocyte esterase, cellular count and morphology, bacteria and fungi, as well as crystal and particle detection. | □ 1 □ 2 □ 3 □ 4  □ 5 □ 6 □ 7 □ 8 □ 9 |
| 1. 凝血功能异常率   定义：患者治疗期间，发生凝血功能检测异常患者例数占该组总人数的比例。凝血功能检测包含凝血酶原时间（PT）、活化部分凝血活酶时间（APTT）、凝血酶时间（TT）、纤维蛋白原、D-二聚体、血小板计数和功能。  Proportion of Patients with Abnormal Coagulation Function Definition: Refers to the proportion of patients who have abnormal results in coagulation function tests during the treatment period, relative to the total number of patients in the treatment group. Coagulation function tests include prothrombin time (PT), activated partial thromboplastin time (APTT), thrombin time (TT), fibrinogen, D-dimer, platelet count, and platelet function. | □ 1 □ 2 □ 3 □ 4  □ 5 □ 6 □ 7 □ 8 □ 9 |
| 1. 肝功能异常率   定义：患者治疗期间，发生肝功能检测异常患者例数占该组总人数的比例。肝功能检测包含肝酶学检测：天门冬氨酸转氨酶（AST）、丙氨酸转氨酶（ALT）、碱性磷酸酶（ALP）、γ-谷氨酰转肽酶（GGT），血清总蛋白与白蛋白水平、血清胆红素与直接胆红素水平。  Proportion of Patients with Abnormal Liver Function Definition: Refers to the proportion of patients who have abnormal results in liver function tests during the treatment period, relative to the total number of patients in the treatment group. Liver function tests include hepatic enzyme tests: aspartate aminotransferase (AST), alanine aminotransferase (ALT), alkaline phosphatase (ALP), and gamma-glutamyl transferase (GGT), as well as serum levels of total protein and albumin, and serum levels of bilirubin and direct bilirubin. | □ 1 □ 2 □ 3 □ 4  □ 5 □ 6 □ 7 □ 8 □ 9 |
| 1. 肾功能异常率   定义：患者治疗期间，发生肾功能检测异常患者例数占该组总人数的比例。肾功能检测包含血清肌酐（Serum Creatinine）、血尿素氮（Blood Urea Nitrogen, BUN）、尿素/肌酐比值（Urea/Creatinine Ratio）、尿酸（Uric Acid）、血清尿酸清除率（Serum Uric Acid Clearance）。  Proportion of Patients with Abnormal Renal Function Definition: Refers to the proportion of patients who have abnormal results in renal function tests during the treatment period, relative to the total number of patients in the treatment group. Renal function tests include measurements such as serum creatinine, blood urea nitrogen (BUN), urea/creatinine ratio, uric acid, and serum uric acid clearance. These tests are used to assess the kidney’s ability to filter waste products from the blood and maintain proper fluid and electrolyte balance in the body. | □ 1 □ 2 □ 3 □ 4  □ 5 □ 6 □ 7 □ 8 □ 9 |
| **卫生经济性评估** **（Outcomes for Healthcare Economics Evaluation）** | |
| 1. 效果指标：本研究的疗效评价指标为效果指标   Effectiveness outcomes in healthcare economics evaluation: The outcomes for efficacy in this study are effectiveness outcomes in health economics evaluation. | □ 1 □ 2 □ 3 □ 4  □ 5 □ 6 □ 7 □ 8 □ 9 |
| 1. 成本-效果比（CER）   定义：成本效果比即每个项目的成本（C）与效果（E）的比值。  Cost-Effectiveness Ratio (CER)  Definition: the ratio of the cost (C) to the effectiveness (E) for each intervention | □ 1 □ 2 □ 3 □ 4  □ 5 □ 6 □ 7 □ 8 □ 9 |
| 1. 效用指标：质量调整生命年（QALY）   定义：QALY = 该健康状态下生存的时间长度（天数）乘以这段时间内的健康效用值/365天  Quality-Adjusted Life Years (QALY)  Definition: QALY is calculated by multiplying the duration of survival in a particular health state (in days) by the health utility value during that period and dividing it by 365 days. | □ 1 □ 2 □ 3 □ 4  □ 5 □ 6 □ 7 □ 8 □ 9 |
| 1. 健康效用值   定义;采用中国大陆EQ-5D-5L量表官方中文版及于中国人群偏好的EQ-5D量表效用值积分体系计算  Health utility value  Definition: The health utility value is calculated using the official Chinese version of the EQ-5D-5L scale and the EQ-5D utility scoring system preferred by the Chinese population in mainland China | □ 1 □ 2 □ 3 □ 4  □ 5 □ 6 □ 7 □ 8 □ 9 |
| 1. 增量成本效用比（ICER）   定义：干预与对照组成本和效用之差的比值，即干预组比对照组每多提高一个质量调整生命年所需要的成本，ICER=ΔC/ΔQALY。  Incremental Cost-Effectiveness Ratio (ICER)  Definition: ICER is the ratio of the difference in costs and effects between the intervention group and the control group, specifically the additional cost required to increase one Quality-Adjusted Life Year (QALY) in the intervention group compared to the control group. ICER is calculated as ICER = ΔC / ΔQALY | □ 1 □ 2 □ 3 □ 4  □ 5 □ 6 □ 7 □ 8 □ 9 |
| 其他修改或补充建议 (Other modifications or additional suggestions) |  |

实践经验、理论分析、国内外相关文献、直观感觉这四个判断依据在您对上述条目的重要性评价中起到了何种程度的作用（小、中、大）

To what extent do practical experience, theoretical analysis, relevant literature from domestic and international sources, and intuitive feeling contribute to your evaluation of the importance of the aforementioned items (low, medium, high)?

|  | 小 (Low) | 中 (Medium) | 大 (High) |
| --- | --- | --- | --- |
| 实践经验 (practical experience) | ○ | ○ | ○ |
| 理论分析(theoretical analysis) | ○ | ○ | ○ |
| 国内外相关文献 (relevant literature from domestic and international sources) | ○ | ○ | ○ |
| 直观感觉 (intuitive feeling) | ○ | ○ | ○ |

| 您对以上条目的熟悉程度  (The familiarity levels for the given options) |
| --- |
| ○不熟悉 (Not familiar) ○不太熟悉 (Not too familiar) ○一般熟悉 (Moderately familiar) ○较熟悉 (Quite familiar) ○很熟悉 (Very familiar) |
